# Exploring the role of normalization and feature selection in microbiome disease classification pipelines

**DOI:** 10.1093/gigascience/giaf096

**Published:** 2025-09-02

**Authors:** Ignacio Garach Vélez, Francisco Manuel Ortuño Guzmán, Ignacio Rojas Ruiz, Luis Javier Herrera Maldonado

**Affiliations:** Department of Computer Engineering, Automation and Robotics (ICAR), University of Granada, 18071 Granada, Spain; Department of Computer Engineering, Automation and Robotics (ICAR), University of Granada, 18071 Granada, Spain; Department of Computer Engineering, Automation and Robotics (ICAR), University of Granada, 18071 Granada, Spain; Department of Computer Engineering, Automation and Robotics (ICAR), University of Granada, 18071 Granada, Spain

**Keywords:** microbiome, normalization, machine learning, feature selection, classification

## Abstract

**Background:**

Disease classification using 16S rRNA microbiome data faces challenges of high dimensionality, compositionality, and sparsity, compounded by the inherent small sample sizes in many studies. Machine learning and feature selection techniques offer potential to identify robust biomarkers and improve classification performance; however, their comparative effectiveness across diverse methods and datasets has been insufficiently explored. This study evaluates multiple feature selection techniques alongside normalization strategies, focusing on their interplay with classifier performance.

**Results:**

Our analyses revealed that centered log-ratio normalization improves the performance of logistic regression and support vector machine models and facilitates feature selection, whereas random forest models yield strong results using relative abundances. Interestingly, presence–absence normalization was able to achieve similar performance compared to abundance-based transformations across classifiers. Among feature selection methods, minimum redundancy maximum relevancy (mRMR) surpassed most methods in identifying compact feature sets and demonstrated performance comparable to least absolute shrinkage and selection operator (LASSO), which obtained top results requiring lower computation times. Autoencoders needed larger latent spaces to perform well and lacked interpretability, Mutual Information suffered from redundancy, and ReliefF struggled with data sparsity.

**Conclusions:**

Overall, feature selection pipelines improved model focus and robustness via a massive reduction of the feature space. mRMR and LASSO emerged as the most effective methods across datasets.

## Background

Microbial communities are present in almost every part of the human body, from the mouth to the gut, skin, and reproductive tract. The gut microbiome, widely recognized as the most extensively studied microbial community, has attracted significant attention in recent years. Numerous studies have linked microbiota dysbiosis to the development of different health problems [1], not only infectious diseases but also cancer [2], inflammatory disorders such as Crohn’s disease [3] or arthritis [4], and even neurodegenerative disorders such as Parkinson’s due to the gut–brain axis [5].

Although numerous studies employing classical statistical methods have been conducted to demonstrate these connections, the rapid advancement of next-generation sequencing technologies and the availability of increasingly larger, higher-quality datasets with more diverse cohorts underscore the potential of the use of machine learning techniques in these studies. These new approaches offer opportunities to uncover complex patterns and associations that might be challenging to detect using traditional statistics, potentially leading to more refined insights and robust predictive models.

The task of detecting the presence of a disease in a host from the gut microbiome has been approached in various ways [1]. Machine learning methods and, more specifically, the supervised learning paradigm enable researchers to address this disease classification problem from microbiome data with optimal prediction accuracy, helping to identify key biomarkers and therapeutic targets [6].

16S rRNA microbiome data present several inherent challenges that complicate their application in disease classification tasks. First, the high dimensionality of these datasets, with hundreds or thousands of features (operational taxonomic units [OTUs] or amplicon sequence variants [ASVs]) representing bacterial species, often greatly exceeds the number of available samples, leading to the classic curse of the dimensionality problem [7]. Additionally, the sparse nature of these data [8], where most taxa have very low or absent abundances in many samples, increases the chance of overfitting and risks model generalization. Moreover, microbiome data are compositional [9], meaning the abundances of taxa are proportions that sum to 1 (or any read number if not under closure operation). This fact introduces important dependencies between features that can harm machine learning (ML) algorithms and must be properly accounted for, in order to avoid misleading conclusions. These challenges emphasize the need for normalization and advanced techniques that respect the unique characteristics of microbiome data [10]. In this research, we compare the performance of models using relative abundances and various normalization techniques, as well as evaluate the effect of applying rarefaction of counts prior to normalization.

**Figure 1:**
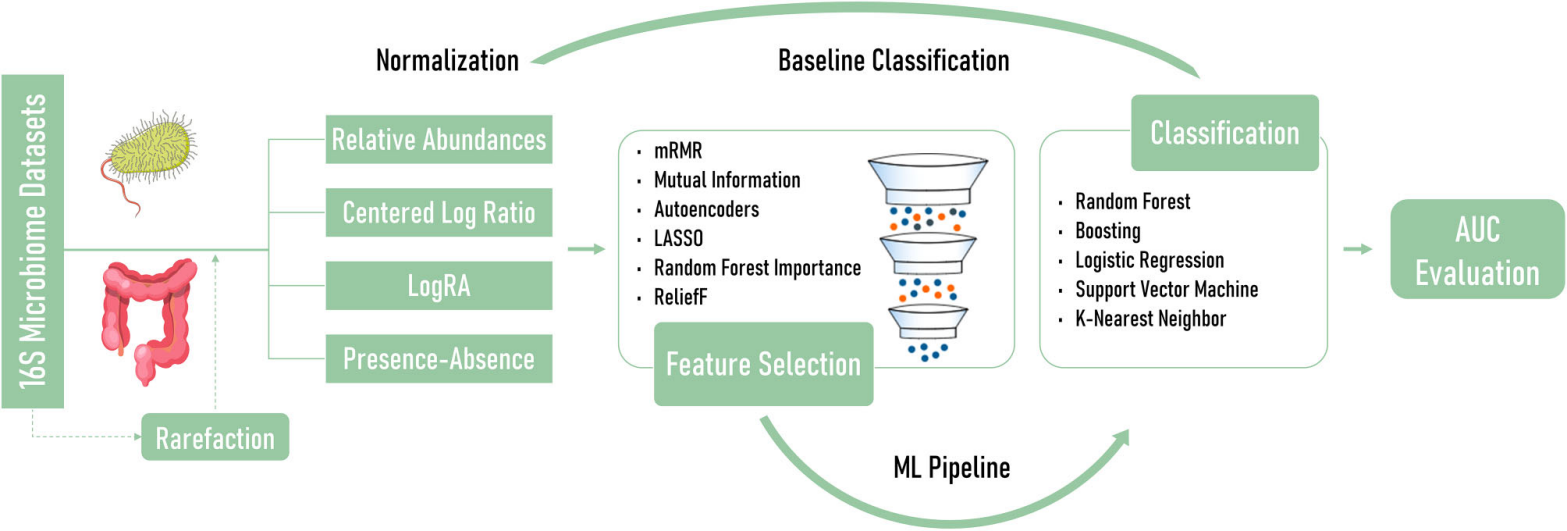
Graphical abstract. Overview of the proposed pipeline, illustrating the normalization strategies, feature selection techniques, and classification algorithms evaluated.

Moreover, feature selection arises as a great tool for microbiome data analysis challenges, allowing us to find small taxa signatures and helping classifiers to deal with the high-dimensional compositional-sparse data, ideally improving performance. In this article, our aim is to evaluate multiple feature selection methods and their interaction with classifiers to identify robust microbial features across diseases using a large collection of 16 rRNA disease classification datasets. Feature selection has been used to address this problem with some cohorts [11, 12], but a systematic comparison in multiple datasets such as the one presented here is lacking. This offers a broader understanding of feature selection and model interactions in the field of 16S microbiome data modeling. A summary of the analysis pipeline and key methodological components is provided in Fig. 1 as a graphical abstract.

## Data Description

Although some big initiatives have been developed to centralize microbiome data studies such as The Human Microbiome Project [13] or The American Gut Project [14], big curated repositories with metadata enabling further study of disease classification problems are lacking. To compare the performance of our pipelines, we leveraged 16S gut datasets from MicrobiomeHD, a standardized database of human gut 16S microbiome case-control studies and their associated patient metadata [15], and MLrepo, a repository of curated microbiome-related supervised learning tasks [16]. We selected datasets containing at least 75 samples and a minimum 1:6 imbalance ratio between cases and controls. To enhance the benchmark, we retrieved 2 more gut datasets online with abundance tables and metadata available [17]. Multiple datasets from MicrobiomeHD that exhibited extremely low performance in preliminary tests (an area under the receiver operating characteristic curve [AUC] lower than 0.60) were excluded from the final analysis to avoid biases in the interpretation of the results. Similarly, datasets with excessively high performance (AUC greater than 0.95) were also excluded, as their classification tasks were too simple, offering limited variability and failing to provide meaningful differences between algorithms. The final selected datasets and their respective original studies are available in Table 1. A total of 3,320 gut samples from 15 datasets were considered.

**Table 1:** Benchmark datasets used in our classification analysis, including their imbalance ratios (IRs) and references

Dataset	Samples	Features	IR	Reference
ART	114 (86, 28)	10,733	3.07	[18]
CDI	336 (93, 243)	3,456	2.61	[19]
CRC1	490 (229, 261)	6,920	1.14	[20]
CRC2	102 (46, 56)	837	1.22	[21]
HIV	350 (293, 57)	14,425	5.14	[22]
CD1	140 (78, 62)	3,547	1.26	[23]
CD2	160 (68, 92)	3,547	1.35	[23]
IBD1	91 (67, 24)	2,742	2.79	[24]
IBD2	114 (68, 46)	1,496	1.48	[25]
CIR	77 (51, 26)	3,104	1.96	[26]
MHE	77 (26, 51)	3,104	1.96	[26]
OB	281 (220, 61)	6,386	3.61	[27]
PAR1	148 (74, 74)	10,232	1.00	[28]
PAR2	333 (201, 132)	6,844	1.52	[29]
PAR3	507 (323, 184)	12,198	1.76	[30]

ART: Arthritis; CDI: *Clostridium difficile* Infection; CRC1 and CRC2: Colorectal Cancer; HIV: Human Immunodeficiency Virus; CD1 and CD2: Crohn’s Disease; IBD1 and IBD2: Inflammatory Bowel Disease; CIR: Cirrhosis; MHE: Minimal Hepatic Encephalopathy; OB: Obesity; PAR1, PAR2, and PAR3: Parkinson’s Disease.

CD1 and CD2 were taken from MLRepo, PAR2 and PAR3 were retrieved from their respective reference, and the remaining datasets were obtained from MicrobiomeHD.

## Analyses

Empirical evidence is presented on the performance levels of baseline ML models across 16S microbiome datasets, focusing on the impact of normalization techniques and feature selection methods. Validation AUC, derived from a nested cross-validation procedure, is used as the primary metric to assess model performance. The models are implemented using the scikit-learn library [31], ensuring consistency and reproducibility. Hyperparameter tuning is done at the inner loop of the validation. The parameter combinations considered are available in Table 2.

**Table 2:** Hyperparameter grid for classifiers used in the study

Classifier	Hyperparameter	Parameters
Random Forest	n_estimators	[200, 300, 400]
	max_features	[sqrt, log2]
	max_depth	[None, 3, 5, 7, 8]
KNN	n_neighbors	[7, 9, 11, 13, 15, 17, 19, 21]
	weights	[uniform, distance]
SVM	C	[0.001, 0.1, 1, 10, 100, 1,000]
	kernel	[‘rbf’]
	gamma	[scale, auto]
Logistic Regression	C	np.logspace(-4, 4, 20)
Boosting	max_depth	[3, 5, 7, 8]
	n_estimators	[300, 500, 800]

### Baseline classification and influence of normalization

To perform an initial assessment of the predictive power of microbiome features for the different diseases under different normalization strategies, we trained and validated 5 ML models (random forest [RF], XGBoost (XGB), logistic regression [LR], support vector machine [SVM], and k-nearest neighbor [KNN]) on each dataset. We used relative abundance data and then compared the results with the same models applied to data transformed using 3 different normalization techniques: centered log-ratio (CLR), log-transformed relative abundance (logRA), and presence–absence (PA) (see Methods section for details). Rarefaction was also assessed prior to normalization to evaluate its effect on performance.

These models represent a diverse set of classification strategies. Random forest and XGBoost are tree-based ensemble methods known for their robustness and ability to capture complex nonlinear relationships. Logistic regression is a linear model that provides interpretable coefficients and performs well when the data are linearly separable. Support vector machine is a margin-based classifier effective in high-dimensional spaces, particularly suitable for sparse data. Finally, k-nearest neighbor is a lazy learning method that classifies samples based on similarity to their neighbors, though it can be sensitive to noise and sparsity in the data. A more detailed description of each classifier is provided in the Methods section.

**Figure 2:**
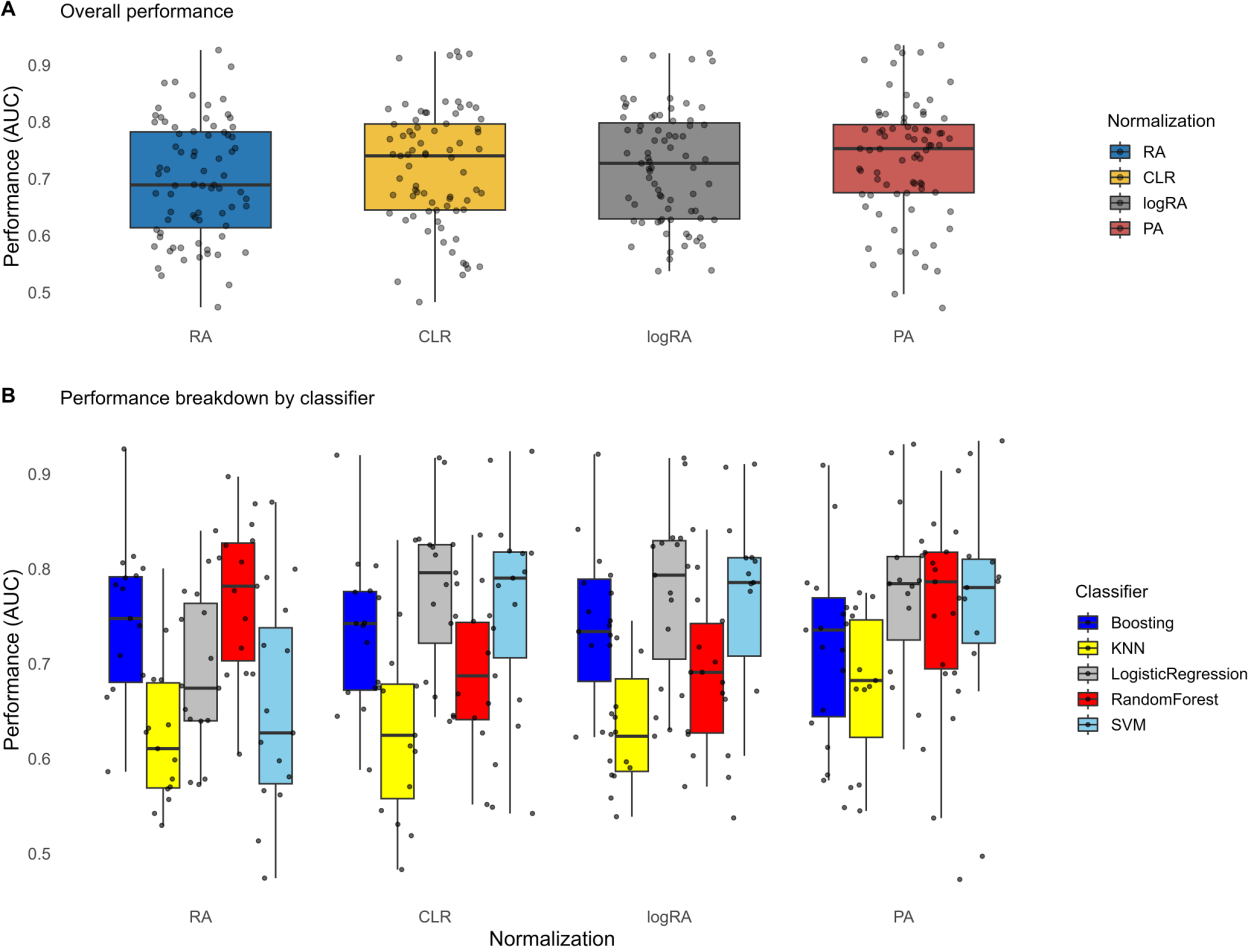
Overall normalization performance and breakdown by classifier for the 4 types of normalizations—baseline classification.

On our initial analysis, we compared the overall performance of the 4 normalization strategies. As Fig. 2A displays, the distribution of AUC scores is similar across these methods. This was statistically confirmed by a global Friedman test, which yielded a nonsignificant $p_{val}=0.56$.

Then, to explore the results more in depth, we proceeded with a more granular analysis, breaking down the performance by classifier for each normalization method individually to uncover more specific trends (see Fig. 2B).

**Figure 3:**
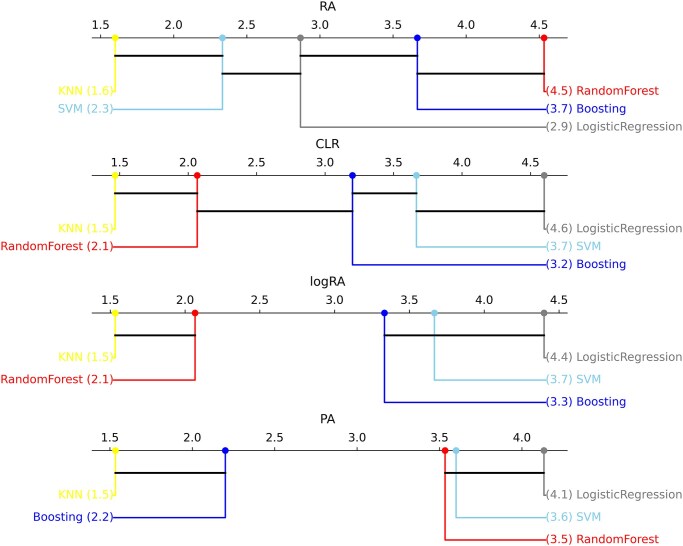
Finner’s test result critical distance diagrams for each normalization.

Focusing on the relative abundance (RA) approach, trends on classifier performance showed that tree-based models obtained best results across most datasets, with random forest yielding the best AUC on average. Boosting (XGBoost) obtained the best performance on the CDI dataset, with an AUC of 0.926. Friedman’s multiple comparisons test revealed statistical differences across algorithms, and Finner’s post hoc results shown in Fig. 3 confirmed this ranking, with random forest results significantly different when compared with all algorithms except for boosting. K-nearest neighbors obtained the worst results, while not being statistically different from support vector machine. Sitting in the middle ground, logistic regression obtained marginally better performance than SVM and slightly worse than boosting.

Next, focusing on data normalized using the CLR transformation, trends clearly changed, with Friedman’s test again showing significant differences in performance. In this case, best models were logistic regression and support vector machines. Boosting stays one step below as a stable alternative, and random forest results dramatically fall into the fourth ranking position. One notable example of this drop in performance with normalization is the Obesity dataset, which obtained an AUC of 0.869 for relative abundances and 0.788 for CLR normalization (note that logistic regression and SVM improved RA results for this dataset).

Finally, focusing on data normalized using the remaining 2 transformations, logRA and PA, the Friedman test also revealed significant differences between classifiers in both cases. For the logRA normalization, results were extremely similar to those from CLR. Logistic regression models achieved the best average ranks, followed by support vector machines and boosting. Unlike what was observed with CLR normalization, in this case, boosting models did not show statistically significant differences in performance compared to logistic regression and SVM. Random forest and KNN fell behind, being significantly outperformed by the top models.

In contrast with abundance-based transformations, the PA normalization showed higher performance in a larger range of different models, with logistic regression, SVM, and random forest ranking high and with no significant statistical differences between them. However, the boosting and KNN results were not comparable to the top ones, even though KNN worked better compared with the other normalizations.

On average, best-performing models were random forest for RA data and logistic regression for CLR and logRA normalizations. PA obtained similar performance to the best abundance-based normalization pipelines when using a random forest or logistic regression classifier (see again AUC boxplots in Fig. 2 and Finner’s test results in Fig. 3). AUC values varied between datasets, showing which ones are more challenging (see Supplementary Tables S3–S6). The CDI dataset achieved an AUC of 0.897 using random forest with RA and 0.917 using logistic regression with CLR. In contrast, the arthritis ART dataset had an AUC of only 0.605 with random forest using RA and 0.644 with logistic regression using CLR.

**Figure 4:**
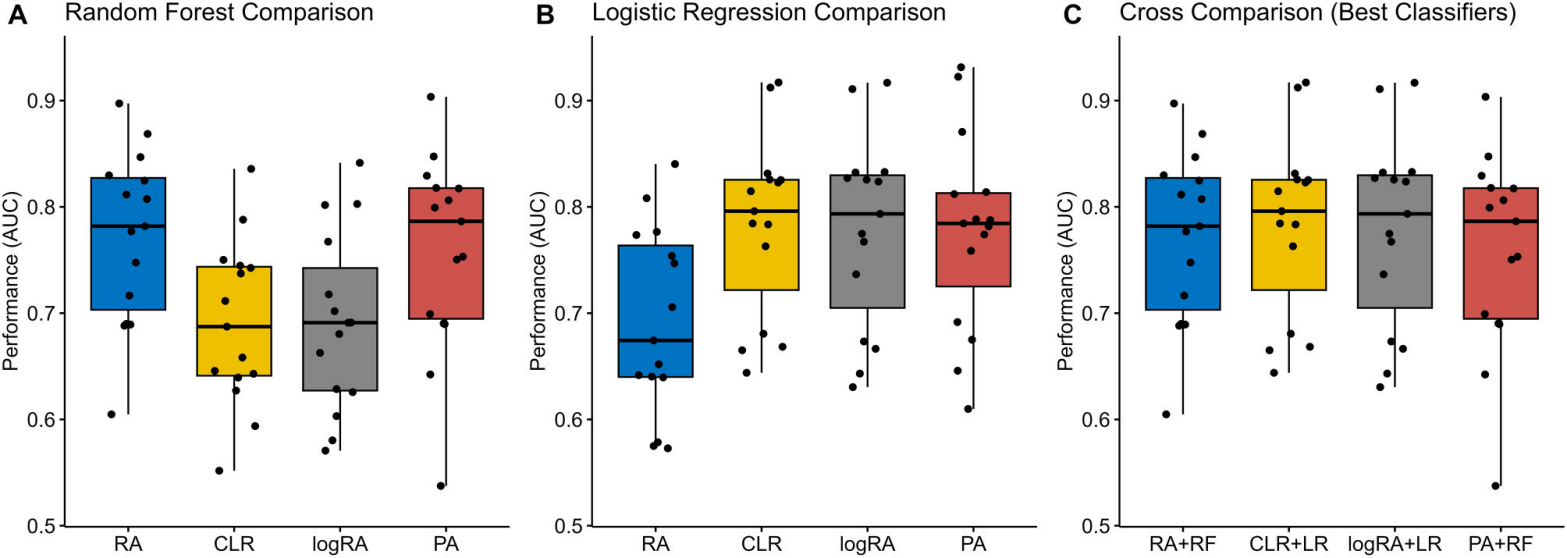
(A) Random forest comparison through normalization methods. Friedman’s test with *P*  $= 1.1 \cdot 10^{-5}$. (B) Logistic regression comparison through normalization methods. Friedman’s test with *P*  $= 1.3 \cdot 10^{-4}$. (C) Comparing best-performing classifiers for each normalization. Friedman’s test with *P*  $= 0.34$.

We finally carried out a comparison between best models with and without normalization using Friedman ranks test. As Fig. 4 shows, significant differences were found between normalizations. It can be observed that the logistic regression comparison on both normalization methods revealed superior performance with CLR and logRA (Fig. 4B) and the opposite trend in the random forest comparison, with RA and PA obtaining better results (Fig. 4A). The PA results were robust and comparable to the best normalizations in both comparisons.

To further investigate, we compared the best-performing models for each normalization RA + random forest, CLR + logistic regression, logRA + logistic regression, and PA + random forest. Friedman’s test did not reveal differences $p_{val}=0.34$ (C), suggesting different ways classifiers can be adapted to deal with compositional data.

An additional analysis was conducted to evaluate the impact of rarefaction and its interactions with normalization methods on classification performance. Rarefaction involves subsampling the count data to a uniform sequencing depth across samples. This is typically done using the minimum library size observed in the dataset. However, when certain samples are extremely shallow, this approach can result in a substantial loss of information. To overcome this, we included a second variant where the rarefaction depth was set to the 5th percentile of each dataset sample depths, removing samples with fewer reads than this quantity.

In the global comparison, Wilcoxon paired tests revealed statistically significant differences between nonrarefied data and both rarefaction approaches, with all *P* values indicating strong significance in the detriment of rarefaction (specifically, $p = 4.4 \cdot 10^{-13}$ for standard rarefaction and $p = 9.3 \cdot 10^{-5}$ for Q05 rarefaction). Quantile-modified rarefaction also performed significantly better than standard rarefaction $p = 1.5 \cdot 10^{-9}$.

When examining each normalization method individually, consistent patterns can be observed (see Supplementary Fig. S1). For RA and CLR normalization, nonrarefied data significantly outperformed both rarefied counterparts. In contrast, no differences were found for logRA and PA normalization for quantile-based rarefaction, though it still did not yield any improvements. Interestingly, for CLR, logRA, and PA, the quantile-based rarefaction yielded significantly better results than standard rarefaction (see Supplementary Table S2).

Therefore, rarefaction was not applied in the subsequent pipeline analyses involving feature selection, as it consistently led to either inferior or nonimproved results across normalization methods.

### Feature selection performance comparison

We also examined the predictive power of the feature set rankings provided by the different ML feature selection techniques. We considered Mutual Information, minimum redundancy maximum relevancy (mRMR), least absolute shrinkage and selection operator (LASSO), RelieF, random forest importances, and autoencoders (see Table 8 and Methods section).

Table 3 shows the top 5 performing feature selection + classifier pipelines per normalization method, illustrating that the ones involving logistic regression outperformed the other methods. Thus, we observe that trends in classifier performance with CLR and logRA normalizations persisted after feature selection, with logistic regression the best-performing method across datasets. Furthermore, random forest continues to be the best classifier for RA data even after feature selection. Additionally, PA stability across classifiers is also maintained, yielding strong results using both LR and RF as feature selection (FS) pipeline classifiers.

**Table 3:** Top 5 machine learning pipelines (classifier + feature selection) sorted by mean AUC across datasets for each normalization method

RA		CLR		logRA		PA	
Pipeline	Mean AUC	Pipeline	Mean AUC	Pipeline	Mean AUC	Pipeline	Mean AUC
RF + RF100	0.761	LR + mRMR100	0.772	LR + mRMR100	0.766	RF + mRMR100	0.757
RF + mRMR100	0.757	LR + LASSO	0.768	LR + LASSO	0.763	LR + mRMR100	0.753
RF + RF50	0.750	LR + mRMR50	0.764	LR + mRMR50	0.762	RF + RF100	0.752
RF + ReliefF100	0.745	SVM + LASSO	0.749	SVM + LASSO	0.751	LR + AE100	0.752
RF + ReliefF100	0.744	LR + AE100	0.744	SVM + mRMR100	0.748	RF + LASSO	0.750

**Figure 5:**
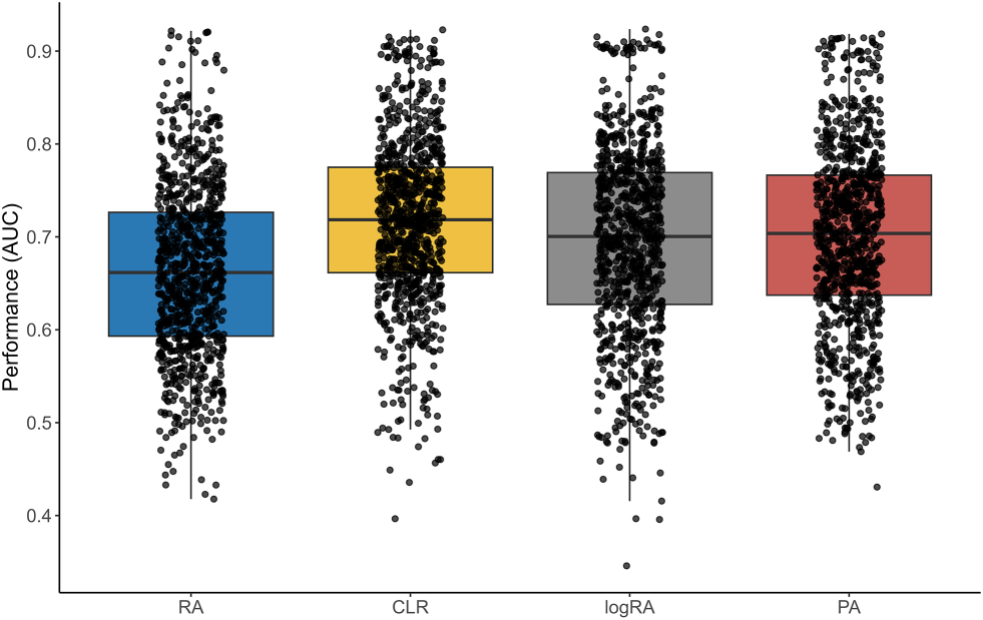
Boxplot of all possible Feature Selection + Classifier pipelines for each normalization technique.

**Figure 6:**
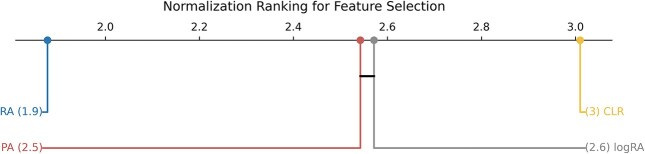
Critical difference diagram from Finner’s post hoc test on feature selection results for each normalization.

In our search for the best ML pipeline, we executed all combinations of normalization techniques with the selected feature selection methods and classifiers. A total of 900 models were validated for each normalization method, and to observe effects of normalization on feature selection, we performed Friedman’s test, which showed significant differences between groups $p = 1.9 \cdot 10^{76}$. A boxplot showing the results across normalizations is shown in Fig. 5. As can be observed, CLR normalization outperformed all other methods overall. Moreover, pipelines relying on relative abundance data consistently underperformed in comparison to all normalization-based approaches. These findings are further supported by the Finner post hoc test results shown in Fig. 6, where logRA and PA occupy an intermediate position, being the only pair of normalization methods with no statistically significant differences between them.

Similarly, to select the most suitable classifier, we performed multiple comparison tests. The results, detailed in the Supplementary Fig. S2, revealed that logistic regression was statistically superior to the other evaluated models. The previously presented Table 3, which highlighted the top-performing pipelines for the CLR normalization, further corroborates this choice, with LR consistently appearing among the best models.

Relying on these results, we selected CLR + logistic regression as the reference to compare the feature selection algorithms on the normalized data, simplifying the analysis and enabling a more focused evaluation of their effectiveness.

**Figure 7:**
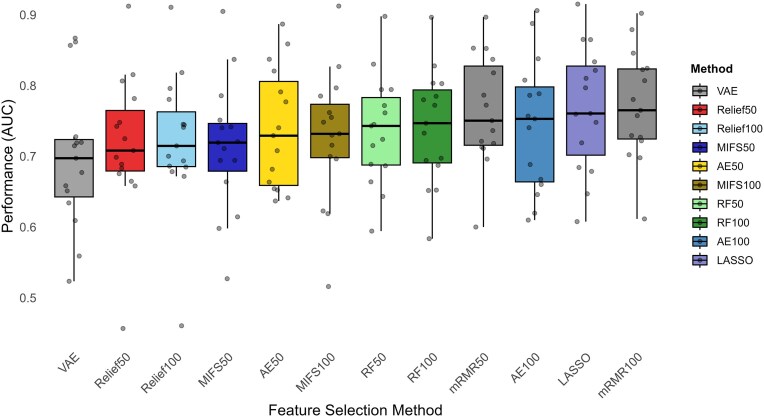
CLR + feature selection + logistic regression performance comparison.

**Figure 8:**
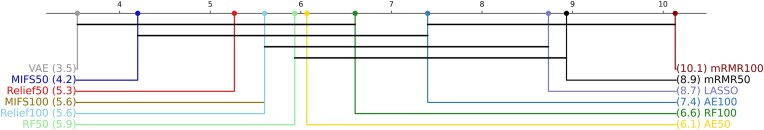
Finner’s test result critical distance diagram for feature selection + logistic regression pipelines with CLR normalization. Ranks from Friedman’s test with *P*  $= 8.6 \cdot 10^{-7}$.

Figure 7 and Table 4 show results of nested validation from this analysis. As expected, Friedman’s multiple comparisons test revealed statistical differences with high significance $p_{val} = 8.6 \cdot 10^{-7}$. Two groups of methods are revealed in terms of results. The first group includes 3 algorithms with good performance at a comparable level: LASSO, mRMR50, and mRMR100, which consistently yield stronger results across datasets. On the other hand, the second group comprises 7 algorithms with lower mean performance, including ReliefF50, ReliefF100, variational autoencoder, MIFS50, MIFS100, RF50, and AE50, with the last one a step above the others. Finally, AE100 and RF100 performances place them in a middle spot between the groups. Critical distance diagram in Fig. 8 illustrates how Finner’s post hoc test confirms these groups.

**Table 4:** Feature selection + logistic regression cross-validation results for data normalized with the centered log-ratio transformation

Dataset	AE50	AE100	VAE	ReliefF50	ReliefF100	MIFS50	MIFS100	mRMR50	mRMR100	LASSO	RF50	RF100
CDI	0.8869	0.8878	0.8617	0.9123	0.9107	0.9049	0.9124	0.8968	0.9022	**0.9152**	0.8979	0.8965
CIR	0.6542	0.6602	0.5233	0.7479	0.7410	0.6942	0.7318	**0.8371**	0.8245	0.8103	0.7429	0.7468
CRC1	0.6817	0.6675	0.6584	0.6830	0.6849	0.6144	0.6189	**0.7112**	0.7022	0.6470	0.6639	0.6514
CRC2	**0.8206**	0.7862	0.7188	0.7249	0.7452	0.7403	0.7550	0.7504	0.7575	0.7606	0.7451	0.7799
CD1	0.7770	0.7885	0.7277	0.7424	0.7445	0.7857	0.7852	0.7865	**0.8069**	0.7968	0.7944	0.8028
CD2	0.8372	0.8380	**0.8569**	0.8154	0.8184	0.8371	0.8269	0.8525	0.8459	0.8215	0.8303	0.8279
HIV	0.7079	0.7404	0.6092	0.6987	0.6936	0.7194	0.7299	0.7364	0.7270	**0.8336**	0.6866	0.6872
IBD1	0.7911	0.8077	0.7148	0.8065	0.7960	0.7512	0.7619	0.8180	0.8225	**0.8653**	0.7944	0.7849
IBD2	0.6413	0.6197	0.6512	0.6647	0.6783	0.6942	0.6998	0.6961	0.6979	0.6842	**0.7240**	0.6942
MHE	0.6523	0.6884	0.6771	0.4566	0.4604	0.7110	0.7157	0.7183	**0.7651**	0.7191	0.6888	0.6973
OB	0.8589	**0.9060**	0.8671	0.7817	0.7805	0.7417	0.7967	0.8529	0.8791	0.8650	0.7718	0.8035
PAR1	0.6636	0.6459	0.6341	0.6888	0.7002	0.5981	0.6227	0.7130	**0.7293**	0.6787	0.6432	0.6522
PAR2	0.7291	**0.7529**	0.7197	0.6754	0.6861	0.6638	0.6964	0.7213	0.7219	0.7480	0.7152	0.7328
PAR3	0.7403	0.7569	0.6973	0.7081	0.7147	0.7206	0.7482	0.7724	**0.7804**	0.7596	0.7615	0.7722
ART	0.6368	0.6098	0.5590	0.6581	**0.6717**	0.5270	0.5159	0.6000	0.6115	0.6077	0.5943	0.5835

Bold values represent the best performing feature selector for each dataset.

The 2 variants of the minimum redundancy maximum relevance algorithm (50 and 100) achieved the highest AUC scores on a large number of datasets. Small differences were found when varying the number of selected features, indicating that fewer than 50 features may be sufficient for some datasets. Finner’s test showed significant differences between them and their Mutual Information feature selection (MIFS) counterparts. Results on some datasets like Obesity or CD1 improve when using top 100 features, suggesting a more detailed analysis should be done when building a model with the mRMR algorithm.

MIFS selection achieves moderate results in feature selection but, as expected, tends to suffer from significant redundancy, with MIFS100 not being significantly better than MIFS50 (see Fig. 8).

LASSO-based feature selection emerges as a competitive alternative to mRMR, achieving robust AUC scores (the highest in CDI, HIV, and IBD1) that are very close to the best-performing method, with no significant differences found compared with mRMR.

Relief-based methods do not perform particularly well on these datasets, except for some interesting exceptions. In the CDI and Arthritis (ART) datasets, they obtained top results, with some differences compared with other feature selection methods in the case of Arthritis ($AUC=0.672$).

Feature selectors based on RF importance scores display intermediate performance in this comparative. Selecting the top 100 features (RF100) results in a slight performance improvement compared to selecting only 50 features (RF50). The statistical analysis via Finner’s test highlights that RF’s performance is not significantly different from that of competitive methods like mRMR50 and LASSO. Concurrently, RF does not demonstrate a statistically significant advantage over several of the lower-performing methods, including the ReliefF and Mutual Information variants.

Autoencoder results place them in a situation analogous to that of random forest, positioned in a middle ground in terms of performance. In particular, Finner’s test did not show significant differences for AE100, neither compared with the top-performing methods nor with some of the weaker ones, such as ReliefF50 and MIFS50. When constrained to a latent space of 50 dimensions (AE50), performance was less favorable. Finally, variational autoencoders (VAEs) displayed very poor performance on most datasets.

For the sake of completeness, we also analyzed the feature selection performance using all normalizations and classifier combinations. The top results are similar to those of CLR logistic regression, with mRMR100, mRMR50, and LASSO performing the best. The Friedman test confirmed significant differences between groups, and the Finner post hoc test (Supplementary Fig. S3) indicated that mRMR100 was still the top-performing method. Additionally, in this global comparison, random forest importances and ReliefF selectors performed significantly better than autoencoders across normalizations. Finally, VAE, MIFS50, and MIFS100 exhibited even weaker performance in this comparison.

#### mRMR selection outperforms Mutual Information

**Figure 9:**
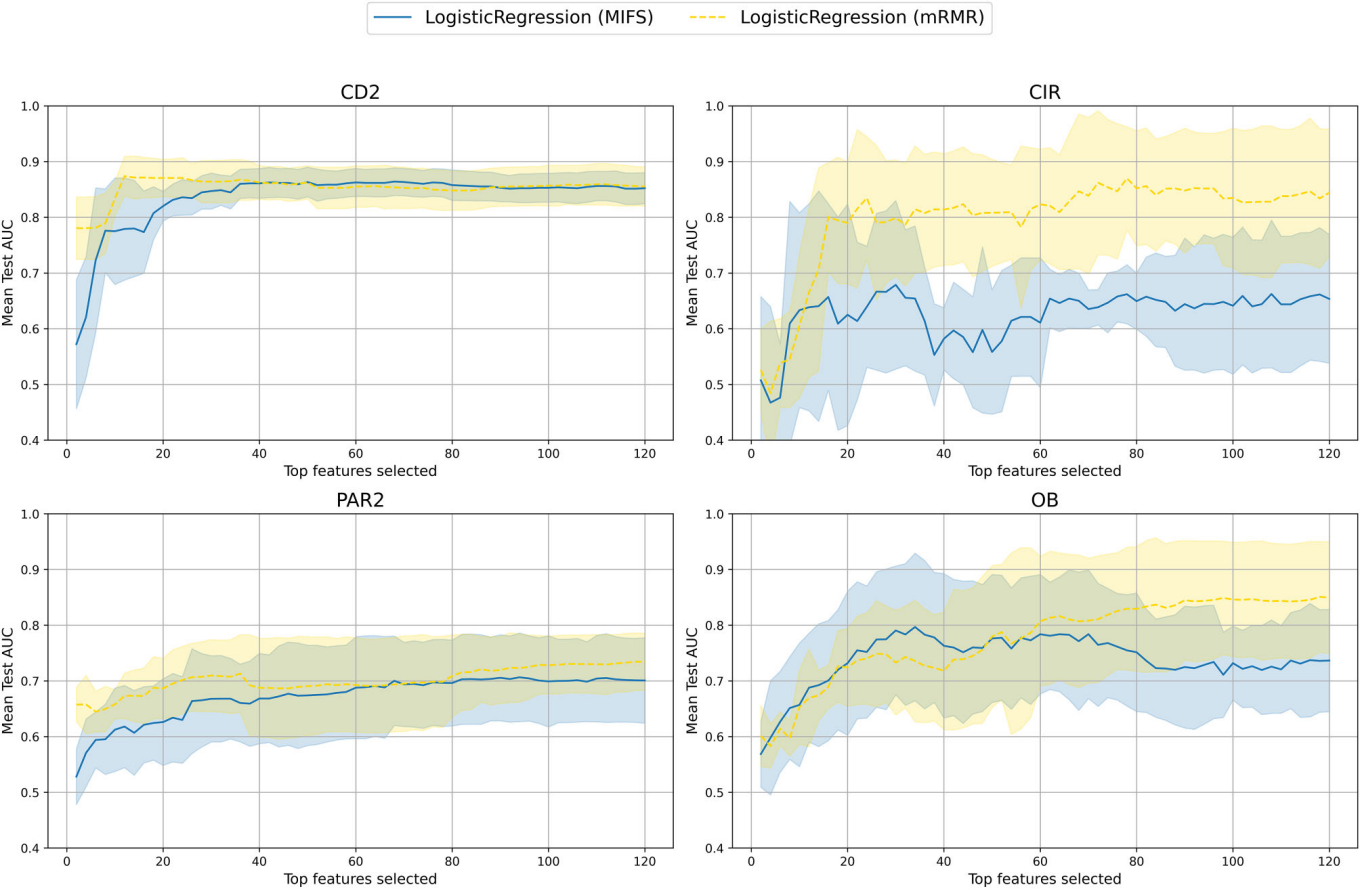
Cross-validation of logistic regression models with incremental number of features provided by Mutual Information and mRMR. Datasets CD2 and CIR provide examples of optimal feature sets with fewer than 50 features. PAR2 and OB sets keep improving beyond 50 and 100 features.

Motivated by the observation that mRMR100 was the best-performing method in our global comparison, we conducted a focused analysis of mRMR for its potential to extract smaller and more interpretable predictive feature sets. We compared mRMR with Mutual Information (MI) for feature selection, employing an incremental approach where 2 new variables from the feature selection ranking were added at a time to evaluate their contributions (see Fig. 9).

The results on most datasets demonstrated that mRMR consistently outperforms MI in identifying relevant features, with mRMR-selected features yielding better performance. This incremental approach is aligned with our statistical analysis, which showed significant differences between mRMR and MI for both small (50 features) and larger (100 features) subsets.

Two different trends are shown in Fig. 9. CD2 and CIR plots illustrate that in some scenarios, fewer than 50 features are enough to obtain near-optimal sets, while PAR2 and OB model performance keeps improving when adding more than 50 features.

To illustrate the practical output of the feature selection process, we display taxa selected by the mRMR algorithm for dataset PAR3 in Table 5. The method identified the most informative bacterial taxa after CLR transformation that are most relevant for the classification task. Notably, the selected taxa exhibit a high diversity of genera.

**Table 5:** Top 12 taxa ranking provided by mRMR feature selection after CLR for the PAR3 dataset

Phylum	Class	Order	Family	Genus
Firmicutes	Clostridia	Clostridiales	Lachnospiraceae	*Roseburia*
Firmicutes	Clostridia	Clostridiales	Family_XI	*Ezakiella*
Firmicutes	Bacilli	Bacillales	Not assigned	Not assigned
Firmicutes	Clostridia	Clostridiales	Ruminococcaceae	*Butyricicoccus*
Firmicutes	Clostridia	Clostridiales	Lachnospiraceae	*Lachnoclostridium*
Firmicutes	Bacilli	Lactobacillales	Streptococcaceae	*Streptococcus*
Proteobacteria	Gammaproteobacteria	Enterobacteriales	Enterobacteriaceae	*Escherichia/Shigella*
Proteobacteria	Gammaproteobacteria	Betaproteobacteriales	Burkholderiaceae	*Delftia*
Firmicutes	Clostridia	Clostridiales	Ruminococcaceae	*Ruminococcaceae*_UCG-013
Firmicutes	Bacilli	Lactobacillales	Streptococcaceae	*Streptococcus*
Actinobacteria	Actinobacteria	Bifidobacteriales	Bifidobacteriaceae	*Bifidobacterium*
Firmicutes	Clostridia	Clostridiales	Lachnospiraceae	*Lachnospiraceae*_UCG-004

#### LASSO performance remarks

Classic LASSO feature selection has also been applied to 16S compositional data, with CLR normalization the most straightforward preprocessing approach [32]. As we showed before, relative abundances did not fit well in this method. Our results show that this method achieves comparable performance across datasets against more complex feature selection algorithms. No statistical differences were found with well-performing methods like mRMR (see post hoc test results in Fig. 8).

However, LASSO tends to select a larger number of features compared to methods like mRMR, particularly in larger datasets. As shown in Table 6, the number of selected features by LASSO is higher in datasets with more samples. This raised the need to verify whether using only the top 50 or top 100 features selected by LASSO would be sufficient to approach the best performance.

**Table 6:** Number of features selected by LASSO (mean of the cross-validation), sorted by number of features of the dataset

Dataset	Selected features	Features	Samples
CRC2	60.32	837	$102 (46,56)$
IBD2	71.12	1,496	$114 (68,46)$
IBD1	56.28	2,742	$91 (67,24)$
CD1	70.32	3,547	$140 (78,62)$
CD2	75.60	3,547	$160 (68,92)$
CDI	115.88	3,456	$336 (93, 243)$
CIR	47.88	3,104	$77 (51,26)$
MHE	51.00	3,104	$77 (26,51)$
OB	126.44	6,386	$281 (220,61)$
CRC1	217.68	6,920	$490 (229,261)$
PAR2	188.72	6,844	$333 (201,132)$
PAR1	100.52	10,232	$148 (74,74)$
ART	75.32	10,733	$114 (86, 28)$
PAR3	295.96	12,198	$507 (323,184)$
HIV	143.84	14,425	$350 (293,57)$
Mean	**113.13**		

The implementation we used takes $10^{-5}$ as the threshold to consider the absolute importance of a feature to be shrunk to zero and not selected. To assess the influence of this parameter, we repeated the analysis, taking only the 50 and 100 most important variables, instead of all the variables not shrunk to zero. Results in Supplementary Table S7 show that differences between the threshold approach and the top 100 approach are minimal. Even selecting the top 50 features is enough to achieve similar results for most datasets considered.

We also measured computing time performance for feature selection algorithms (see Supplementary Fig. S4). Random forest importances and LASSO stand out as the quickest, efficiently handling large datasets. Mutual Information and VAE also performed swiftly. In contrast, algorithms like mRMR and autoencoders fall into an intermediate speed category; they were slower than LASSO but still manageable for moderate datasets. On the other hand, ReliefF was noticeably slower, specially with larger sample sizes.

### Parkinson cross-cohort generalization

In order to assess the proposed pipelines on microbiome datasets and further see their operation in identifying microbial signatures on even external datasets, we used datasets PAR2 and PAR3, which targeted the same hypervariable region. However, their dimensionality differed, with 6,844 features in PAR2 and 12,198 in PAR3. To address this difference, we trained models using the 2,360 features shared between them. While both datasets were collected following uniform protocols, the analysis performed in [29] revealed batch effects between datasets. Thus, to avoid confounding, we did not mix datasets while training; instead, we trained the pipelines in one dataset and tested their generalization score in the other in a cross-cohort scheme.

**Figure 10:**
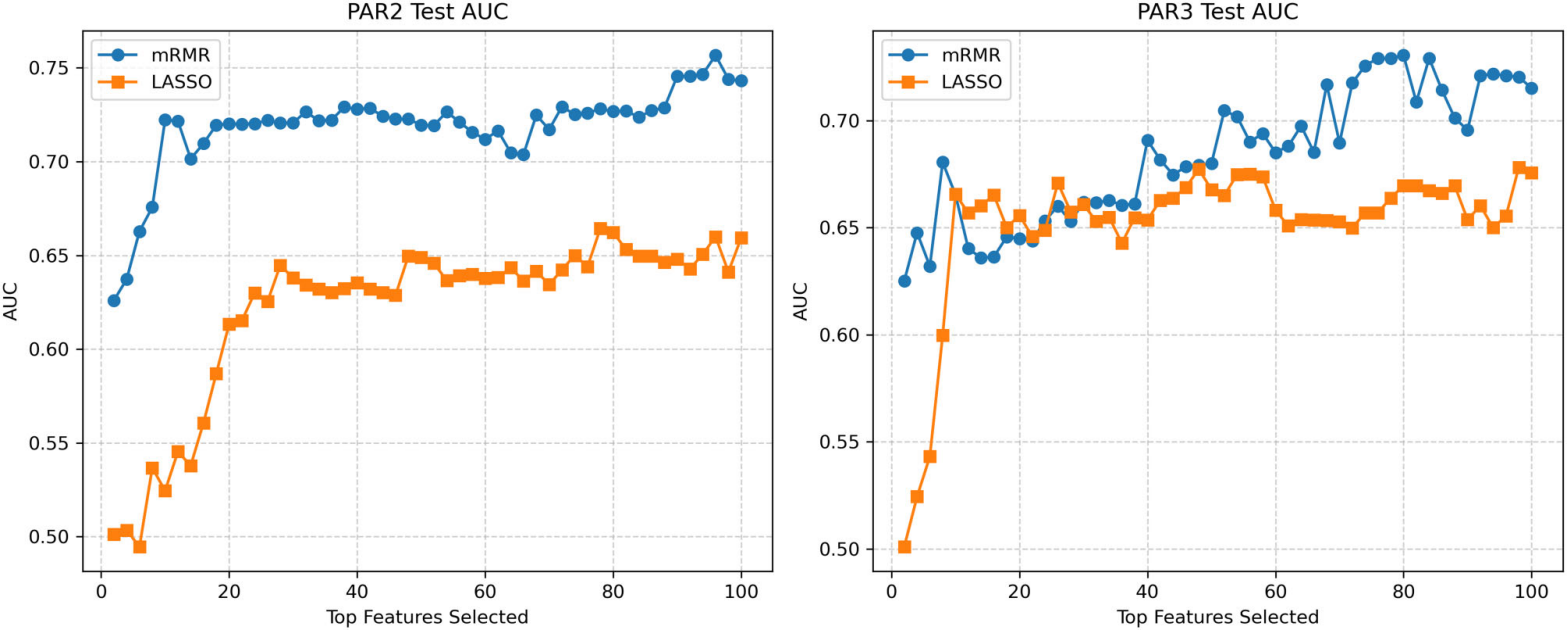
Cross-cohort performance comparison for Parkinson datasets (PAR2 and PAR3). Logistic regression models were trained in 1 dataset and tested in the other. This plot shows the test AUCs considering top features incrementally.

We independently trained logistic regression models with feature selection using mRMR and LASSO on the subset of 2,360 intersectional features. We performed an incremental analysis, evaluating classification performance by sequentially adding 2 features at a time (see Fig. 10). Comparing the performance of features selected by mRMR and LASSO revealed that mRMR-selected features exhibited better generalization in this case study.

Pipelines trained with dataset PAR3 and tested in PAR2 achieved an AUC of 0.722 with only 10 microbial markers, reaching an AUC of 0.743 with 100 for the mRMR + LR pipeline. However, LASSO + LR only reached an AUC of 0.659 with the top 100 selected features. Conversely, pipelines trained with dataset PAR2 and tested in PAR3 achieved a similar performance for mRMR and LASSO with the top 50 features. Again, when considering 100 features, the mRMR + LR pipeline obtained an AUC of 0.715, and LASSO + LR only obtained an AUC of 0.675.

## Discussion

Normalization methods play a crucial role in microbiome data analysis, influencing the performance of classification tasks. A recent study showed that CLR normalization consistently outperforms RA for logistic regression models when classifying microbiome data [33]. Our findings corroborate this result for logistic regression, with CLR normalization yielding superior performance over RA across datasets. Similarly, but with slightly worse performance, SVM exhibited the same behavior across normalization methods. However, we proved this trend does not generalize across all classifiers. The global comparison, regardless of the classifier chosen, shows that while specific classifier performance varies with normalization, the overall impact across models selected is minimal.

The focused evaluation per normalization revealed that random forest performs exceptionally well with RA while performing poorly with CLR and logRA normalizations. This result underscores the importance of tailoring normalization methods to specific algorithms, as different models exhibit varying sensitivities to the transformed data distribution. The good performance of random forest using relative abundance data is aligned with the comparative conducted in [34], where CLR transformation was not applied. A plausible explanation is that decision trees, the core components of random forest and boosting, operate by splitting data with decision thresholds, making them less affected by the compositional and interdependent nature of the data.

Moreover, the comparison between best classifiers for each normalization technique did not reveal a big impact on performance. This suggests that while method-specific normalization optimizations can enhance individual classifier outcomes, the overall performance remains stable across different approaches for baseline disease classification.

Boosting and KNN displayed stable performance across normalization methods while they did not achieve top results in any of them. KNN generally underperformed relative to other classifiers. This result is likely attributable to the sparsity inherent in microbiome data, which poses challenges for distance-based algorithms like KNN. This underperformance is not addressed properly by any normalization (best performance was obtained with PA), as the root issue seems to lie in the high sparsity of the dataset rather than the compositional nature of the data.

Additionally, the stability of PA across classifiers is remarkable. Our results are aligned with [35] and [36] studies, where PA normalization yielded performance comparable to that of abundance-based transformations.

On the other hand, rarefaction, even in its quantile-based variant, generally leads to decreased classification performance, likely due to information loss from discarding potentially relevant counts. This could be particularly concerning in datasets from domains like the reproductive microbiome, where highly dominant species [37] coexist with low-abundance taxa that may be lost during rarefaction. Nevertheless, the improved performance of quantile-based rarefaction compared to the standard rarefaction approach may suggest a mild outlier removal effect, excluding poorly sequenced samples that differ from the overall trend of the dataset.

While these findings highlight the critical interplay between normalization strategies and machine learning algorithms, they also pave the way for further analysis. CLR transformation was found to enable a better-performing feature selection. This enhancement in performance can be attributed to the way CLR transformation addresses the compositional issue. Data are transformed from the simplex into a hyperplane in the Euclidean space, which makes unconstrained statistical methods applicable [38], thus likely making data more suitable to feature selection methods that implicitly use these techniques.

When comparing feature selection methods, results shown in Table 4 and the critical distance diagram (Fig. 8) state clearly that mRMR and LASSO obtained top performance on most datasets. Notably, mRMR50 ranked slightly higher than LASSO while using fewer features on average.

### mRMR selection obtains compact predictive sets

The superior performance of mRMR can be attributed to the balance of its design that generates more compact sets of informative features, which in some scenarios can be smaller, as evidenced by the incremental analysis we conducted (Fig. 9). These findings highlight mRMR’s value as a robust and interpretable feature selection method for key attribute discovery in complex microbiome datasets.

A notable example of mRMR’s effectiveness is demonstrated in the dataset PAR3, where the method identified a highly informative subset of fewer than 50 genera. These features exhibited strong associations with the target condition, achieving comparable performance to larger feature sets selected by other methods. Furthermore, these findings are consistent with results reported in the original study by [29], which highlighted the significance of reduced abundance of the genera *Roseburia* and *Butyricicoccus*, as well as the family Lachnospiraceae, in Parkinson’s disease cases, alongside an observed overabundance of *Bifidobacterium*. All of these bacteria were found in the top 12 rankings provided by mRMR (Table 5).

This result highlights the power of mRMR in focusing on the most relevant taxa, while minimizing redundancy coming from the hierarchical relationships between species of bacteria, thus resulting in compact biologically meaningful sets of features. The interpretability of these subsets is particularly valuable for translational applications, as it provides clear targets for further biological or clinical validation.

### Autoencoders struggle to trade off performance and feature reduction

During experimentation, we tested various autoencoder architectures to determine the optimal configuration for microbiome feature selection. AE100 performed reasonably well, ranking highly on some datasets. However, the modest results obtained by AE50 indicated that larger latent spaces are required to achieve superior classification performance. However, this improvement would came at the cost of reduced interpretability and the necessity of handling a high-dimensional latent representation.

Additionally, we experimented with a VAE architecture, which is designed to encode input data into a probabilistic latent space. Despite its theoretical advantages, such as generating smoother latent spaces and capturing underlying data distributions, the VAE did not perform well in our datasets. It failed to effectively model the complexity of the microbiome data, resulting in worse classification outcomes compared to traditional autoencoders.

A significant limitation of autoencoders, particularly in the context of microbiome research and medical applications, is their inherent lack of interpretability. The latent space representations they generate are often considered “black boxes,” making it difficult to directly link features to biological phenomena or actionable insights. While some methods for interpreting autoencoder outputs exist, they remain limited in scope and application [39, 40].

This lack of transparency poses a challenge, especially in health care–related fields where decision-making algorithms are increasingly scrutinized for explainability [41].

Taking into account these considerations, autoencoders may not be the most suitable alternative for identifying biologically meaningful signature sets. Simpler and more transparent methods, such as mRMR and LASSO, may be better suited for this specific task.

### LASSO remains as a powerful option

Lasso feature selection has been shown to perform well in microbiome-based disease classification paired with logistic regression and random forest for inflammatory bowel disease [42, 43]. Our results are aligned with these studies, showing that this method achieves comparable performance across datasets against more complex feature selection algorithms.

Overall, the mRMR + LR and LASSO + LR pipelines (with CLR) proved to be powerful strategies for improving the robustness and quality of the models. Table 7 shows that these feature selection pipelines achieve at least similar performance, if not better, than the best baseline classification models. Moreover, feature selection offers a greater advantage, a massive reduction of the dimensionality. As we can see, a reduction of around 99% of the features can be achieved, and the selected ones are able to display similar predictive performance to using all of them. This reduction helps models to reduce overfitting and avoid learning noise or spurious patterns coming from features that are irrelevant to the biological signal. Additionally, a reduced set of features makes it easier to interpret and understand the underlying factors influencing the model’s predictions.

**Table 7:** AUC performance comparison of different pipelines on microbiome datasets. For each dataset, the highest AUC is displayed in bold. The 2 best baseline combinations are listed first, followed by the top 3 complete pipelines combining feature selection and logistic regression. Reduction (%) indicates the percentage of features discarded by the feature selection method, relative to the total number of input features.

Dataset	Input Features	Baseline		CLR + LASSO + LR		CLR + mRMR100 + LR		CLR + mRMR50 + LR	
		RA + RF	CLR + LR	AUC	*Reduction (%)*	AUC	*Reduction (%)*	AUC	*Reduction (%)*
CDI	3456	0.8973	**0.9171**	0.9152	*96.65*	0.9022	*97.11*	0.8968	*98.55*
CIR	3104	**0.8468**	0.8147	0.8103	*98.46*	0.8245	*96.78*	0.8371	*98.39*
CRC1	6920	0.6882	0.6651	0.6470	*96.85*	0.7022	*98.55*	**0.7112**	*99.28*
CRC2	837	**0.8247**	0.7960	0.7606	*92.79*	0.7575	*88.05*	0.7504	*94.03*
CD1	3547	0.8073	**0.8253**	0.7968	*98.02*	0.8069	*97.18*	0.7865	*98.59*
CD2	3547	0.8297	0.8229	0.8215	*97.87*	0.8459	*97.18*	**0.8525**	*98.59*
HIV	14425	0.7165	0.8257	**0.8336**	*99.00*	0.7270	*99.31*	0.7364	*99.65*
IBD1	2742	0.7818	0.8314	**0.8652**	*97.95*	0.8225	*96.35*	0.8180	*98.18*
IBD2	1496	0.6893	0.6683	0.6842	*95.25*	**0.6979**	*93.32*	0.6961	*96.66*
MHE	3104	**0.8116**	0.7835	0.7191	*98.36*	0.7651	*96.78*	0.7183	*98.39*
OB	6386	0.8686	**0.9123**	0.8650	*98.02*	0.8791	*98.43*	0.8529	*99.22*
PAR1	10232	0.6898	0.6806	0.6787	*99.02*	**0.7293**	*99.02*	0.7130	*99.51*
PAR2	6844	0.7476	**0.7629**	0.7480	*97.24*	0.7219	*98.54*	0.7213	*99.27*
PAR3	12198	0.7770	**0.7844**	0.7596	*97.57*	0.7804	*99.18*	0.7724	*99.59*
ART	10733	0.6048	**0.6439**	0.6077	*99.30*	0.6115	*99.07*	0.6000	*99.53*

**Table 8:** Summary of feature selection methods used in the study

Method	Description
Mutual Information	Measures the dependence between each feature and the target variable to select the most informative ones.
mRMR	Selects features greedily balancing high relevance to the target and low redundancy among themselves.
LASSO	Features selected by a L1 regularized regression model that enforces sparsity, shrinking some coefficients to zero.
ReliefF	Assigns feature weights based on how well they differentiate between neighboring instances of different classes.
Random forest importances	Ranks features based on their contribution to reducing impurity in decision trees within the ensemble.
Shallow autoencoder	Learns a compressed representation of input data through unsupervised encoding and decoding.
Variational autoencoder	Probabilistic autoencoder that learns a compressed latent space representation by modeling input data as a distribution.

### Generalizing across cohorts

One of the key challenges in microbiome data analysis is to obtain feature sets that capture generalizable patterns, ensuring that the selected features are robust and applicable across different datasets rather than being influenced by specific dataset characteristics. For this reason, we conducted the cross-cohort study using our pipelines with the Parkinson’ disease data. It is important to note that this analysis was performed as a proof of concept. While the observed results are promising, the ability to consistently achieve such levels of generalization remains as an open question. To the best of our knowledge, although cross-cohort generalization is a desirable goal, it is rarely reported in 16S microbiome studies.

Our results show that the features obtained in this case study can reach a good level of generalization. The improved generalization of mRMR-selected features suggests that its greedy procedure to remove redundancy also can help to mitigate dataset-specific biases, allowing generalizable discoveries.

Another key observation is that models trained on PAR3 and tested on PAR2 achieved better generalization than the reverse. This is a reasonable outcome given the larger sample size in PAR3, which provides a broader representation of microbial variability at the training step. Larger datasets tend to capture more robust patterns, making them able to train models with lower generalization error.

## Potential Implications

The findings of this study offer new lines of research that could expand the scope and applicability of microbiome disease classification pipelines. The following potential implications highlight broader research opportunities:

- **Hybrid Approaches:** The different feature selection methods explored could be complemented by hybrid strategies, such as employing efficient greedy methods for initial filtering, followed by computationally intensive techniques like wrappers or evolutionary algorithms to refine the microbial signatures. Such combinations may obtain very reduced yet informative feature subsets.
- **Evaluation Across Harmonized Datasets:** The harmonization of datasets provides an opportunity for comprehensive meta-analyses. Evaluating feature selection methods across integrated datasets can help generalize findings, ensuring they are robust to variations in data collection, preprocessing, and experimental conditions. Validation of results across external studies would further establish the reliability of identified biomarkers.
- **Advancing Explainability for Clinical Integration:** Developing explainability interfaces and metrics tailored to microbiome data can help to close the gap between bioinformatic discoveries and practical clinical applications. By enabling understanding of the reasons behind feature selection and classification decisions, these tools would promote confidence in utilizing biomarkers for aiding diagnostics.
- **Towards Standardization and Collaboration:** As microbiome research continues to expand, standardization of feature selection practices, facilitated by reproducible workflows, will be essential. Collaborative platforms that integrate harmonized data, past results, and interpretability frameworks could accelerate discoveries in microbiome research.

## Conclusion

Taken together, our study provides some recommendations on how to improve biomarker discovery through 16S microbiome disease classification without sacrificing performance. Random forest showed strong behavior for raw relative abundances and turned out to be good baselines for pure performance. Additionally, PA normalization yielded equivalent performance to that of abundance-based transformations. On the other hand, CLR normalization combined with logistic regression helps to cope with compositionality at baseline classification and improves feature selection. Among feature selection methods, mRMR100 consistently enhanced predictive power while maintaining interpretability and compactness. While mRMR50 also ranked better, LASSO must also be considered because of its similar performance and quicker computation.

Future studies are advised to match normalization and feature selection approaches to their chosen classifiers. They should also establish a trade-off between feature set sizes and predictive accuracy, in order to maximize the robustness and relevance of the identified microbial features.

## Methods

### 16S rRNA data

The most usual sequencing technique to analyze human microbiome is 16S ribosomal RNA amplicon analysis [44]. The 16S gene is used for taxonomic and phylogenetic studies as it is universally present between different species of bacteria and archaea. It is approximately 1,600 base pairs long and contains 9 hypervariable regions (V1–V9) with a different conservation degree that can provide signatures of different bacterial species [45]. The conserved regions flanking the hypervariable region of interest can be used for PCR amplification of targets and posterior sequencing via the Illumina framework [46] or previously via the discontinued 454 pyrosequencing [47].

The resulting sequences are then clustered into operational taxonomic units (OTUs) [48] or resolved into amplicon sequence variants [49]. Finally, they are compared against a reference database in order to identify their likely taxonomy [50]. At this point, raw data have transformed into an abundance table with the number of reads of each sequence for each sample, suited for data analysis and machine learning.

### Preprocessing: filtering and normalization

As data selected were already assembled into OTU/ASV count tables, only simple filtering was needed. Datasets with an acceptable number of features were not filtered to avoid removing low-count but possibly powerful predictive OTUs. On the other hand, extremely sparse datasets, with more than 15,000 features, were filtered (Supplementary Table S1 provides original feature dimensionality and details for the datasets); that is, as in [34], we removed taxa with fewer than 10 reads across the dataset and features that were not present in more than the 1% of the dataset. Final dimensions of datasets are presented in Table 1. Relative abundances of taxa were then computed; this step is commonly referred to as a closure operation. These proportions naturally reside in the simplex, a constrained D-dimensional space defined as:


\begin{eqnarray*}
\boldsymbol{x}_{\mathrm{ra}} = \left[\frac{x_1}{\sum _{i=1}^D x_i}, \frac{x_2}{\sum _{i=1}^D x_i}, \ldots , \frac{x_D}{\sum _{i=1}^D x_i}\right] \in \mathcal {S}^{D-1}
\end{eqnarray*}

\begin{eqnarray*}
\mathcal {S}^{D-1} = \left\lbrace \boldsymbol{x} \in \mathbb {R}^D \mid x_i \ge 0, \sum _{i=1}^D x_i = 1\right\rbrace
\end{eqnarray*}

Centered log-ratio normalization was applied to address the compositional nature of microbiome data, and its performance was compared with RA and other transformations, including logRA and PA encoding.

Aitchison’s CLR normalization [51] transforms compositional data from the simplex into the Euclidean space by taking the logarithm of each feature’s relative abundance, divided by the geometric mean $G(x)$ of all features within a sample.


\begin{eqnarray*}
\boldsymbol{x}_{clr} = \left[\log \left(\frac{x_{\mathrm{ra}1}}{G(\boldsymbol{x}_{\mathrm{ra}})}\right), \log \left(\frac{x_{\mathrm{ra}2}}{G(\boldsymbol{x}_{\mathrm{ra}})}\right), \ldots , \log \left(\frac{x_{\mathrm{ra}D}}{G(\boldsymbol{x}_{\mathrm{ra}})}\right)\right]
\end{eqnarray*}

However, due to the presence of zero counts in microbiome datasets (common when taxa are not detected in a given sample), it is necessary to introduce a pseudocount to avoid undefined logarithmic calculations. The choice of pseudocount remains a critical yet unresolved issue. Different pseudocount values can impact the results, as they affect the ratios between features and distribution of the transformed data. While commonly used values such as 0.5, 1, or small constants close to zero are practical solutions, there is no consensus on an optimal choice. We applied a pseudocount $P=0.5$ to all datasets, as done in the limma-voom approach [52], to allow for the CLR and logRA normalizations.

Finally, the PA transformation is a binary representation where each feature is set to 1 if its relative abundance is greater than 0, and 0 otherwise.


\begin{eqnarray*}
x_{\mathrm{pa}_i} = \left\lbrace \begin{array}{@{}l@{\quad }l@{}}1 & \mathrm{if}\ \ x_{\mathrm{ra}_i} > 0 \\
0 & \mathrm{otherwise} \end{array}\right.
\end{eqnarray*}

Rarefaction is another commonly used preprocessing step in microbiome studies, performed directly on count data, before the previously defined normalizations. It involves randomly subsampling each sample read (without replacement) to the same sequencing depth to mitigate biases introduced by varying library sizes [53]. While it can help control for differences in sampling depth, rarefaction discards data and introduces stochasticity, which can reduce statistical power.

### Feature selection techniques overview

Feature selection is a critical step in high-dimensional data analysis, as it reduces the number of variables while retaining the most informative ones. This enhances interpretability and may improve model performance. By selecting a small number of features, such as microbial genera or species, their biological relationships with the target diseases can be more easily identified. Moreover, removing redundant and irrelevant features can lead to an improvement of the generalization ability of the models.

In this study, we primarily focused on the filter approach, which evaluates features independently of any classification algorithm. This provides a model-agnostic ranking that can be applied across different classifiers while minimizing the risk of overfitting [54]. Additionally, we employed autoencoders to explore dimensionality reduction techniques. While not strictly a feature selection method, autoencoders identify compressed representations of the data without the use of class information. This deep learning approach is emerging as a growing-interest technique in the microbiome domain [55]. As a reference, we also included LASSO, a widely used technique in the few studies addressing this problem, to benchmark the performance of our approaches.

#### Mutual information and mRMR

MI is a fundamental concept in information theory that measures the mutual dependency between 2 variables [56]. It estimates how much knowledge about one variable is obtained by observing the other. One of the main advantages of MI lies in its ability to capture nonlinear relationships, unlike simpler correlation metrics that only account for linear ones. This flexibility is particularly useful in biological data, where complex interactions are common. However, MI-based feature selection provides a ranking of features that has not accounted for redundancy, leading to the selection of features that may be highly correlated and thus less informative collectively. We applied the implementation available at scikit-learn [31].

The mRMR algorithm was initially designed for gene selection in transcriptomics. It aims to strike a balance between selecting features that are maximally relevant to the target variable and minimally redundant with each other [57]. Unlike simpler MI-based methods [58], mRMR imposes additional constraints to avoid selecting correlated features, which is particularly useful in high-dimensional datasets. In particular, we considered 2 subset sizes to retain for comparisons (50 and 100); in this way, we can also evaluate the redundancy present in the data. We applied the open-source implementation available in [59].

This algorithm has demonstrated its effectiveness in identifying small, biologically meaningful signatures in transcriptomics, especially in cancer studies. Applications of mRMR to 16S rRNA microbiome data have yielded mixed results, often selecting relatively large feature sets [12].

#### ReliefF

ReliefF is a feature selection algorithm that extends the original Relief method, improving its capabilities to handle unbalanced and noisy datasets [60]. This algorithm evaluates features by estimating their ability to distinguish between instances that are near each other (nearest hits and misses), leveraging a nearest-neighbor approach to assess the relevance of features. Unlike most filter methods, ReliefF considers interactions among features, making it particularly suitable for high-dimensional datasets where complex relationships may exist. We applied the ReBATE open-source implementation [61].

#### Autoencoders

**Figure 11:**
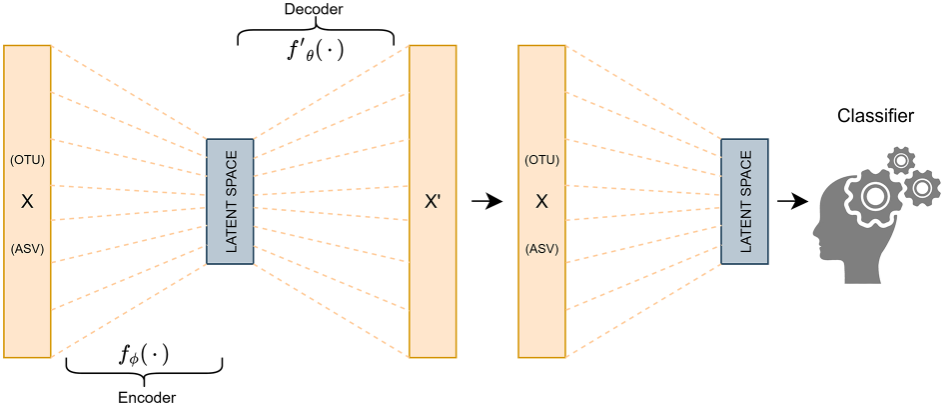
Shallow autoencoder feature reductor diagram. Latent space is calculated via optimization of the reconstruction loss $L(x,x{'})=||x-x{'}|{|}^{2}=||x-f{{'}}_{\theta }({f}_{\phi }(x))|{|}^{2}$.

Autoencoders are a specialized class of artificial neural networks that can learn efficient compressed data representations without the information from the labels. Its architecture consists of 2 main parts: an encoder layer that aims to transform the data into a reduced representation, the latent space. From the output of this layer, a decoder tries to rebuild the original input. During training, the loss function measures the difference between input and output layers; by minimizing this reconstruction loss, the latent space captures the most important features of the input data (see Fig. 11 ). While autoencoders are not a traditional feature selection method, they have been pointed out as an interesting hypothesis for task-adapted dimensionality reduction that can cope with sparse matrices and a low number of samples [55, 62]. For our study, we used DeepMicro [63], a deep learning framework designed to allow an effective representation of microbiome profiles, to learn the latent space representation as a previous step for the classification task. In particular, we used the shallow autoencoders that have only 1 hidden layer, as they showed the most promising results in the DeepMicro case studies and also because of the small sample sizes of some of the considered datasets. We chose 2 different latent space sizes (50 and 100), allowing a comparison with the other algorithms, and a variational autoencoder (50). DeepMicro also offers the later classification step, but to adapt autoencoders to our validation strategy, we preferred to use only the latent representation learning step.

#### LASSO

LASSO is a regression-based method widely used for feature selection. By effectively shrinking the coefficients of less relevant features to zero, it stands as a common FS method for high-dimensional datasets. In microbiome studies, LASSO is frequently employed either as a standalone feature selection method or as part of a pipeline paired with logistic regression [32, 64]. In our case, the logistic regression classifier uses L2-regularization; therefore, when combined in a pipeline, both techniques will be hybridized.

#### Random forest feature importance

Random forest is an ensemble learning method that can also be used for feature selection by evaluating the importance of each feature in the model’s construction [65]. The core idea is to build a large number of decision trees, with each tree trained on a random subset of the data. The importance of a feature is calculated by measuring how much it contributes to reducing impurity across all the trees. When a particular feature is repeatedly selected at the top of the trees for splitting the nodes, it receives a higher importance score. This provides a feature relevance metric that accounts for interactions between features by construction.

### Classification models, tuning, and validation

Our study leveraged both ensemble and traditional classification algorithms to evaluate the impact of feature selection and normalization techniques. Ensemble methods have been demonstrated as very powerful classifiers, among which RF and XGBoost are the 2 most popular ones. In addition to them, we employed traditional classifiers, including SVM, LR, and KNN.

**Figure 12:**
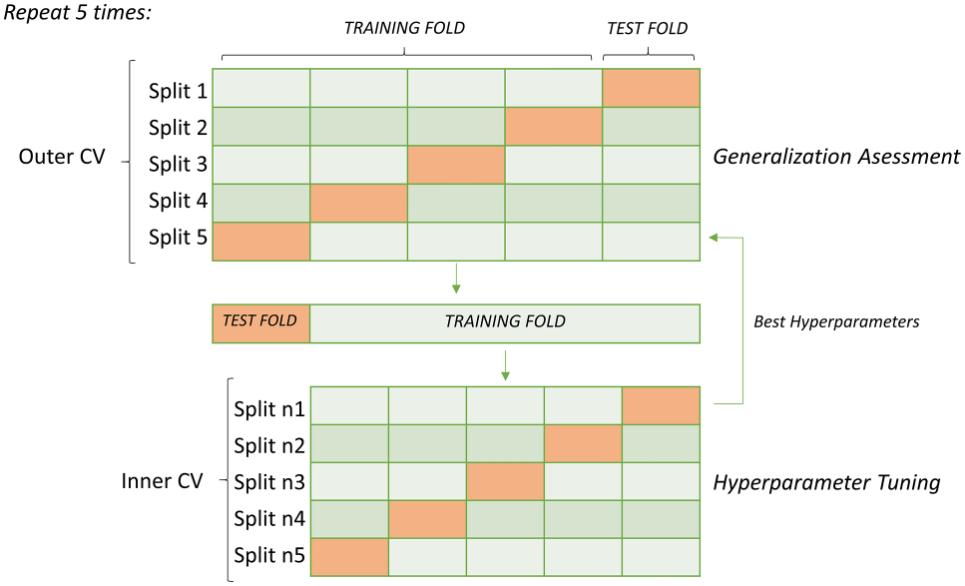
Repeated nested cross-validation procedure.

In order to get a robust measure of generalization performance, each model was validated through 5 times repeated nested 5-fold cross-validation (see Fig. 12). To ensure fair comparisons, all classifiers underwent hyperparameter optimization using a grid search in the inner validation loop. This computationally intensive process was selected to ensure robust performance estimates and minimize possible bias and potential overfitting from the model parameters selection [66].

The AUC was chosen as the objective metric, as it is the most commonly used performance measure in microbiome classification studies. AUC offers a general evaluation of model performance across varying decision thresholds, being less sensitive to imbalance in datasets, where accuracy may fail to capture the true predictive power of a classifier.

#### Logistic regression

Logistic regression is a linear model that is commonly used for binary classification tasks. It estimates the probability of an instance belonging to a particular class by fitting the input features to a logistic function. In our analysis, we incorporated L2 regularization (Ridge) to improve model performance and prevent overfitting. This technique adds a penalty term proportional to the square of the coefficient values to the loss function [67].

#### Support vector machine

Support vector machines classify data by finding the optimal hyperplane that maximally separates classes in a high-dimensional feature space. SVMs can handle nonlinear boundaries using kernel functions (radial basis or polynomial) and are effective in high-dimensional settings [68]. In our case, we selected a radial-basis kernel, as the results of [69] stated its universality, meaning it can approximate any continuous function arbitrarily well given sufficient data. This property implies that the RBF kernel can generalize other kernels under appropriate conditions.

#### Random forest

Random forest is an ensemble learning method that constructs multiple decision trees on different sets of features and samples (bagging) during training and outputs the mode of their predictions. By aggregating predictions from numerous trees and data samples, RF reduces overfitting and improves robustness. [65]

#### Boosting

Boosting is another ensemble learning approach that builds a strong classifier by sequentially combining a set of weak models. Trees are trained iteratively, with each new model focusing on correcting the instances that were misclassified by the previous models. For our analysis, we employed XGBoost, a powerful and widely adopted implementation of gradient boosting known for its high performance and scalability. [70]

#### K-nearest neighbor

KNN is a nonparametric, lazy-learning method that classifies samples by majority vote provided by the classes of their $k$ closest neighbors in the feature space. It requires no explicit training but scales poorly with large datasets due to its reliance on distance computations (e.g., Euclidean or Manhattan). Performance depends strongly on the choice of $k$, which, in conjunction with the distance metric, needs to be tuned properly [71].

### Statistical analysis

Comparing results of multiple algorithms across multiple datasets requires the use of statistical tests to avoid reaching conclusions due to random chance [72].

To analyze pairwise differences between pairs of classifiers or normalization techniques, we employed the Wilcoxon signed-rank test. This paired nonparametric test was chosen due to its suitability for scenarios where the assumptions of normality of parametric tests may not hold.

We also employed the Friedman test, followed by the Finner post hoc procedure, to analyze the differences between multiple classifiers and feature selection methods. This approach was chosen by evidence of superior power compared to other procedures [73], which means it has a lower probability of making a type II error. The Friedman test allowed us to determine the presence of significant differences in classifier performance for each normalization, while the Finner post hoc procedure enabled us to identify which methods differed from each other in a controlled manner.

## Availability of Source Code and Requirements

- Project name: 16SMicrobiomeMLFS
- Project homepage: https://github.com/nach00gar/16SMicrobiomeMLFS
- Operating system(s): Platform independent
- Programming language: Python, R
- Other requirements: sklearn 1.5.2, composition_stats 2.0.0, xgboost 2.1.2, keras 2.2.4, mrmr-selection 0.2.8, skrebate 0.62, DeepMicro [63], stac (Statistical Tests for Algorithms Comparison [74])
- License: GNU GPL
- SciCrunch RRID: SCR_027170
- Bio.tools ID: 16smicrobiomemlfs
- WorkflowHub ID: 1807 [75]

## Additional Files

**Supplementary Fig. S1**. Rarefaction effect on baseline classification for each normalization.

**Supplementary Fig. S2**. Finner’s test result comparing classifier ranks for CLR feature selection pipelines. Friedman’s test results were significant with $p_{val} = 3.5 \cdot 10^{-47}$.

**Supplementary Fig. S3**. Finner’s test result comparing feature selection ranks across all normalization + feature selection pipelines.

**Supplementary Fig. S4**. Mean execution time by fold at the outer cross-validation by feature selection algorithm and dataset.

**Supplementary Table S1**. Dataset description and OTU filtering results. IR stands for the imbalance ratio, and the numbers in parentheses indicate the distribution of samples across classes.

**Supplementary Table S2**. Rarefaction comparisons for each normalization. Wilcoxon test *P* values.

**Supplementary Table S3**. RA baseline results.

**Supplementary Table S4**. CLR baseline results.

**Supplementary Table S5**. logRA baseline results.

**Supplementary Table S6**. PA baseline results.

**Supplementary Table S7**. LASSO logistic regression results with CLR normalization, comparing performance with different numbers of features selected.

giaf096_Supplemental_File_Revised

giaf096_Authors_Response_To_Reviewer_Comments_Original_Submission

giaf096_GIGA-D-25-00079_Original_Submission

giaf096_GIGA-D-25-00079_Revision_1

giaf096_Reviewer_1_Report_Original_Submission Justin Morris -- 4/17/2025

giaf096_Reviewer_2_Report_Original_Submission Zuzanna Karwowska -- 4/5/2025

## Abbreviations

AE: autoencoder; AUC: area under the receiver operating characteristic curve; ASV: amplicon sequence variant; CLR: centered log-ratio; FS: feature selection; IR: imbalance ratio; KNN: k-nearest neighbors; LASSO: least absolute shrinkage and selection operator; LR: logistic regression; MIFS: Mutual Information feature selection; ML: machine learning; mRMR: minimum redundancy maximum relevancy; OTU: operational taxonomic unit; PA: presence–absence; RA: relative abundances; RF: random forest; rRNA: ribosomal RNA; SVM: support vector machine; VAE: variational autoencoder.

## Disclosure of Use of AI-Assisted Tools

Large language models [76] were used to improve the flow of certain paragraphs and to find grammatical errors. The use of the LLM does not negatively affect the empirical data and conclusions. The authors have thoroughly reviewed the text and are fully responsible for its content.

## Data Availability

Supporting data used for the analyses can be accessed by our code repository. Data were downloaded from MicrobiomeHD [15], MLRepo [16], and [17] article repository.
