# Correction to: Exploring the role of normalization and feature selection in microbiome disease classification pipelines

**DOI:** 10.1093/gigascience/giag050

**Published:** 2026-05-06

This is a correction to: Ignacio Garach Vélez, Francisco Manuel Ortuño Guzmán, Ignacio Rojas Ruiz, Luis Javier Herrera Maldonado, Exploring the role of normalization and feature selection in microbiome disease classification pipelines, *GigaScience*, Volume 14, 2025, giaf096, https://doi.org/10.1093/gigascience/giaf096

In the originally published article, the information listed for the second funding project was incorrect. The funding section originally read:

“This work is supported by grant PID2021-128317OB-I00 (Principal Investigators: Ignacio Rojas and Luis Javier Herrera) and PCI2023-146016-2 (Principal Investigators: Francisco Manuel Ortuño and Olga Valenzuela), funded by MICIU/AEI/10.13039/501100011033 and co-funded by the European Union. Funding for open access charge: Universidad de Granada/CBUA”.

This has been emended to:

“This work is supported by grant PID2021-128317OB-I00 (Principal Investigators: Ignacio Rojas and Luis Javier Herrera) funded by MICIU/AEI/10.13039/501100011033 and co-funded by the European Union and C-ING-172-UGR23, funded by the Department of University, Research and Innovation of the Andalusian Regional Government (Junta de Andalucía) (Principal Investigators: Francisco Manuel Ortuño and Olga Valenzuela). Funding for open access charge: Universidad de Granada/CBUA.

