# Supplementary material for: Exploring the role of normalization and feature selection in microbiome disease classification pipelines
Source: Gigascience. 2025 Sep 2;14:giaf096. doi: 10.1093/gigascience/giaf096 (PMC12402773; doi:10.1093/gigascience/giaf096)
Supplement: giaf096_Supplemental_File_Revised [file giaf096_supplemental_file_revised.pdf]

Supplementary Material

July 13, 2025

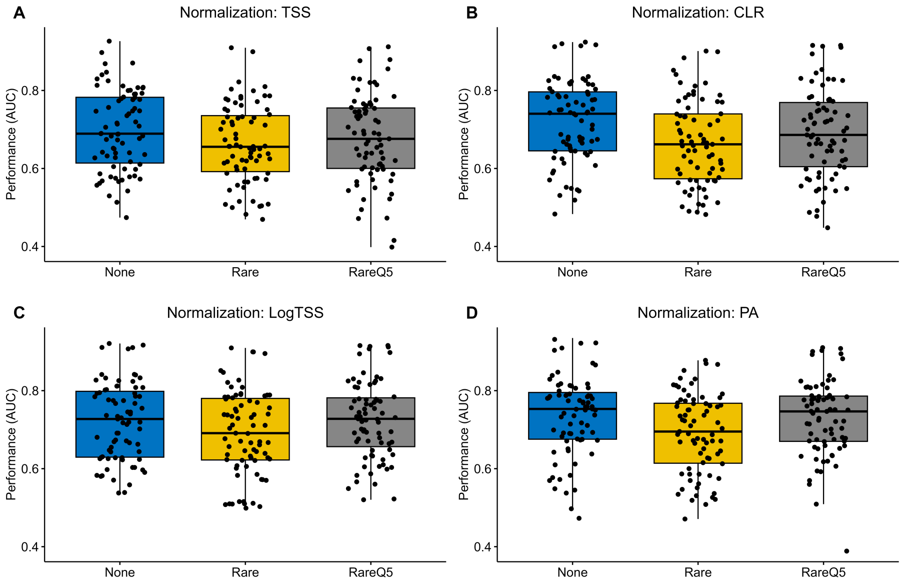

Supplementary Figure S1: Rarefaction effect on baseline classification for each normalization.

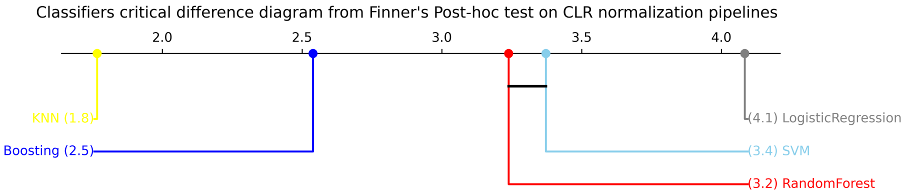

Supplementary Figure S2: Finner's test result comparing classifier ranks for CLR feature selection pipelines. Friedman's test results were significative with $p_{val} = 3.5 \cdot 10^{-47}$

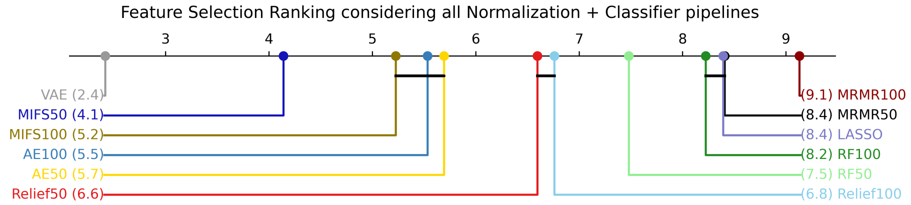

Supplementary Figure S3: Finner's test result comparing feature selection ranks across all normalization + feature selection pipelines.

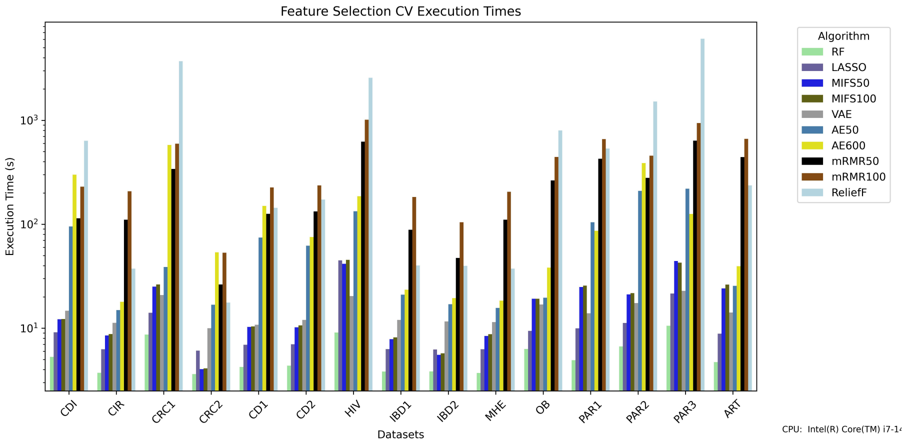

Supplementary Figure S4:Mean execution time by fold at the outer Cross Validation by Feature Selection algorithm and dataset

Dataset	Samples	Original Features	Filtered	IR	Region
ART	114 (86, 28)	10733	10733	3.07	V1-V2
CDI	336 (93, 243)	19314	3456	2.61	V3-V5
CRC1	490 (229, 261)	6920	6920	1.14	V4
CRC2	102 (46, 56)	837	837	1.22	V3
HIV	350 (293, 57)	104134	14425	5.14	V3-V4
CD1	140 (78, 62)	3547	3547	1.26	V4
CD2	160 (68, 92)	3547	3547	1.35	V4
IBD1	91 (67, 24)	36349	2742	2.79	V3-V5
IBD2	114 (68, 46)	15496	1496	1.48	V5-V6
CIR	77 (51, 26)	3104	3104	1.96	V2
MHE	77 (26, 51)	3104	3104	1.96	V2
OB	281 (220, 61)	56790	6386	3.61	V2
PAR1	148 (74, 74)	10232	10232	1.00	V1-V3
PAR2 *	333 (201, 132)	6844	6844	1.52	V4
PAR3 *	507 (323, 184)	12198	12198	1.76	V4

Supplementary Table T1: Datasets description and OTU filtering results. IR stands for the imbalance ratio, and the numbers in parentheses indicate the distribution of samples across classes.

Normalization	Rarefaction	RarefactionQ5	Rarefaction vs RarefactionQ5
RA	$7.1 \cdot 10^{-5}$	$1.4 \cdot 10^{-4}$	0.33
CLR	$9.4 \cdot 10^{-7}$	$4.2 \cdot 10^{-4}$	$4.1 \cdot 10^{-3}$
logRA	0.041	0.79	$2.3 \cdot 10^{-4}$
PA	$9.7 \cdot 10^{-5}$	0.26	$8.2 \cdot 10^{-6}$

Supplementary Table T2: Rarefaction comparisons for each normalization. Wilcoxon test p-values

Dataset	Boosting	KNN	Logistic Regression	Random Forest	SVM
CDI	0.9264	0.6106	0.8082	0.8973	0.7998
CIR	0.7906	0.6322	0.5728	0.8468	0.5619
CRC1	0.6279	0.5570	0.6403	0.6882	0.6504
CRC2	0.7086	0.6831	0.7539	0.8247	0.7565
CD1	0.7928	0.6836	0.7468	0.8073	0.7194
CD2	0.8132	0.6767	0.7735	0.8297	0.7138
HIV	0.7791	0.5786	0.7765	0.7165	0.7910
IBD1	0.7478	0.7354	0.7057	0.7818	0.5978
IBD2	0.6648	0.6357	0.5785	0.6893	0.5809
MHE	0.6879	0.5423	0.6743	0.8116	0.5134
OB	0.8064	0.8005	0.8403	0.8686	0.8704
PAR1	0.6731	0.5703	0.5750	0.6898	0.4742
PAR2	0.7402	0.5988	0.6396	0.7476	0.6173
PAR3	0.7832	0.5297	0.6519	0.7770	0.6272
ART	0.5864	0.5681	0.6417	0.6048	0.5663

Supplementary Table T3: RA Baseline Results

Dataset	Boosting	KNN	Logistic Regression	Random Forest	SVM
CDI	0.9199	0.7523	0.9171	0.8358	0.9145
CIR	0.7425	0.5308	0.8147	0.7501	0.7969
CRC1	0.6748	0.5705	0.6651	0.6457	0.6620
CRC3	0.6698	0.6135	0.7960	0.6873	0.7507
CD1	0.7768	0.6077	0.8253	0.6430	0.8163
CD2	0.8050	0.7005	0.8229	0.7426	0.8166
HIV	0.7404	0.6766	0.8257	0.6582	0.7903
IBD1	0.7755	0.6806	0.8314	0.7447	0.8357
IBD2	0.6447	0.6746	0.6683	0.5937	0.6343
MHE	0.7432	0.6715	0.7835	0.7373	0.8189
OB	0.8033	0.8304	0.9123	0.7879	0.9240
PAR1	0.6522	0.5189	0.6806	0.6271	0.5423
PAR2	0.7224	0.5454	0.7629	0.6394	0.7626
PAR3	0.7697	0.6247	0.7844	0.7114	0.7824
ART	0.5883	0.4832	0.6439	0.5518	0.5489

Supplementary Table T4: CLR Baseline Results

Dataset	Boosting	KNN	Logistic Regression	Random Forest	SVM
CDI	0.9209	0.7195	0.9168	0.8415	0.9072
CIR	0.8417	0.5977	0.8328	0.8027	0.7949
CRC1	0.6279	0.5828	0.6665	0.6910	0.6691
CRC2	0.6437	0.6549	0.7934	0.6257	0.7764
CD1	0.7549	0.5967	0.8271	0.6627	0.8080
CD2	0.7933	0.6237	0.8256	0.6804	0.8119
HIV	0.7303	0.5586	0.8237	0.6912	0.7857
IBD1	0.7852	0.7134	0.8324	0.7673	0.8402
IBD2	0.6227	0.5905	0.6431	0.5803	0.6031
MHE	0.7273	0.7454	0.7365	0.7176	0.8116
OB	0.8081	0.7403	0.9108	0.8017	0.9104
PAR1	0.7340	0.6281	0.6734	0.6286	0.6712
PAR2	0.7193	0.5389	0.7672	0.6031	0.7451
PAR3	0.7745	0.6474	0.7747	0.7019	0.7844
ART	0.6256	0.5819	0.6304	0.5707	0.5376

Supplementary Table T5: logRA Baseline Results

Dataset	Boosting	KNN	Logistic Regression	Random Forest	SVM
CDI	0.9092	0.6929	0.9224	0.9035	0.9216
CIR	0.7593	0.5723	0.8705	0.7992	0.8389
CRC1	0.6510	0.5696	0.6750	0.6896	0.6711
CRC2	0.6376	0.7421	0.7845	0.8178	0.7326
CD1	0.7375	0.7504	0.7817	0.7864	0.7869
CD2	0.7853	0.7590	0.8120	0.8292	0.8132
HIV	0.7356	0.6938	0.7587	0.6903	0.7692
IBD1	0.7797	0.7749	0.8139	0.8174	0.8072
IBD2	0.6119	0.6727	0.6916	0.6991	0.7109
MHE	0.7144	0.6735	0.7883	0.8062	0.7915
OB	0.8659	0.7709	0.9314	0.8474	0.9350
PAR1	0.5829	0.5451	0.6458	0.6423	0.4728
PAR2	0.7175	0.6761	0.7740	0.7503	0.7684
PAR3	0.7524	0.6824	0.7877	0.7531	0.7804
ART	0.5772	0.5485	0.6099	0.5375	0.4972

Supplementary Table T6: PA Baseline Results

Dataset	Lasso	Lasso50	Lasso100
CRC1	0.648	0.665	0.646
CRC2	0.760	0.748	0.760
CD1	0.792	0.791	0.792
CD2	0.822	0.827	0.822
PAR	0.676	0.679	0.675
PAR2	0.748	0.709	0.740
PAR3	0.759	0.723	0.748
HIV	0.836	0.798	0.822
OB2	0.867	0.848	0.866
CDI	0.915	0.907	0.913
CIRR	0.814	0.813	0.814
IBD1	0.870	0.852	0.870
IBD2	0.685	0.687	0.685
MHE	0.723	0.716	0.723
ART	0.608	0.585	0.608
**Mean**	**0.768**	**0.746**	**0.764**

Supplementary Table T7: Lasso logistic regression results with CLR normalization, comparing performance with different numbers of features selected.
