# Supplementary material for: Exploring the role of normalization and feature selection in microbiome disease classification pipelines
Source: Gigascience. 2025 Sep 2;14:giaf096. doi: 10.1093/gigascience/giaf096 (PMC12402773; doi:10.1093/gigascience/giaf096)
Supplement: giaf096_GIGA-D-25-00079_Original_Submission [file giaf096_giga-d-25-00079_original_submission.pdf]

GigaScience

Exploring the role of normalization and feature selection in microbiome disease classification pipelines

--Manuscript Draft--

**Manuscript Number:**	GIGA-D-25-00079
**Full Title:**	Exploring the role of normalization and feature selection in microbiome disease classification pipelines
**Article Type:**	Research
**Funding Information:**	Ministerio de Ciencia e Innovación (PID2021-128317OB-I00) — Prof. Ignacio Rojas Ruiz<br>Consejería de Conocimiento, Investigación y Universidad, Junta de Andalucía (PCI2023- 146016-2) — Dr. Francisco Manuel Ortuño Guzmán
**Abstract:**	Background: Disease classification using 16S rRNA microbiome data faces challenges of high dimensionality, compositionality, and sparsity, compounded by the inherent small sample sizes in many studies. Machine learning and feature selection techniques offer potential to identify robust biomarkers and improve classification performance; however, their comparative effectiveness across diverse methods and datasets has been insufficiently explored. This study evaluates multiple feature selection techniques alongside normalization strategies, focusing on their interplay with classifier performance. Results: Our analyses revealed that Centered Log-Ratio normalization improves the performance of logistic regression and support vector machine models and facilitates feature selection, whereas random forests yielded strong results using relative abundances. Among feature selection methods, minimum redundancy maximum relevancy (mRMR) surpassed most methods in identifying compact and biologically meaningful biomarker sets and demonstrated performance comparable to LASSO, which obtained top results requiring lower computation times. Autoencoders struggled to achieve small biomarker sets and lacked interpretability, and ReliefF struggled with data sparsity. Conclusions: Overall, feature selection pipelines improved model focus and robustness via a massive reduction of the feature space. mRMR and LASSO emerged as the most effective methods across datasets.
**Corresponding Author:**	Ignacio Garach Vélez, MSc<br>Universidad de Granada Escuela Tecnica Superior de Ingenierias Informatica y de Telecomunicacion<br>GRANADA, GRANADA SPAIN
**Corresponding Author Secondary Information:**	
**Corresponding Author's Institution:**	Universidad de Granada Escuela Tecnica Superior de Ingenierias Informatica y de Telecomunicacion
**Corresponding Author's Secondary Institution:**	
**First Author:**	Ignacio Garach Vélez, MSc
**First Author Secondary Information:**	
**Order of Authors:**	Ignacio Garach Vélez, MSc<br>Francisco Manuel Ortuño Guzmán<br>Ignacio Rojas Ruiz<br>Luis Javier Herrera Maldonado
**Order of Authors Secondary Information:**	
**Additional Information:**	
**Question**	**Response**

Powered by Editorial Manager® and ProduXion Manager® from Aries Systems Corporation

Are you submitting this manuscript to a special series or article collection?	No
**Experimental design and statistics**<br><br>Full details of the experimental design and statistical methods used should be given in the Methods section, as detailed in our Minimum Standards Reporting Checklist. Information essential to interpreting the data presented should be made available in the figure legends.<br><br>Have you included all the information requested in your manuscript?	Yes
**Resources**<br><br>A description of all resources used, including antibodies, cell lines, animals and software tools, with enough information to allow them to be uniquely identified, should be included in the Methods section. Authors are strongly encouraged to cite Research Resource Identifiers (RRIDs) for antibodies, model organisms and tools, where possible.<br><br>Have you included the information requested as detailed in our Minimum Standards Reporting Checklist?	Yes
**Availability of data and materials**<br><br>All datasets and code on which the conclusions of the paper rely must be either included in your submission or deposited in publicly available repositories (where available and ethically appropriate), referencing such data using a unique identifier in the references and in the "Availability of Data and Materials" section of your manuscript.<br><br>Have you have met the above requirement as detailed in our Minimum	Yes

Standards Reporting Checklist?	
GigaScience has policies and guidelines in place for the use of generative AI-writing tools such as ChatGPT. If you have used such writing tools to assist with writing the manuscript this must be declared and cited in the text. Authors should not list AI-writing tools and other AI-assisted technologies as an author or co-author and should acknowledge that they are fully responsible for text generated or refined by AI-writing tools.<p><br><br>A summary of use (particularly in the introduction or among methods) needs to be included at the end of the paper, and the outputs should also be included as a supplementary file hosted in GigaDB or other open repositories. Please read our guidelines for more information. <p><br><br>By submitting to GigaScience, you are aware of the journal's AI-writing tools policy, and if you have declared use of such tools below, you have acknowledged this where appropriate in your manuscript and have made a summary of use and outputs available. <p><br><b>AI-assisted writing tools have been used in the preparation of this manuscript?	Yes

Manuscript

Click here to access/download;Manuscript;Article FS Microbiome.pdf

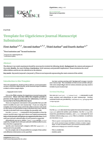

GigaScience, 2025, 1–14

doi: xx.xxxx/xxxx
Manuscript in Preparation
RESEARCH

RESEARCH

Exploring the role of normalization and feature selection in microbiome disease classification pipelines

Ignacio Garach Vélez[1*], Francisco Manuel Ortuño Guzmán[1], Ignacio Rojas Ruiz[1] and Luis Javier Herrera Maldonado[1]

[1]Computer Engineering, Automatics and Robotics Department, University of Granada, 18071 Granada, Spain;

[*] Correspondence address: igarachv@ugr.es

Abstract

Background: Disease classification using 16S rRNA microbiome data faces challenges of high dimensionality, compositionality, and sparsity, compounded by the inherent small sample sizes in many studies. Machine learning and feature selection techniques offer potential to identify robust biomarkers and improve classification performance; however, their comparative effectiveness across diverse methods and datasets has been insufficiently explored. This study evaluates multiple feature selection techniques alongside normalization strategies, focusing on their interplay with classifier performance. **Results**: Our analyses revealed that Centered Log-Ratio normalization improves the performance of logistic regression and support vector machine models and facilitates feature selection, whereas random forests yielded strong results using relative abundances. Among feature selection methods, minimum redundancy maximum relevancy (mRMR) surpassed most methods in identifying compact and biologically meaningful biomarker sets and demonstrated performance comparable to LASSO, which obtained top results requiring lower computation times. Autoencoders struggled to achieve small biomarker sets and lacked interpretability, and ReliefF struggled with data sparsity. **Conclusions**: Overall, feature selection pipelines improved model focus and robustness via a massive reduction of the feature space. mRMR and LASSO emerged as the most effective methods across datasets.

Key words: microbiome; biomarkers; machine learning; feature selection; classification;

Background

Microbial communities are present in almost every part of the human body, from the mouth to the gut, skin, and reproductive tract. The gut microbiome, widely recognized as the most extensively studied microbial community, has attracted significant attention in recent years. Numerous studies have linked microbiota dysbiosis to the development of different health problems [1]. Not only infectious diseases, but also cancer [2], inflammatory disorders such as Crohn's disease [3] or arthritis [4], and even neurodegenerative disorders such as Parkinson due to the gut-brain axis [5].

Although numerous studies employing classical statistical methods have been conducted to demonstrate these connections, the rapid advancement of next-generation sequencing technologies and the availability of increasingly larger, higher-quality datasets with more diverse cohorts underscore the potential of the use of machine learning techniques in these studies. These new approaches offer opportunities to uncover complex patterns and associations that might be challenging to detect using traditional statistics, potentially leading to more refined insights and robust predictive models.

The task of detecting the presence of a disease in a host from the gut microbiome has been approached in various ways [1]. Machine learning methods, and more specifically the supervised learning paradigm, enable researchers to address this disease classification problem from microbiome data with optimal prediction accuracy, helping to identify key biomarkers and therapeutic targets [6].

16S rRNA microbiome data presents several inherent challenges that complicate its application in disease classification tasks. Firstly, the high dimensionality of these datasets, with hundreds or thousands of features (Operational Taxonomic Units -OTUS- or Amplicon Sequence Variants -ASVs-) representing bacterial species, often greatly exceeds the number of available samples, leading to the classic curse of dimensionality problem [7]. Additionally, the

Compiled on: February 25, 2025.
Draft manuscript prepared by the author.

sparse nature of this data [8], where most taxa have very low or absent abundances in many samples, increases the chance of overfitting and risks model generalization. Moreover, microbiome data are compositional [9], meaning the abundances of taxa are proportions that sum up to one (or any read number if not under closure operation). This fact introduces important dependencies between features that can harm Machine Learning (ML) algorithms and must be properly accounted for, in order to avoid misleading conclusions. These challenges emphasize the need for normalization and advanced techniques that respect the unique characteristics of microbiome data [10].

In this context, feature selection arises as a great tool for these challenges, allowing to find a small set of taxa biomarkers and helping classifiers to deal with the high-dimensional compositional-sparse data, ideally improving performance. In this article we aim to evaluate multiple feature selection methods and their interaction with classifiers to identify robust microbial markers across diseases using a large collection of 16 rRNA disease classification datasets. We also compare the performance of models with and without CLR normalization [11] to analyze its impact. Feature selection has been used to address this problem with some cohorts [12, 13], but a systematic comparison in multiple datasets as the one presented here is lacking. This offers a broader understanding of feature selection and model interactions in the field of microbiome data modeling.

Data Description

Although some big initiatives have been developed to centralize microbiome data studies such as The Human Microbiome Project [14] or The American Gut Project [15], big curated repositories with metadata enabling further study of disease classification problems are lacking. To compare the performance of our pipelines we leveraged 16S gut datasets from MicrobiomeHD, a standardized database of human gut 16S microbiome case-control studies and their associated patient metadata,[16] and MLrepo, a repository of curated microbiome-related supervised learning tasks, [17]. We selected datasets containing at least 75 samples, and a minimum 1:6 imbalance ratio between cases and controls. To enhance the benchmark, we retrieved 2 more gut datasets online with abundance tables and metadata available [18]. Multiple datasets from MicrobiomeHD that exhibited extremely low performance in preliminary tests (an AUC lower than 0.60) were excluded from the final analysis to avoid biases in the interpretation of the results. Similarly, datasets with excessively high performance (AUC greater than 0.95) were also excluded, as their classification tasks were too simple, offering limited variability and failing to provide meaningful differences between algorithms. The final selected datasets and their respective original studies are available at Table 1. A total of 3320 gut samples from 15 datasets were considered.

Table 1. Benchmark datasets used in our classification analysis, including their imbalance ratios (IR) and references.

Dataset	Samples	Features	IR	Reference
ART	114 (86, 28)	10 733	3.07	[19]
CDI	336 (93, 243)	3456	2.61	[20]
CRC1	490 (229, 261)	6920	1.14	[21]
CRC2	102 (46, 56)	837	1.22	[22]
HIV	350 (293, 57)	14 425	5.14	[23]
CD1	140 (78, 62)	3547	1.26	[24]
CD2	160 (68, 92)	3547	1.35	[24]
IBD1	91 (67, 24)	2742	2.79	[25]
IBD2	114 (68, 46)	1496	1.48	[26]
CIR	77 (51, 26)	3104	1.96	[27]
MHE	77 (26, 51)	3104	1.96	[27]
OB	281 (220, 61)	6386	3.61	[28]
PAR1	148 (74, 74)	10 232	1.00	[29]
PAR2	333 (201, 132)	6844	1.52	[30]
PAR3	507 (323, 184)	12 198	1.76	[31]

ART: Arthritis; CDI: Clostridium difficile Infection; CRC1 and CRC2: Colorectal Cancer; HIV: Human Immunodeficiency Virus; CD1 and CD2: Crohn's Disease; IBD1 and IBD2: Inflammatory Bowel Disease; CIR: Cirrhosis; MHE: Minimal Hepatic Encephalopathy; OB: Obesity; PAR1, PAR2, and PAR3: Parkinson's Disease.
CD1 and CD2 were taken from MLRepo, PAR2 and PAR3 were retrieved from their respective reference, and the remaining datasets were obtained from MicrobiomeHD.

Classifier	Hyperparameter	Parameters
Random Forest	n_estimators	[200, 300, 400]
	max_features	[sqrt, log2]
	max_depth	[None, 3, 5, 7, 8]
KNN	n_neighbors	[7, 9, 11, 13, 15, 17, 19, 21]
	weights	[uniform, distance]
SVM	C	[0.001, 0.1, 1, 10, 100, 1000]
	kernel	['rbf']
	gamma	[scale, auto]
Logistic Regression	C	np.logspace(-4, 4, 20)
Boosting	max_depth	[3, 5, 7, 8]
	n_estimators	[300, 500, 800]

Table 2. Hyperparameter grid for classifiers used in the study.

Analyses

Empirical evidence is presented on the performance levels of baseline machine learning (ML) models across 16S microbiome datasets, focusing on the impact of normalization techniques and feature selection methods. Validation AUC, derived from a nested cross-validation procedure, is used as the primary metric to assess model performance. The models are implemented using the scikit-learn library [32], ensuring consistency and reproducibility. Hyperparameter tuning is done at the inner loop of the validation. The parameter combinations considered for each model are available at Table 2.

Baseline classification and influence of normalization

To perform an initial assessment of the predictive power of microbiome features with and without normalization for the different diseases, we trained and validated 5 ML models (Random Forest, XGBoost, Logistic Regression, Support Vector Machine and K-Nearest Neighbor) on each dataset with the relative abundances (RA) approach. Then, we compared the results with the same models using Centered Log-Ratio (CLR) normalized data (see Methods section for details).

Focusing on the RA approach, trends on classifier performance are shown in figure 1. Tree-based models obtained best results across most datasets with Random Forest yielding the best AUC on average. Boosting (XGBoost) obtained the best performance at CDI dataset with an AUC of 0.926. Friedman's multiple comparisons test revealed statistical differences across algorithms and Finner's posthoc results shown at figure 2 confirmed this ranking, with Random Forest results significantly different when compared with all algorithms except for Boosting. K-Nearest Neighbors obtained the worst results, while not being statistically different from Support Vector Machine (SVM). Sitting in the middle ground, Logistic Regression obtained marginally better performance than SVM and slightly worse than Boosting.

Next, we performed the same analysis for data normalized using the CLR transformation. Results are shown in figures 3 and 4

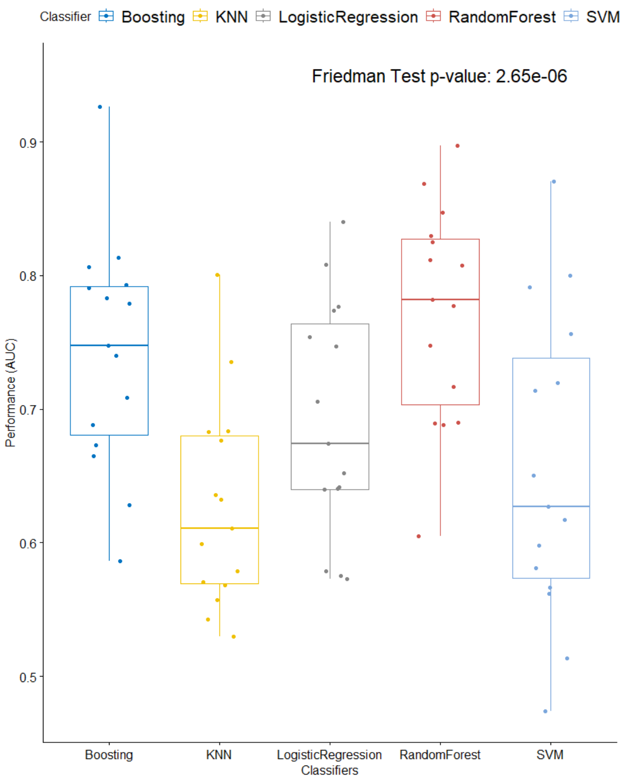

Figure 1. Classifier performance for relative abundance data

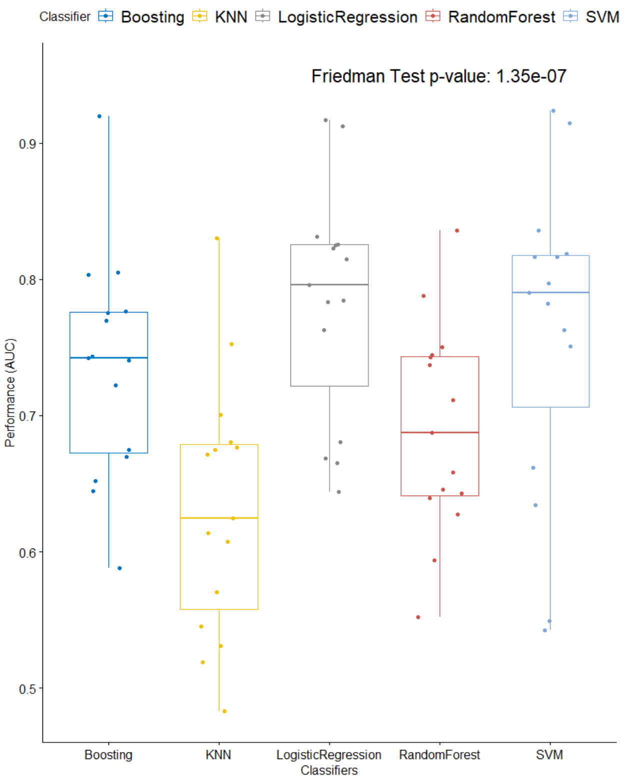

Figure 3. Classifier performance for CLR normalized data

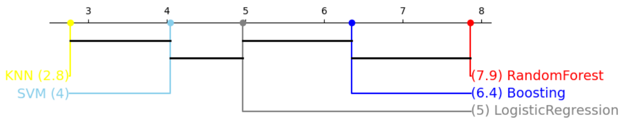

Figure 2. Finner's test results critical distance diagram for relative abundances approach

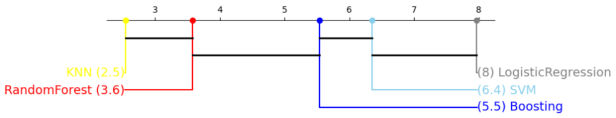

Figure 4. Finner's test results critical distance diagram for Centered Log-Ratio normalization approach

where trends clearly changed with Friedman's test again showing significant differences in performance. In this case, best models were Logistic Regression and Support Vector Machines. Boosting stays one step below as an stable alternative and Random Forest results dramatically fall into the fourth ranking position. One notable example of this drop in performance with normalization is the Obesity dataset which obtained an AUC of 0.869 for relative abundances and 0.788 for CLR normalization (note that Logistic Regression and SVM do improve RA results for this dataset).

On average, best performing models were Random Forests for RA data and Logistic Regression for CLR normalization (see AUC boxplots at figures 1 and 3 and Finner's test results at figures 2 and 4). AUC values varied between datasets showing which ones are more challenging (see Table 8), varying from the CDI dataset with AUC of 0.897 with Random Forest (with RA) and 0.917 with Logistic Regression (with CLR), to arthritis ART with an AUC of 0.605 with Random Forest (with RA) and 0.644 with Logistic Regression (with CLR).

We finally carried out a comparison between best models with and without normalization using Wilcoxon signed-rank test. As figure 5 shows, it can be observed that Logistic Regression comparison on both normalization methods revealed superior performance with CLR $p = 6.1 \cdot 10^{-5}$ (B) and the exact opposite trend in Random Forest comparison $p = 6.1 \cdot 10^{-5}$ (A). In both cases, the superior method outperformed its counterpart across all 15 datasets. Since the Wilcoxon test is rank-based, the p-values are identical. Comparing RA + Random Forest with CLR + Logistic Regression did not reveal differences $p = 0.56$ (C), suggesting different ways classifiers can be adapted to deal with compositional data.

To further investigate, we compared the means of all classifier results for each normalization method and found no significant difference in overall performance as for Wilcoxon test with $p = 0.055$ (figure 5 D), although the mean performance for CLR was slightly higher than for RA.

Feature Selection Performance Comparison

We also examined the predictive power of the biomarker sets provided by the different feature selection techniques. We considered mutual information, mRMR, LASSO, ReliefF and autoencoders (see Methods section). Table 3 shows top 10 performing Feature Selection + Classifier pipelines illustrating that the ones involving Logistic Regression outperformed the other methods. Thus, we observe that trends in classifier performance with CLR normalization (Figure 3) persisted after feature selection, with logistic regression as the best performing method across datasets. Furthermore, as shown in Table 4, Random forests continue to be the best classifier of those considered for RA data even after feature selection. However, the pipelines with RA are not competitive when compared with the CLR + FS + Logistic Regression pipelines. Wilcoxon test comparing the best classifier for each normalization across feature selection methods reaffirmed this superiority rejecting the null hypothesis with $p = 7.34 \cdot 10^{-13}$ as shown in Figure 6.

Additionally, in our search of the best ML pipeline we executed

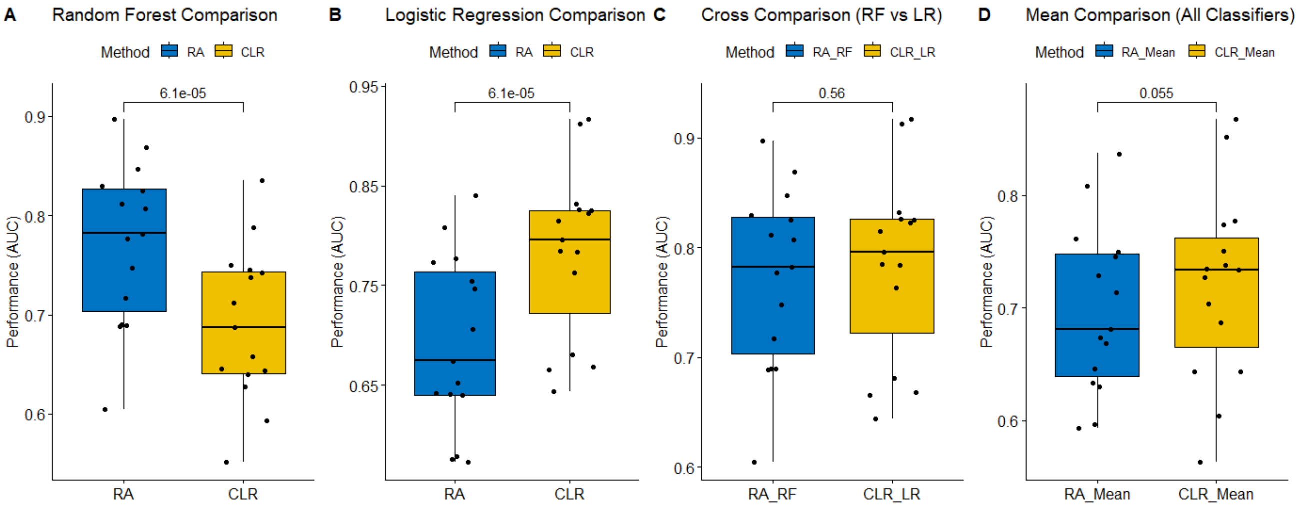

Figure 5. Wilcoxon test on normalization techniques. A. Random Forest comparison on both normalization methods. B. Logistic Regression comparison on both normalization methods. C. Comparing RA + Random Forest with CLR + Logistic Regression D. Mean results analysis across all classifiers, for both normalization methods.

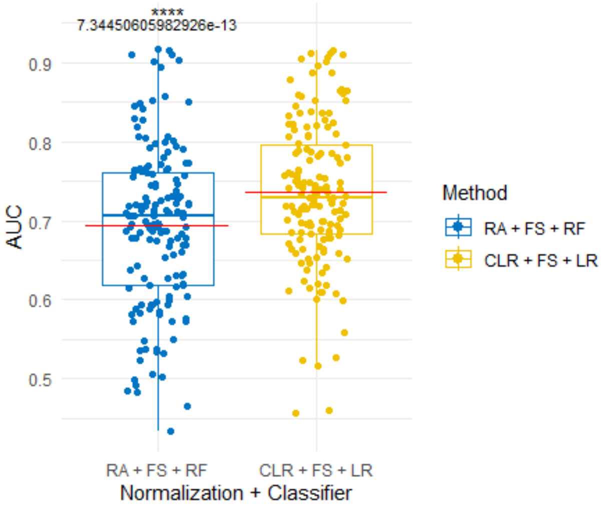

Figure 6. Wilcoxon signed-rank test assessing effects of normalization on feature selection pipelines considering best classifiers for each normalization(Random Forest for RA and Logistic Regression for CLR). Red lines indicate the means.

all combinations of Normalizations (RA or CLR) with the feature selection methods and classifiers selected. A total of 750 models were validated per normalization, and to observe effects of normalization on feature selection, we performed a paired Wilcoxon signed-rank test obtaining significant differences in favor of Centered Log-Ratio Normalization (see Supplementary figure S1) $p_{val} = 5.5 \cdot 10^{-74}$.

Relying on these results, we selected CLR + Logistic Regression as the reference to compare the feature selection algorithms on the normalized data, simplifying the analysis and enabling a more focused evaluation of their effectiveness.

Figure 7 and Table 5 show results of nested validation from this analysis. As expected, Friedman's multiple comparisons test revealed statistical differences with high significance $p_{val} = 9.14 \cdot 10^{-7}$. Two groups of methods are revealed in terms of results. The first group includes three algorithms with good performance at a comparable level: LASSO, mRMR50 and mRMR100, which consistently yield stronger results across datasets. On the other hand, the second group comprises six algorithms with lower mean performance, including ReliefF50, ReliefF100, variational autoencoder, MIFS50, MIFS100 and AE50, with the last one a step above the others. Finally, AE100 performance places it in a middle spot between the groups. Critical distance diagram at Figure 8 illustrates how Finner's posthoc test confirms these groups.

Model	Feature Selection	AUC
LogisticRegression	mRMR100	0.772
LogisticRegression	LASSO	0.768
LogisticRegression	mRMR50	0.764
SVM	LASSO	0.749
LogisticRegression	AE100	0.744
RandomForest	mRMR50	0.739
LogisticRegression	AE50	0.739
SVM	mRMR100	0.738
SVM	AE100	0.737
RandomForest	LASSO	0.737

Table 3. Top 10 machine learning pipelines sorted by mean AUC results across datasets for CLR normalization

Model	Feature Selection	AUC
RandomForest	mRMR100	0.757
RandomForest	Relief100	0.745
RandomForest	Relief50	0.744
RandomForest	mRMR50	0.737
RandomForest	LASSO	0.737
LogisticRegression	mRMR100	0.717
Boosting	mRMR100	0.715
LogisticRegression	mRMR50	0.713
Boosting	Relief50	0.706
Boosting	Relief100	0.704

Table 4. Top 10 machine learning pipelines sorted by AUC results across datasets for Relative Abundance data

The two variants of the minimum redundancy maximum relevance algorithm (50 and 100) achieved the highest AUC scores on a big number of datasets. Small differences were found when varying the number of selected features, indicating that fewer than 50 features may be sufficient for some datasets. Finner's test showed significant differences between them and their MIFS counterparts. Results on some datasets like Obesity or CD1 improve when using top 100 features suggesting a more detailed analysis should be done when building a model with the mRMR algorithm.

Mutual Information (MIFS) selection achieves moderate results

Table 5. Feature selection + Logistic Regression cross validation results for data normalized with the Centered Log-Ratio transformation

Dataset	AE50	AE100	VAE	Relief50	Relief100	MIFS50	MIFS100	mRMR50	mRMR100	LASSO
CDI	0.8869	0.8878	0.8617	0.9123	0.9107	0.9049	0.9124	0.8968	0.9022	**0.9152**
CIR	0.6542	0.6602	0.5233	0.7479	0.7410	0.6942	0.7318	**0.8371**	0.8245	0.8103
CRC1	0.6817	0.6675	0.6584	0.6830	0.6849	0.6144	0.6189	**0.7112**	0.7022	0.6470
CRC2	0.8206	0.7862	0.7188	0.7249	0.7452	0.7403	0.7550	0.7504	0.7575	**0.7606**
CD1	0.7770	0.7885	0.7277	0.7424	0.7445	0.7857	0.7852	0.7865	**0.8069**	0.7968
CD2	0.8372	0.8380	**0.8569**	0.8154	0.8184	0.8371	0.8269	0.8525	0.8459	0.8215
HIV	0.7079	0.7404	0.6092	0.6987	0.6936	0.7194	0.7299	0.7364	0.7270	**0.8336**
IBD1	0.7911	0.8077	0.7148	0.8065	0.7960	0.7512	0.7619	0.8180	0.8225	**0.8653**
IBD2	0.6413	0.6197	0.6512	0.6647	0.6783	0.6942	**0.6998**	0.6961	0.6979	0.6842
MHE	0.6523	0.6884	0.6771	0.4566	0.4604	0.7110	0.7157	0.7183	**0.7651**	0.7191
OB	0.8589	**0.9060**	0.8671	0.7817	0.7805	0.7417	0.7967	0.8529	0.8791	0.8650
PAR1	0.6636	0.6459	0.6341	0.6888	0.7002	0.5981	0.6227	0.7130	**0.7293**	0.6787
PAR2	0.7291	**0.7529**	0.7197	0.6754	0.6861	0.6638	0.6964	0.7213	0.7219	0.7480
PAR3	0.7403	0.7569	0.6973	0.7081	0.7147	0.7206	0.7482	0.7724	**0.7804**	0.7596
ART	0.6368	0.6098	0.5590	0.6581	**0.6717**	0.5270	0.5159	0.6000	0.6115	0.6077

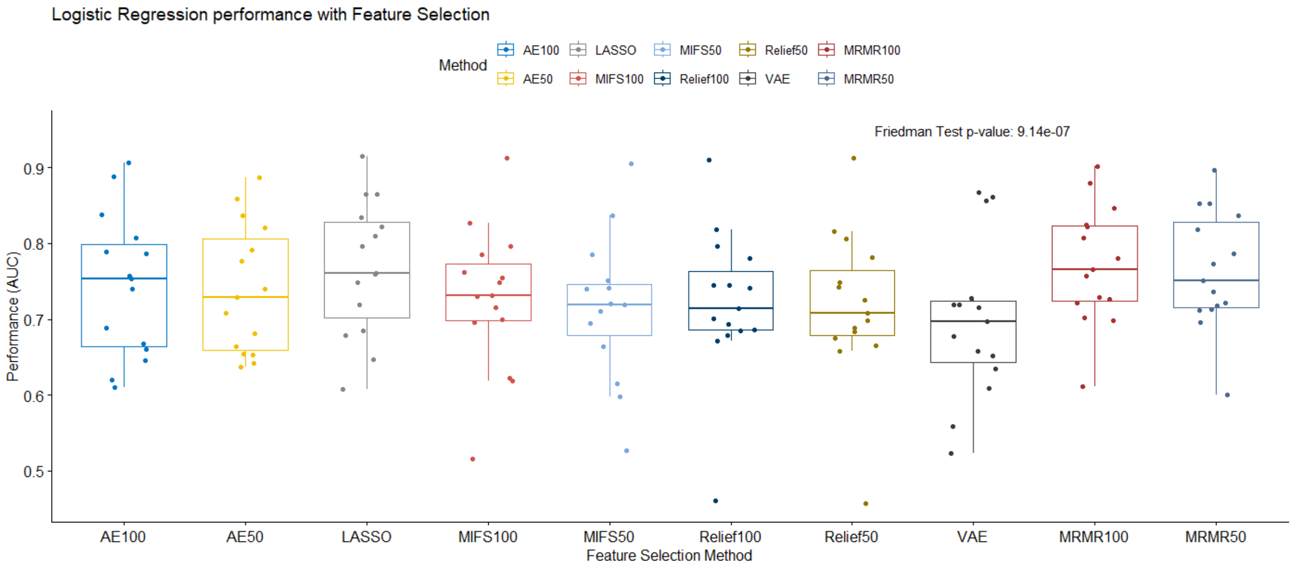

Figure 7. CLR + Feature Selection + Logistic Regression performance comparison

in feature selection, but as expected, tends to suffer from significant redundancy with MIFS100 not being significantly better than MIFS50 (see figure 8).

LASSO-based feature selection emerges as a competitive alternative to mRMR, achieving robust AUC scores (the highest at CDI, CRC2, HIV and IBD1) that are very close to the best-performing method, with no significant differences found compared with mRMR.

Relief-based methods do not perform particularly well on these datasets except for some interesting exceptions. On CDI and Arthritis (RA) datasets they obtained top results, with some difference compared with other feature selection methods in the case of Arthritis (AUC = 0.672).

Autoencoders results placed them on a middle ground in terms of performance, but with certain limitations. In particular, Finner's test did not show significant differences for AE100, neither compared with the top-performing methods nor with some of the weaker ones, such as ReliefF50. When constrained to a latent space of 50 dimensions (AE50) performance was less favorable. Finally, variational Autoencoders (VAE) displayed very poor performance on most datasets.

For the sake of completeness, we also analyzed the feature selection performance using the rest of classifiers. Mean AUC results across datasets are available at Supplementary Table 4. The results for SVM and Boosting are similar to those of Logistic Regression, with mRMR100, LASSO, and mRMR50 performing the best. RF performed particularly well with Relief, while achieving performance comparable to mRMR for this classifier, metrics were still far from the Logistic Regression pipeline results. KNN results are aligned with its baseline performance, showing consistently lower AUC scores compared to the other classifier pipelines.

To statistically validate these findings, we conducted a multiple comparison test by grouping classifier results based on feature selection algorithms. The Friedman test confirmed significant differences between groups ($p = 1.1 \cdot 10^{-16}$), and the Finner post hoc test (Supplementary Figure S2) indicated that while LASSO performed slightly better than mRMR across classifiers, the difference was not statistically significant. Notably, the observed trends align with those of Logistic Regression, with a more compact middle group consisting of Relief, MIFS100, and autoencoders. Finally, VAE and MIFS50 exhibited even weaker performance in this comparison.

mRMR selection outperforms Mutual Information

Motivated by the observation that mRMR100 was the best-performing method in our global comparison, we conducted a focused analysis of mRMR for its potential to extract smaller and more interpretable biomarker sets. We compared mRMR with Mutual Information (MI) for feature selection, employing an incremental approach where two new variables from the feature selection ranking were added at a time to evaluate their contributions (see figure

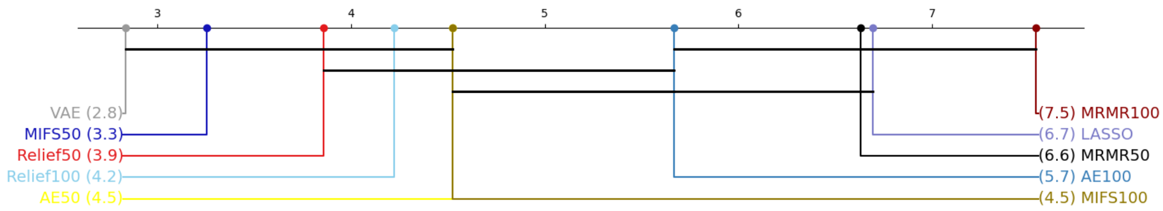

Figure 8. Finner's test results critical distance diagram for Feature selection + Logistic Regression pipelines with CLR normalization. Ranks from Friedman's test with p-value $= 9.14 \cdot 10^{-7}$

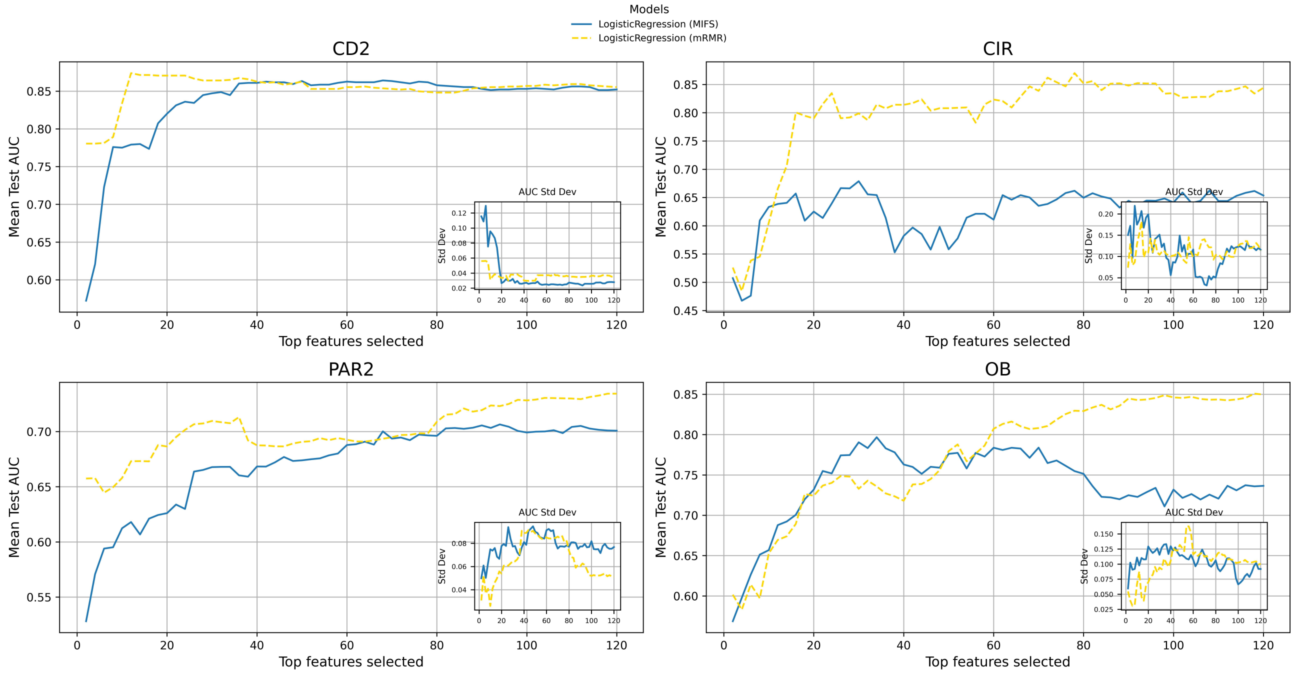

Figure 9. Cross Validation of Logistic Regression models with incremental number of features provided by Mutual Information and mRMR. Datasets CD2 and CIR provide examples of optimal biomarker sets with less than 50 features. PAR2 and OB sets keep improving beyond 50 and 100 features.

9).

The results on most datasets demonstrated that mRMR consistently outperforms MI in identifying biomarkers, with mRMR-selected features yielding better performance. This incremental approach is aligned with our statistical analysis, which showed significant differences between mRMR and MI for both small (50 features) and larger (100 features) subsets.

Two different trends are shown at figure 9. CD2 and CIR plots illustrate that in some scenarios, less than 50 features are enough to obtain near optimal sets, while PAR2 and OB model performance keeps improving when adding more than 50 features

LASSO performance remarks

Classic LASSO feature selection has also been applied to 16S compositional data, being the CLR normalization the most straightforward preprocessing approach [33]. As we showed before, relative abundances did not fit well this method. Our results show that this method achieves comparable performance across datasets against more complex feature selection algorithms. No statistical differences were found with well performing methods like mRMR (see posthoc test results in Figure 8).

However, LASSO tends to select a larger number of features compared to methods like mRMR, particularly in larger datasets. As shown in Table 6 , the number of selected features by LASSO is higher in datasets with more samples. This raised the need to verify whether using only the top-50 or top-100 features selected by LASSO would be sufficient to approach the best performance.

Dataset	Selected Features	Features	Samples
CRC2	60.32	837	102(46, 56)
IBD2	71.12	1496	114(68, 46)
IBD1	56.28	2742	91(67, 24)
CD1	70.32	3547	140(78, 62)
CD2	75.60	3547	160(68, 92)
CDI	115.88	3456	336(93, 243)
CIR	47.88	3104	77(51, 26)
MHE	51.00	3104	77(26, 51)
OB	126.44	6386	281(220, 61)
CRC1	217.68	6920	490(229, 261)
PAR2	188.72	6844	333(201, 132)
PAR1	100.52	10232	148(74, 74)
ART	75.32	10733	114(86, 28)
PAR3	295.96	12198	507(323, 184)
HIV	143.84	14425	350(293, 57)
Mean	**113.13**		

Table 6. Number of features selected by LASSO (mean of the cross validation), sorted by number of features of the dataset.

The implementation we used takes 10^{-5} as threshold to consider the absolute importance of a feature to be shrunk to zero and not

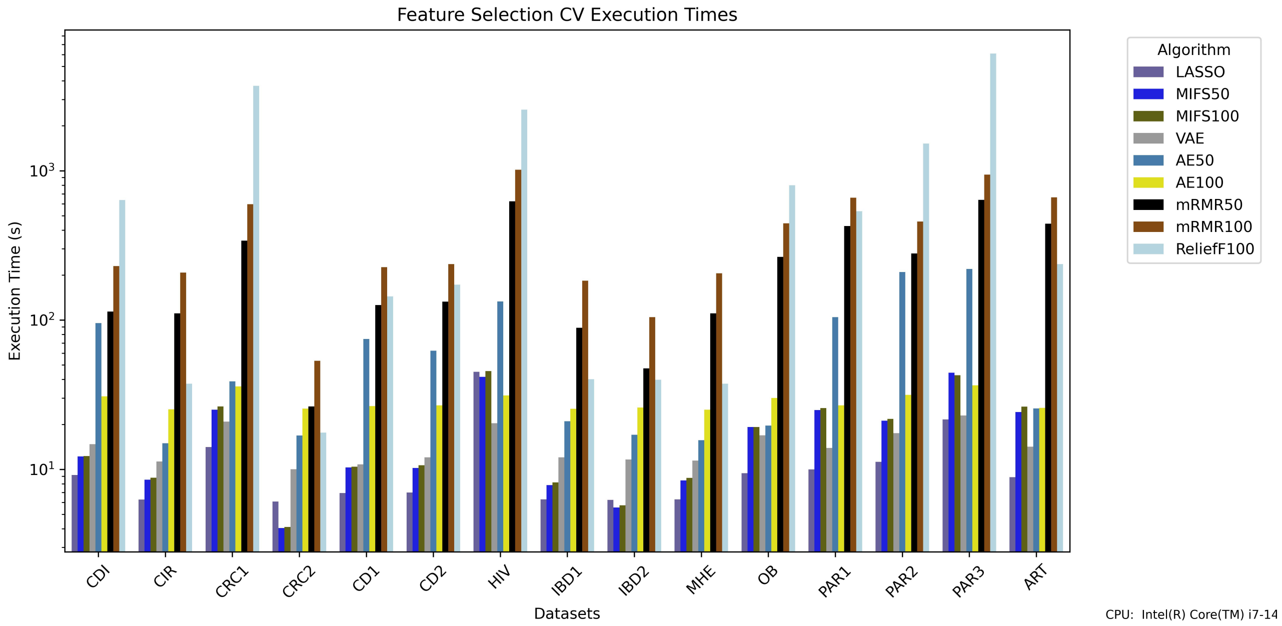

Figure 10. Mean execution time by fold at the outer Cross Validation by Feature Selection algorithm and dataset

selected. To assess the influence of this parameter, we repeated the analysis taking only the 50 and 100 most important variables, instead of all the variables not shrunk to zero. Results at Supplementary Table T7 show that differences between the threshold approach and the Top-100 approach are minimal. Even selecting the top 50 features is enough to achieve similar results for most datasets considered.

We also measured computing time performance for feature selection algorithms (see figure 10). LASSO stands out as the quickest, efficiently handling large datasets. Mutual Information and Variational Autoencoders (VAE) also performed swiftly. In contrast, algorithms like Minimum Redundancy Maximum Relevance (mRMR) and Autoencoders fall into an intermediate speed category; they were slower than LASSO but still manageable for moderate datasets. On the other hand, ReliefF was noticeably slower, specially with larger sample sizes.

Parkinson Cross-cohort Generalization

In order to assess the proposed pipelines on microbiome datasets, and further see its operation in identifying microbial signatures on even external datasets, we used datasets PAR2 and PAR3, which targeted the same hypervariable region. However, their dimensionality differed, with 6844 features at PAR2 and 12198 at PAR3. To address this difference, we trained models using the 2360 features shared between them. While both datasets were collected following uniform protocols, the analysis performed at [30] revealed batch effects between datasets. Thus, to avoid confounding we did not mix datasets while training, instead we trained the pipelines in one dataset and tested their generalization score in the other in a cross-cohort scheme.

We independently trained logistic regression models with feature selection using mRMR and LASSO on the subset of 2360 intersectional features. We performed an incremental analysis, evaluating classification performance by sequentially adding two features at a time (see figure 11). Comparing the performance of features selected by mRMR and LASSO revealed that mRMR-selected features exhibited better generalization in this case study.

Pipelines trained with dataset PAR3 and tested at PAR2 achieved 0.722 AUC with only 10 microbial markers, reaching 0.743 AUC with 100 for the mRMR+LR pipeline. However, LASSO+LR only reached 0.659 AUC with the top 100 selected features. Conversely, pipelines trained with dataset PAR2 and tested at PAR3 achieved similar performance for mRMR and LASSO with the top 50 features. Again, when considering 100 features, mRMR+LR pipeline obtained an AUC of 0.715 and LASSO+LR only obtained an AUC of 0.675.

Discussion

Normalization methods play a crucial role in microbiome data analysis, influencing the performance of classification tasks. A recent study showed that CLR normalization consistently outperforms RA for logistic regression models when classifying microbiome data [34]. Our findings corroborate this result for logistic regression, with CLR normalization yielding superior performance over RA across datasets. Similarly, but with slightly worse performance, SVM exhibited the same behavior across normalization methods. However, we proved this trend does not generalize across all classifiers.

We extended the evaluation of normalization methods to include multiple classifiers, revealing that random forest performs exceptionally well with RA while performing poorly with CLR normalization. This result underscores the importance of tailoring normalization methods to specific algorithms, as different models exhibit varying sensitivities to the transformed data distribution. Random Forest good performance using relative abundance data is aligned with the comparative conducted at [35], where CLR transformation was not applied. A plausible explanation is that decision trees, the core components of Random Forest and Boosting, operate by splitting data with decision thresholds, making them less affected by the compositional and interdependent nature of the data.

Moreover, the comparison between best classifiers for both normalization techniques (RA+RF and CLR+LR) did not reveal a big impact on performance. This suggests that while method-specific normalization optimizations can enhance individual classifier outcomes, the overall performance remains stable across different approaches for baseline disease classification.

Boosting and K-Nearest Neighbors displayed stable performance across normalization methods while they do not achieve

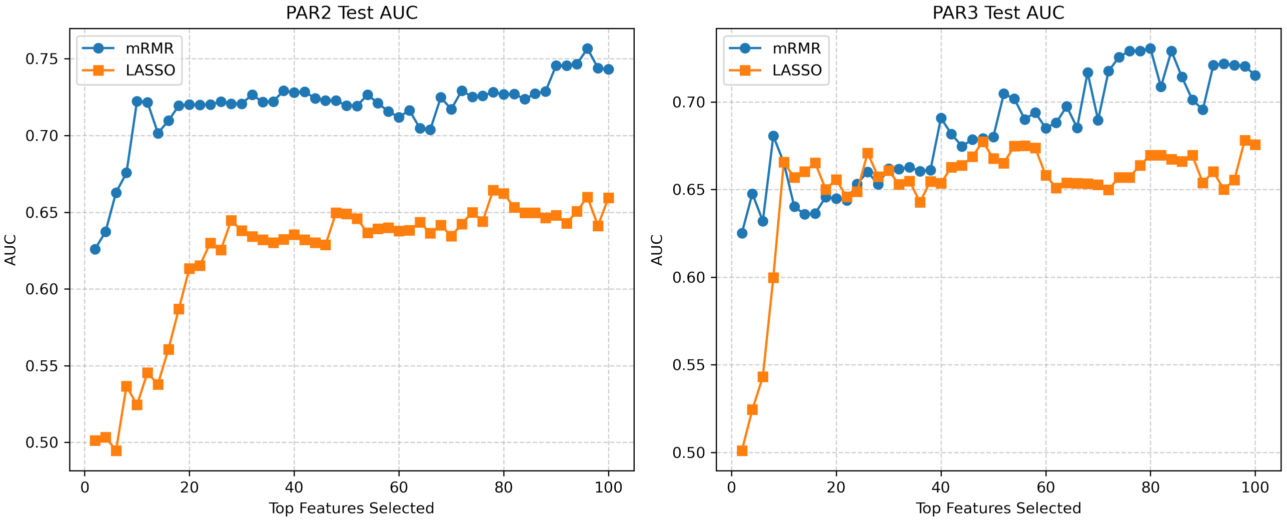

Figure 11. Cross-cohort performance comparison for Parkinson datasets (PAR2 and PAR3). Logistic Regression models were trained in one dataset and tested in the other, this plot shows the test AUCs considering top features incrementally.

top results in any of them. KNN generally underperformed relative to other classifiers (see figures 1 and 3). This result is likely attributable to the sparsity inherent in microbiome data, which poses challenges for distance-based algorithms like KNN. This underperformance is not addressed by CLR normalization, as the root issue seems to lie in the high sparsity of the dataset rather than the compositional nature of the data.

While these findings highlight the critical interplay between normalization strategies and machine learning algorithms, they also paved the way for further analysis. Wilcoxon paired test and boxplot from figure 6 revealed that CLR transformation enables a better-performing feature selection. This means that for most pipelines considered (Feature Selection + Classifier), CLR normalization performs better than the relative abundance approach. This enhancement in performance can be attributed to the way CLR transformation addresses the compositional issue. Data is isometrically transformed from the simplex into a hyperplane in the euclidean space, which makes unconstrained statistical methods applicable [36], thus likely making data more suitable to feature selection methods that implicitly use these techniques.

In this study, we examined the predictive power of biomarker sets identified through various feature selection techniques, using a comprehensive evaluation of classifier performance. The trends in classifier performance with CLR normalization persisted after feature selection, with Logistic Regression emerging as the top-performing method across datasets. Table 3 highlights the top 10 performing Feature Selection + Classifier pipelines, illustrating that Logistic Regression pipelines consistently outperform others.

When comparing feature selection methods, results shown at table 5 and critical distance diagram (figure 8) state clearly that mRMR100 and LASSO obtained top performance on most datasets.

mRMR selection obtains compact biomarker sets

The superior performance of mRMR can be attributed to the balance of its design that generates more informative and compact biomarker sets, which in some scenarios can be smaller, as evidenced by the incremental analysis we conducted (figure 9). These findings highlight mRMR's value as a robust and interpretable feature selection method for biomarker discovery in complex microbiome datasets.

A notable example of mRMR's effectiveness in biomarker discovery is demonstrated in the dataset PAR3, where the method identified a highly informative subset of less than 50 genera. These biomarkers exhibited strong associations with the target condition, achieving comparable performance to larger feature sets selected by other methods. Furthermore, these findings are consistent with results reported in the original study by [30], which highlighted the significance of reduced abundance of the genera Roseburia and Butyricicoccus, as well as the family Lachnospiraceae, in Parkinson's disease cases, alongside an observed overabundance of Bifidobacterium. All of these bacteria were found in the top 12 ranking provided by mRMR (table 7).

This result remarks the power of mRMR in focusing on the most relevant taxa, while minimizing redundancy coming from the hierarchical relationships between species of bacteria. Thus, resulting in compact biologically meaningful sets of features. The interpretability of this subsets is particularly valuable for translational applications, as it provides clear targets for further biological or clinical validation.

Autoencoders struggle to trade off performance and feature reduction

During experimentation, we tested various autoencoder architectures to determine the optimal configuration for microbiome feature selection. The modest results obtained by AE50 and AE100 indicated that larger latent spaces are required to achieve superior classification performance. However, this improvement would came at the cost of reduced interpretability and the necessity of handling a high-dimensional latent representation.

Additionally, we experimented with a Variational Autoencoder architecture, which is designed to encode input data into a probabilistic latent space. Despite its theoretical advantages, such as generating smoother latent spaces and capturing underlying data distributions, the VAE did not perform well on our datasets. It failed to effectively model the complexity of the microbiome data, resulting in worse classification outcomes compared to traditional autoencoders.

A significant limitation of autoencoders, particularly in the context of microbiome research and medical applications, is their inherent lack of interpretability. The latent space representations they generate are often considered "black boxes," making it difficult to directly link features to biological phenomena or actionable insights. While some methods for interpreting autoencoder outputs exist, they remain limited in scope and application [37, 38].

This lack of transparency poses a challenge, especially in healthcare-related fields where decision-making algorithms are increasingly scrutinized for explainability [39].

Taking into account these considerations, autoencoders may not

Phylum	Class	Order	Family	Genus
Firmicutes	Clostridia	Clostridiales	Lachnospiraceae	Roseburia
Firmicutes	Clostridia	Clostridiales	Family_XI	Ezakiella
Firmicutes	Bacilli	Bacillales	Not assigned	Not assigned
Firmicutes	Clostridia	Clostridiales	Ruminococcaceae	Butyricicoccus
Firmicutes	Clostridia	Clostridiales	Lachnospiraceae	Lachnoclostridium
Firmicutes	Bacilli	Lactobacillales	Streptococcaceae	Streptococcus
Proteobacteria	Gammaproteobacteria	Enterobacteriales	Enterobacteriaceae	Escherichia/Shigella
Proteobacteria	Gammaproteobacteria	Betaproteobacteriales	Burkholderiaceae	Delftia
Firmicutes	Clostridia	Clostridiales	Ruminococcaceae	Ruminococcaceae_UCG-013
Firmicutes	Bacilli	Lactobacillales	Streptococcaceae	Streptococcus
Actinobacteria	Actinobacteria	Bifidobacteriales	Bifidobacteriaceae	Bifidobacterium
Firmicutes	Clostridia	Clostridiales	Lachnospiraceae	Lachnospiraceae_UCG-004

Table 7. Top 12 taxa ranking provided by mRMR feature selection after CLR for PAR3 dataset.

be the most suitable alternative for identifying biologically meaningful signature sets. Simpler and more transparent methods, such as mRMR and LASSO, may be better suited for this specific task.

LASSO remains as a powerful option

Lasso feature selection has shown to perform well at microbiome-based disease classification paired with Logistic Regression and Random Forests for inflammatory bowel disease [40, 41].

Our results show that this method achieves comparable performance across datasets against more complex feature selection algorithms. When executed with big feature spaces it often selected a larger number of features compared to other methods (table 6), thus obtaining more complex biomarker sets. However, our analyses showed that taking top-50 and top-100 features obtained similar performance (Supplementary Table T7) and should be considered as a strong option.

Overall, the mRMR100 + Logistic Regression and Lasso + Logistic Regression pipelines (with CLR) proved to be powerful strategies for improving the interpretability and quality of the models. Table 8 shows that both feature selection pipelines achieve at least similar performance, if not better, than the best baseline classification models. Moreover, feature selection offers a greater advantage, a massive reduction of the dimensionality. As we can see, a reduction of around 99 % of the features can be achieved and the selected ones are able to display similar predictive performance as using all of them. This reduction help models to reduce overfitting and avoid learning noise or spurious patterns coming from features that are irrelevant to the biological signal. Additionally, a reduced set of features makes it easier to interpret and understand the underlying factors influencing the model's predictions.

Generalizing Across Cohorts

One of the key challenges in microbiome data analysis is to obtain feature sets that capture generalizable patterns, ensuring that the selected features are robust and applicable across different datasets rather than being influenced by specific dataset characteristics. For this reason, we conducted the cross-cohort study using our pipelines with the Parkinson data. It is important to note that this analysis was performed as a proof of concept. While the observed results are promising, the ability to consistently achieve such levels of generalization remains as an open question. To the best of our knowledge, although cross-cohort generalization is a desirable goal, it is rarely reported in 16S microbiome studies.

Our results show that the biomarkers obtained in this case study can reach a good level of generalization. Training with the 2360 intersectional features, despite being a constraint imposed by dataset characteristics, was not detrimental, at least for the mRMR+LR pipeline. In particular, when training on the PAR3 dataset and testing on PAR2, the achieved AUC matched the results obtained when both training and testing were conducted on PAR2 (see Table 5). The mRMR algorithm was able to identify microbial markers that generalized well across datasets, achieving higher external AUC scores than LASSO. The improved generalization of mRMR-selected features suggests that its greedy procedure to remove redundancy also can help to mitigate dataset-specific biases, allowing for more general biomarker discovery.

Another key observation is that models trained on PAR3 and tested on PAR2 achieved better generalization than the reverse. This is a reasonable outcome given the larger sample size in PAR3, which provides a broader representation of microbial variability at the training step. Larger datasets tend to capture more robust patterns, making them able to train models with lower generalization error.

Dataset	RA + RF	CLR + LR	CLR + LASSO + LR	CLR + mRMR100 + LR	Input Features	Reduction (%)
CDI	0.8973	0.9171	0.9152	0.9022	3456	97.1065
CIR	0.8468	0.8147	0.8103	0.8245	3104	96.7784
CRC1	0.6882	0.6651	0.6470	0.7022	6920	98.5549
CRC2	0.8247	0.7960	0.7606	0.7575	837	88.0526
CD1	0.8073	0.8253	0.7968	0.8069	3547	97.1807
CD2	0.8297	0.8229	0.8215	0.8459	3547	97.1807
HIV	0.7165	0.8257	0.8336	0.7270	14425	99.3068
IBD1	0.7818	0.8314	0.8652	0.8225	2742	96.3530
IBD2	0.6893	0.6683	0.6842	0.6979	1496	93.3155
MHE	0.8116	0.7835	0.7191	0.7651	3104	96.7784
OB	0.8686	0.9123	0.8650	0.8791	6386	98.4341
PAR1	0.6898	0.6806	0.6787	0.7293	10232	99.0227
PAR2	0.7476	0.7629	0.7480	0.7219	6844	98.5389
PAR3	0.7770	0.7844	0.7596	0.7804	12198	99.1802
ART	0.6048	0.6439	0.6077	0.6115	10733	99.0683

Table 8. AUC Performance comparison of different pipelines on microbiome datasets with feature selection. % of reduction computed using top-100 selected variables.

Potential implications

The findings of this study offer new lines of research that could expand the scope and applicability of microbiome disease classification pipelines. The following potential implications highlight broader research opportunities:

- **Hybrid Approaches:** The different feature selection methods explored could be complemented by hybrid strategies, such as employing efficient greedy methods for initial filtering, followed by computationally-intensive techniques like evolutionary algorithms to refine the microbial signatures. Such combinations may obtain very reduced yet informative feature subsets.
- **Evaluation Across Harmonized Datasets:** The harmonization of datasets provides an opportunity for comprehensive meta-analyses. Evaluating feature selection methods across integrated datasets can help generalize findings, ensuring they are robust to variations in data collection, preprocessing, and experimental conditions. Validation of results across external studies would further establish the reliability of identified biomarkers.
- **Advancing Explainability for Clinical Integration:** Developing explainability interfaces and metrics tailored to microbiome data can help to close the gap between bioinformatic discoveries and practical clinical applications. By enabling to understand the reasons behind feature selection and classification decisions, these tools would promote confidence in utilizing biomarkers for aiding diagnostics.
- **Towards Standardization and Collaboration:** As microbiome research continues to expand, standardization of feature selection practices, facilitated by reproducible workflows, will be essential. Collaborative platforms that integrate harmonized data, past results and interpretability frameworks could accelerate discoveries in microbiome research.

Conclusion

Taken together, our study provides some recommendations on how to improve biomarker discovery in 16S microbiome disease classification without sacrificing performance. Random Forests showed strong behavior for raw relative abundances and turned out to be good baselines for pure performance. On the other hand, CLR normalization combined with Logistic Regression helps to cope with compositionality at baseline classification and improves feature selection. Among feature selection methods, mRMR consistently enhanced predictive power while maintaining interpretability. LASSO must also be considered because of its similar performance and quicker computation.

Future studies are advised to match normalization and feature selection approaches to their chosen classifiers. They should also establish a trade-off between biomarker set sizes and predictive accuracy, in order to maximize the robustness and relevance of identified biomarkers.

Methods

16S rRNA data

The most usual sequencing technique to analyze human microbiome is 16S ribosomal RNA amplicon analysis [42]. The 16S gene is used for taxonomic and phylogenetic studies as it is universally present between different species of bacteria and archaea. It is approximately 1600 base pair long and contains nine hypervariable regions (V1-V9) with different conservation degree that can provide signatures of different bacterial species [43]. The conserved regions flanking the hypervariable region of interest can be used for PCR amplification of targets and posterior sequencing via the Illumina framework [44] or previously via the discontinued 454 pyrosequencing [45].

The resulting sequences are then clustered into operational taxonomic units (OTUs) [46] or resolved into amplicon sequence variants (ASVs) [47]. Finally, they are compared against a reference database in order to identify their likely taxonomy [48]. At this point raw data has transformed into an abundance table with the number of reads of each sequence for each sample, suited for data analysis and machine learning.

Preprocessing: Filtering and Normalization

As data selected was already assembled into OTU/ASV count tables, only simple filtering was needed. Datasets with acceptable number of features were not filtered to avoid removing low-count but possibly powerful predictive OTUs. In the other hand, extremely sparse datasets, with more than 15000 features were filtered (Supplementary Table T1 provides original feature dimensionality and details for the datasets), i.e., as in [35] we removed taxa with less than 10 reads across the dataset, and features that were not present in more than the 1 % of the dataset. Final dimensions of datasets are presented at table 1. Relative abundances of taxons were then computed, this step is commonly referred to as a closure operation. These proportions naturally reside in the simplex, a constrained D-dimensional space defined as:

$$\mathbf{x}_{\text{ra}} = \left[\frac{x_1}{\sum_{i=1}^{D} x_i}, \frac{x_2}{\sum_{i=1}^{D} x_i}, \ldots, \frac{x_D}{\sum_{i=1}^{D} x_i} \right] \in \mathcal{S}^{D-1}$$

$$\mathcal{S}^{D-1} = \left\{ \boldsymbol{x} \in \mathbb{R}^D \mid x_i \geq 0, \sum_{i=1}^{D} x_i = 1 \right\}$$

Centered log-ratio normalization was applied to address the compositional nature of microbiome data, and we compared its performance with RA. Aitchison's CLR normalization [11] transforms compositional data from the simplex into the Euclidean space by taking the logarithm of each feature's relative abundance divided by the geometric mean $G(x)$ of all features within a sample.

$$\mathbf{x}_{clr} = \left[\log\left(\frac{x_{\text{ra}1}}{G(\mathbf{x}_{\text{ra}})}\right), \log\left(\frac{x_{\text{ra}2}}{G(\mathbf{x}_{\text{ra}})}\right), \ldots, \log\left(\frac{x_{\text{ra}D}}{G(\mathbf{x}_{\text{ra}})}\right) \right]$$

However, due to the presence of zero counts in microbiome datasets (common when taxa are not detected in a given sample) it is necessary to introduce a pseudocount to avoid undefined logarithmic calculations. The choice of pseudocount remains a critical yet unresolved issue. Different pseudocount values can impact the results, as they affect the ratios between features and distribution of the transformed data. While commonly used values such as 0.5, 1, or small constants close to zero are practical solutions, there is no consensus on an optimal choice. We applied a pseudocount $p = 0.5$ to all datasets as done in the limma-voom trend [49], to allow for the CLR normalization.

Feature Selection Techniques Overview

Feature selection is a critical step in high-dimensional data analysis, as it reduces the number of variables while retaining the most informative ones. This enhances interpretability and may improve model performance. By selecting a small number of features, such as microbial genera or species, their biological relationships with the target diseases can be more easily identified, helping biomarker

discovery. Removing redundant and irrelevant features can lead to an improvement of the generalization ability of the models.

In this study, we primarily focused on the filter approach, which evaluates features independently of any classification algorithm. This provides a model-agnostic ranking that can be applied across different classifiers while minimizing the risk of overfitting [50]. Additionally, we employed autoencoders to explore dimensionality reduction techniques. While not strictly a feature selection method, autoencoders identify compressed representations of the data without the use of class information. This deep learning approach is emerging as a growing-interest technique in the microbiome domain [51]. As a reference, we also included LASSO, a widely used technique in the few studies addressing this problem, to benchmark the performance of our approaches.

Mutual Information and mRMR

Mutual information (MI) is a fundamental concept in information theory that measures the mutual dependency between two variables [52]. It estimates how much knowledge about one variable is obtained by observing the other. One of the main advantages of MI lies in its ability to capture nonlinear relationships, unlike simpler correlation metrics that only account for linear ones. This flexibility is particularly useful in biological data, where complex interactions are common. However, MI-based feature selection provides a ranking of features that has not accounted for redundancy, leading to the selection of features that may be highly correlated and thus less informative collectively. We applied the implementation available at scikit-learn [32].

The Minimum Redundancy Maximum Relevancy (mRMR) algorithm was initially designed for gene selection in transcriptomics. It aims to strike a balance between selecting features that are maximally relevant to the target variable and minimally redundant with each other [53]. Unlike simpler mutual information-based methods [54], mRMR imposes additional constraints to avoid selecting correlated features, which is particularly useful in high-dimensional datasets. In particular, we considered two subset sizes to retain for comparisons (50 and 100), in this way we can also evaluate the redundancy present in the data. We applied the open source implementation available at [55].

This algorithm has demonstrated its effectiveness in identifying small, biologically meaningful subsets of biomarkers in transcriptomics, especially in cancer studies. Applications of mRMR to 16S rRNA microbiome data have yielded mixed results, often selecting relatively large feature sets [13].

ReliefF

ReliefF is a feature selection algorithm that extends the original Relief method improving its capabilities to handle unbalanced and noisy datasets [56]. This algorithm evaluates features by estimating their ability to distinguish between instances that are near each other (nearest hits and misses), leveraging a nearest-neighbor approach to assess the relevance of features. Unlike most filter methods, ReliefF considers interactions among features, making it particularly suitable for high-dimensional datasets where complex relationships may exist. We applied the ReBATE open source implementation [57].

Autoencoders

Autoencoders are a specialized class of artificial neural networks that can learn efficient compressed data representations without the information from the labels. Its architecture consists of two main parts, an encoder layer that aims to transform the data into a reduced representation, the latent space. From the output of this layer, a decoder tries to rebuild the original input. During training, the loss function measures the difference between input and output layers, by minimizing this reconstruction loss, the latent space captures the most important features of the input data. While autoencoders are not a traditional Feature Selection method, they have been pointed out as an interesting hypothesis for task-adapted dimensionality reduction that can cope with sparse matrices and low number of samples. [51, 58] . For our study we used DeepMicro [59] , a deep learning framework designed to allow an effective representation of microbiome profiles, to learn the latent space representation as a previous step for the classification task. In particular, we used the shallow autoencoders that have only one hidden layer, as they showed the most promising results in the DeepMicro case studies and also because of the small sample sizes of some of the considered datasets. We chose 2 different latent space sizes (50 and 100), allowing to compare with the other algorithms, and a variational autoencoder (50). DeepMicro also offers the later classification step, but to adapt autoencoders to our validation strategy we preferred to use only the latent representation learning step.

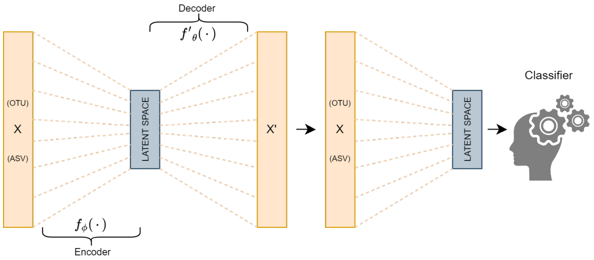

Figure 12. Shallow autoencoder feature reductor diagram. Latent space is calculated via optimization of the reconstruction loss $L(x, x') = ||x - x'||^2 = ||x - f'_\theta(f_\phi(x))||^2$.

LASSO

Least Absolute Shrinkage and Selection Operator (LASSO) is a regression-based method widely used for feature selection. By effectively shrinking the coefficients of less relevant features to zero it stands as a common FS method for high-dimensional datasets. In microbiome studies, LASSO is frequently employed either as a standalone feature selection method or as part of a pipeline paired with Logistic Regression [33, 60]. In our case, the Logistic Regression classifier uses L2-regularization, therefore when combined in a pipeline, both techniques will be hybridized.

Classification Models, Tuning and Validation

Our study leveraged both ensemble and traditional classification algorithms to evaluate the impact of feature selection and normalization techniques. Ensemble methods have been demonstrated as very powerful classifiers, among which Random Forests [25,26] (RF) and eXtreme Gradient Boosting decision trees [27] (XGBoost) are the two most popular ones. In addition to them, we employed traditional classifiers, including Support Vector Machines (SVM), Logistic Regression (LR) and k-Nearest Neighbors (KNN).

In order to get a robust measure of generalization performance, each model was validated through 5 times repeated nested 5 fold cross validation (see figure 13). To ensure fair comparisons, all classifiers underwent hyperparameter optimization using grid search in the inner validation loop. This computationally intensive process was selected to ensure robust performance estimates and minimize possible bias and potential overfitting from the model parameters selection [61].

The Area Under the Receiver Operating Characteristic Curve (AUC) was chosen as the objective metric, as it is the most commonly used performance measure in microbiome classification studies. AUC offers a general evaluation of model performance across varying decision thresholds, being less sensitive to imbalance in datasets, where accuracy may fail to capture the true predictive power of a classifier.

Statistical Analysis

Comparing results of multiple algorithms across multiple datasets requires the use of statistical tests to avoid reaching conclusions due to random chance [62].

To analyze pairwise differences between pairs of classifiers or normalization techniques, we employed the Wilcoxon signed-rank test. This paired non-parametric test was chosen due to its suitability for scenarios where the assumptions of normality of parametric tests may not hold.

We also employed the Friedman test followed by the Finner posthoc procedure to analyze the differences between multiple classifiers and feature selection methods. This approach was chosen by evidences on superior power compared to other procedures [63], which means it has a lower probability of making a type II error. The Friedman test allowed us to determine the presence of significant differences in classifier performance for both normalizations, while the Finner posthoc procedure enabled us to identify which methods differed from each other in a controlled manner.

Availability of source code and requirements

- Project name: 16SMicrobiomeMLFS
- Project home page: https://github.com/nach00gar/16SMicrobiomeMLFS
- Operating system(s): Platform independent
- Programming language: Python, R
- Other requirements: sklearn 1.5.2, composition_stats 2.0.0, xgboost 2.1.2, keras 2.2.4, mrmr-selection 0.2.8, skrebate 0.62, DeepMicro [59], stac (Statistical Tests for Algorithms Comparison [64])
- License: GNU GPL

Data availability

Supporting data used for the analyses can be accessed by our code repository. Data was downloaded from MicrobiomeHD [16], MLRepo [17] and [18] article repository.

Declarations

List of abbreviations

AE: Autoencoder; AUC: Area Under the Receiver Operating Characteristic Curve; ASV: Amplicon Sequence Variant; CLR: Centered Log-Ratio; FS: Feature Selection; IR: Imbalance Ratio; KNN: k-Nearest Neighbors; LASSO: Least Absolute Shrinkage and Selection Operator; LR: Logistic Regression; MIFS: Mutual Information Feature Selection; ML: Machine Learning; mRMR: Minimum Redundancy Maximum Relevancy; OTU: Operational Taxonomic Unit; PCR: Polymerase Chain Reaction; RA: Relative Abundances; RF: Random Forest; rRNA: Ribosomal RNA; SVM: Support Vector Machine; VAE: Variational Autoencoder.

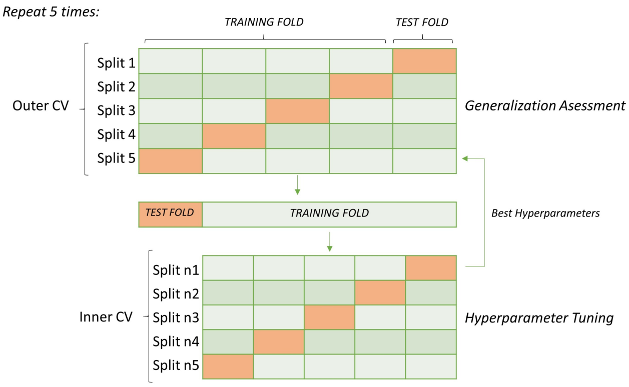

Figure 13. Repeated Nested Cross Validation procedure.

Disclosure of use of AI-assisted tools

Large language models [65] were used to improve the flow of certain paragraphs and to find grammatical errors. The use of the LLM does not negatively affect the empirical data and conclusions. The authors have thoroughly reviewed the text and are fully responsible for its content.

Competing Interests

The authors declare that they have no competing interests.

Funding

This work is supported by Grant PID2021-128317OB-I00 (Principal Investigators: Ignacio Rojas and Luis Javier Herrera) and PCI2023-146016-2 (Principal Investigators: Francisco Manuel Ortuño and Olga Valenzuela), funded by MICIU/AEI/10.13039/501100011033 and co-funded by the European Union.

Author's Contributions

Conceptualization, I.G., L.J.H. and F.M.O.; methodology, I.G. and L.J.H.; software, I.G.; analysis and validation, I.G.; data curation, I.G. ; writing—original draft preparation, I.G. and L.J.H.; writing—review and editing, I.R., L.J.H., and F.M.O.; supervision, I.R., L.J.H., and F.M.O.; funding acquisition, I.R., L.J.H., and F.M.O. All authors read and approved the final manuscript.

References

1. Hou K, Wu ZX, Chen XY, Wang JQ, Zhang D, Xiao C, et al. Microbiota in health and diseases. Signal Transduction and Targeted Therapy 2022 Apr;7(1):1–28. https://www.nature.com/articles/s41392-022-00974-4.
2. Akbar N, Khan NA, Muhammad JS, Siddiqui R. The role of gut microbiome in cancer genesis and cancer prevention. Health Sciences Review 2022 Mar;2:100010. https://www.sciencedirect.com/science/article/pii/S277263202100009X.
3. Núñez-Sánchez MA, Melgar S, O'Donoghue K, Martínez-Sánchez MA, Fernández-Ruiz VE, Ferrer-Gómez M, et al. Crohn's Disease, Host–Microbiota Interactions, and Immunonutrition: Dietary Strategies Targeting Gut Microbiome as Novel Therapeutic Approaches. International Journal of Molecular Sciences 2022 Jul;23(15):8361. https://www.ncbi.nlm.nih.gov/pmc/articles/PMC9369148/.
4. Zhao T, Wei Y, Zhu Y, Xie Z, Hai Q, Li Z, et al. Gut microbiota and rheumatoid arthritis: From pathogenesis to novel therapeutic opportunities. Frontiers in Immunology 2022 Sep;13:1007165. https://www.ncbi.nlm.nih.gov/pmc/articles/PMC9499173/.
5. Carabotti M, Scirocco A, Maselli MA, Severi C. The gut-brain axis: interactions between enteric microbiota, central and enteric nervous systems. Annals of Gastroenterology: Quarterly Publication of the Hellenic Society of Gastroenterology 2015 Jun;28(2):203. https://pmc.ncbi.nlm.nih.gov/articles/PMC4367209/.
6. Hernández Medina R, Kutuzova S, Nielsen KN, Johansen J, Hansen LH, Nielsen M, et al. Machine learning and deep learn-

ing applications in microbiome research. ISME Communications 2022 Oct;2(1):1–7. https://www.nature.com/articles/s43705-022-00182-9.
7. Altman N, Krzywinski M. The curse(s) of dimensionality. Nature Methods 2018 Jun;15(6):399–400. https://www.nature.com/articles/s41592-018-0019-x.
8. Pan AY. Statistical analysis of microbiome data: The challenge of sparsity. Current Opinion in Endocrine and Metabolic Research 2021 Aug;19:35–40. https://www.sciencedirect.com/science/article/pii/S2451965021000600.
9. Gloor GB, Macklaim JM, Pawlowsky-Glahn V, Egozcue JJ. Microbiome Datasets Are Compositional: And This Is Not Optional. Frontiers in Microbiology 2017;8:2224.
10. Gloor GB, Wu JR, Pawlowsky-Glahn V, Egozcue JJ. It's all relative: analyzing microbiome data as compositions. Annals of Epidemiology 2016 May;26(5):322–329. https://www.sciencedirect.com/science/article/pii/S1047279716300734.
11. Aitchison J. The Statistical Analysis of Compositional Data. London: Chapman and Hall.; 1986.
12. Pödör Z, Hekfusz M. Comparing Feature Selection Methods on Metagenomic Data using Random Forest Classifier. Transactions on Engineering and Computing Sciences 2024 Feb;12(1):175–187. https://journals.scholarpublishing.org/index.php/TMLAI/article/view/16525.
13. Wu H, Cai L, Li D, Wang X, Zhao S, Zou F, et al. Metagenomics Biomarkers Selected for Prediction of Three Different Diseases in Chinese Population. BioMed Research International 2018;2018(1):2936257. https://onlinelibrary.wiley.com/doi/abs/10.1155/2018/2936257.
14. Human Microbiome Project Consortium. Structure, function and diversity of the healthy human microbiome. Nature 2012 Jun;486(7402):207–214.
15. McDonald D, Hyde E, Debelius JW, Morton JT, Gonzalez A, Ackermann G, et al. American Gut: an Open Platform for Citizen Science Microbiome Research. mSystems 2018 May;3(3):10.1128/msystems.00031–18. https://journals.asm.org/doi/10.1128/msystems.00031-18.
16. Duvallet C, Gibbons S, Gurry T, Irizarry R, Alm E, MicrobiomeHD: the human gut microbiome in health and disease. Zenodo; 2017. https://zenodo.org/records/569601, accessed 7 October 2024.
17. Vangay P, Hillmann BM, Knights D. Microbiome Learning Repo (ML Repo): A public repository of microbiome regression and classification tasks. GigaScience 2019 Apr;8(5):giz042. https://pmc.ncbi.nlm.nih.gov/articles/PMC6493971/.
18. Wallen ZD. Comparison study of differential abundance testing methods using two large Parkinson disease gut microbiome datasets derived from 16S amplicon sequencing. BMC Bioinformatics 2021 May;22(1):265. https://doi.org/10.1186/s12859-021-04193-6.
19. Scher JU, Sczesnak A, Longman RS, Segata N, Ubeda C, Bielski C, et al. Expansion of intestinal Prevotella copri correlates with enhanced susceptibility to arthritis. eLife 2013 Nov;2:e01202. https://doi.org/10.7554/eLife.01202.
20. Schubert AM, Rogers MAM, Ring C, Mogle J, Petrosino JP, Young VB, et al. Microbiome Data Distinguish Patients with Clostridium difficile Infection and Non-C. difficile-Associated Diarrhea from Healthy Controls. mBio 2014 May;5(3):10.1128/mbio.01021–14. https://journals.asm.org/doi/10.1128/mbio.01021-14.
21. Baxter NT, Ruffin MT, Rogers MAM, Schloss PD. Microbiota-based model improves the sensitivity of fecal immunochemical test for detecting colonic lesions. Genome Medicine 2016 Apr;8(1):37. https://doi.org/10.1186/s13073-016-0290-3.
22. Wang T, Cai G, Qiu Y, Fei N, Zhang M, Pang X, et al. Structural segregation of gut microbiota between colorectal cancer patients and healthy volunteers. The ISME Journal 2012 Feb;6(2):320–329. https://doi.org/10.1038/ismej.2011.109.
23. Noguera-Julian M, Rocafort M, Guillén Y, Rivera J, Casadellà M, Nowak P, et al. Gut Microbiota Linked to Sexual Preference and HIV Infection. eBioMedicine 2016 Mar;5:135–146. https://www.thelancet.com/journals/ebiom/article/PIIS2352-3964(16)30028-7/fulltext.
24. Gevers D, Kugathasan S, Denson LA, Vázquez-Baeza Y, Treuren WV, Ren B, et al. The treatment-naïve microbiome in new-onset Crohn's disease. Cell host & microbe 2014 Mar;15(3):382. https://pmc.ncbi.nlm.nih.gov/articles/PMC4059512/.
25. Papa E, Docktor M, Smillie C, Weber S, Preheim SP, Gevers D, et al. Non-Invasive Mapping of the Gastrointestinal Microbiota Identifies Children with Inflammatory Bowel Disease. PLOS ONE 2012 Jun;7(6):e39242. https://journals.plos.org/plosone/article?id=10.1371/journal.pone.0039242.
26. Willing BP, Dicksved J, Halfvarson J, Andersson AF, Lucio M, Zheng Z, et al. A pyrosequencing study in twins shows that gastrointestinal microbial profiles vary with inflammatory bowel disease phenotypes. Gastroenterology 2010;139(6):1844–1854.e1.
27. Zhang Z, Zhai H, Geng J, Yu R, Ren H, Fan H, et al. Large-Scale Survey of Gut Microbiota Associated With MHE Via 16S rRNA-Based Pyrosequencing. Official journal of the American College of Gastroenterology | ACG 2013 Oct;108(10):1601. https://journals.lww.com/ajg/abstract/2013/10000/large_scale_survey_of_gut_microbiota_associated.14.aspx.
28. Turnbaugh PJ, Hamady M, Yatsunenko T, Cantarel BL, Duncan A, Ley RE, et al. A core gut microbiome in obese and lean twins. Nature 2009 Jan;457(7228):480–484. https://www.nature.com/articles/nature07540.
29. Scheperjans F, Aho V, Pereira PAB, Koskinen K, Paulin L, Pekkonen E, et al. Gut microbiota are related to Parkinson's disease and clinical phenotype. Movement Disorders 2015;30(3):350–358. https://onlinelibrary.wiley.com/doi/abs/10.1002/mds.26069.
30. Wallen ZD, Appah M, Dean MN, Sesler CL, Factor SA, Molho E, et al. Characterizing dysbiosis of gut microbiome in PD: evidence for overabundance of opportunistic pathogens. npj Parkinson's Disease 2020 Jun;6(1):1–12. https://www.nature.com/articles/s41531-020-0112-6.
31. Hill-Burns EM, Debelius JW, Morton JT, Wissemann WT, Lewis MR, Wallen ZD, et al. Parkinson's Disease and PD Medications Have Distinct Signatures of the Gut Microbiome. Movement disorders: official journal of the Movement Disorder Society 2017 May;32(5):739–749. https://www.ncbi.nlm.nih.gov/pmc/articles/PMC5469442/.
32. Pedregosa F, Varoquaux G, Gramfort A, Michel V, Thirion B, Grisel O, et al. Scikit-learn: Machine Learning in Python. Journal of Machine Learning Research 2011;12(85):2825–2830. http://jmlr.org/papers/v12/pedregosa11a.html.
33. Susin A, Wang Y, Lê Cao KA, Calle ML. Variable selection in microbiome compositional data analysis. NAR Genomics and Bioinformatics 2020 Jun;2(2):lqaa029. https://doi.org/10.1093/nargab/lqaa029.
34. Quinn TP, Erb I. Interpretable Log Contrasts for the Classification of Health Biomarkers: a New Approach to Balance Selection. mSystems 2020 Apr;5(2):10.1128/msystems.00230–19. https://journals.asm.org/doi/10.1128/msystems.00230-19.
35. Wang XW, Liu YY. Comparative study of classifiers for human microbiome data. Medicine in Microecology 2020 Jun;4:100013. https://www.sciencedirect.com/science/article/pii/S2590097820300100.
36. Lin H, Peddada SD. Analysis of microbial compositions: a review of normalization and differential abundance analysis. NPJ Biofilms and Microbiomes 2020 Dec;6:60. https://www.ncbi.nlm.nih.gov/pmc/articles/PMC7710733/.
37. Li Q, Yu Y, Kossinna P, Lun T, Liao W, Zhang Q. XA4C:

eXplainable representation learning via Autoencoders revealing Critical genes. PLOS Computational Biology 2023 Oct;19(10):e1011476. https://journals.plos.org/ploscompbiol/article?id=10.1371/journal.pcbi.1011476.
38. Vivek S, Faul J, Thyagarajan B, Guan W. Explainable variational autoencoder (E-VAE) model using genome-wide SNPs to predict dementia. Journal of Biomedical Informatics 2023 Dec;148:104536. https://www.sciencedirect.com/science/article/pii/S1532046423002575.
39. Ali S, Abuhmed T, El-Sappagh S, Muhammad K, Alonso-Moral JM, Confalonieri R, et al. Explainable Artificial Intelligence (XAI): What we know and what is left to attain Trustworthy Artificial Intelligence. Information Fusion 2023 Nov;99:101805. https://www.sciencedirect.com/science/article/pii/S1566253523001148.
40. Hacılar H, Nalbantoğlu OU, Bakir-Güngör B. Machine Learning Analysis of Inflammatory Bowel Disease-Associated Metagenomics Dataset. In: 2018 3rd International Conference on Computer Science and Engineering (UBMK); 2018. p. 434–438. https://ieeexplore.ieee.org/document/8566487/?arnumber=8566487.
41. Fukui H, Nishida A, Matsuda S, Kira F, Watanabe S, Kuriyama M, et al. Usefulness of Machine Learning-Based Gut Microbiome Analysis for Identifying Patients with Irritable Bowels Syndrome. Journal of Clinical Medicine 2020 Aug;9(8):2403. https://www.mdpi.com/2077-0383/9/8/2403.
42. Johnson JS, Spakowicz DJ, Hong BY, Petersen LM, Demkowicz P, Chen L, et al. Evaluation of 16S rRNA gene sequencing for species and strain-level microbiome analysis. Nature Communications 2019 Nov;10(1):5029. https://www.nature.com/articles/s41467-019-13036-1.
43. Chakravorty S, Helb D, Burday M, Connell N, Alland D. A detailed analysis of 16S ribosomal RNA gene segments for the diagnosis of pathogenic bacteria. Journal of microbiological methods 2007 Feb;69(2):330. https://pmc.ncbi.nlm.nih.gov/articles/PMC2562909/.
44. Amplicon Sequencing Solutions;. https://emea.illumina.com/techniques/sequencing/dna-sequencing/targeted-resequencing/amplicon-sequencing.html, accessed 19 December 2024.
45. Rothberg JM, Leamon JH. The development and impact of 454 sequencing. Nature Biotechnology 2008 Oct;26(10):1117–1124.
46. Nguyen NP, Warnow T, Pop M, White B. A perspective on 16S rRNA operational taxonomic unit clustering using sequence similarity. npj Biofilms and Microbiomes 2016 Apr;2(1):1–8. https://www.nature.com/articles/npjbiofilms20164.
47. Callahan BJ, McMurdie PJ, Holmes SP. Exact sequence variants should replace operational taxonomic units in marker-gene data analysis. The ISME Journal 2017 Jul;11(12):2639. https://pmc.ncbi.nlm.nih.gov/articles/PMC5702726/.
48. Santamaria M, Fosso B, Consiglio A, De Caro G, Grillo G, Licciulli F, et al. Reference databases for taxonomic assignment in metagenomics. Briefings in Bioinformatics 2012 Nov;13(6):682–695. https://doi.org/10.1093/bib/bbs036.
49. Law CW, Chen Y, Shi W, Smyth GK. voom: precision weights unlock linear model analysis tools for RNA-seq read counts. Genome Biology 2014 Feb;15(2):R29. https://doi.org/10.1186/gb-2014-15-2-r29.
50. Guyon I, Elisseeff A. An introduction to variable and feature selection. J Mach Learn Res 2003 Mar;3(null):1157–1182.
51. Roy G, Prifti E, Belda E, Zucker JD. Deep learning methods in metagenomics: a review. Microbial Genomics 2024;10(4):001231. https://www.microbiologyresearch.org/content/journal/mgen/10.1099/mgen.0.001231.
52. Cover TM, Thomas JA. Elements of Information Theory. John Wiley & Sons; 2012.
53. Ding C, Peng H. Minimum redundancy feature selection from microarray gene expression data. In: Computational Systems Bioinformatics. CSB2003. Proceedings of the 2003 IEEE Bioinformatics Conference. CSB2003 Stanford, CA, USA: IEEE Comput. Soc; 2003. p. 523–528. http://ieeexplore.ieee.org/document/1227396/.
54. Kraskov A, Stögbauer H, Grassberger P. Estimating mutual information. Physical Review E, Statistical, Nonlinear, and Soft Matter Physics 2004 Jun;69(6 Pt 2):066138.
55. smazzanti, smazzanti/mrmr minimum Redundancy - Maximum Relevance; 2025. https://github.com/smazzanti/mrmr, installed 1 November 2024.
56. Kononenko I. Estimating attributes: Analysis and extensions of RELIEF. In: Bergadano F, De Raedt L, editors. Machine Learning: ECML-94 Berlin, Heidelberg: Springer; 1994. p. 171–182.
57. Urbanowicz RJ, Meeker M, La Cava W, Olson RS, Moore JH. Relief-based feature selection: Introduction and review. Journal of Biomedical Informatics 2018 Sep;85:189–203. https://www.sciencedirect.com/science/article/pii/S1532046418301400.
58. Wickramaratne D, Wijesinghe R, Weerasinghe R. Human Gut Microbiome Data Analysis for Disease Likelihood Prediction Using Autoencoders. In: 2021 21st International Conference on Advances in ICT for Emerging Regions (ICter); 2021. p. 49–54. https://ieeexplore.ieee.org/document/9774811/?arnumber=9774811, iSSN: 2472-7598.
59. Oh M, Zhang L. DeepMicro: deep representation learning for disease prediction based on microbiome data. Scientific Reports 2020 Apr;10(1):6026. https://www.nature.com/articles/s41598-020-63159-5.
60. Queen O, Emrich SJ. LASSO-based feature selection for improved microbial and microbiome classification. In: 2021 IEEE International Conference on Bioinformatics and Biomedicine (BIBM); 2021. p. 2301–2308. https://ieeexplore.ieee.org/document/9669485.
61. Cawley GC, Talbot NLC. On Over-fitting in Model Selection and Subsequent Selection Bias in Performance Evaluation. Journal of Machine Learning Research 2010;11(70):2079–2107. http://jmlr.org/papers/v11/cawley10a.html.
62. Demšar J. Statistical Comparisons of Classifiers over Multiple Data Sets. Journal of Machine Learning Research 2006;7(1):1–30. http://jmlr.org/papers/v7/demsar06a.html.
63. García S, Fernández A, Luengo J, Herrera F. Advanced nonparametric tests for multiple comparisons in the design of experiments in computational intelligence and data mining: Experimental analysis of power. Information Sciences 2010 May;180(10):2044–2064. https://www.sciencedirect.com/science/article/pii/S0020025509005404.
64. Rodríguez-Fdez I, Canosa A, Mucientes M, Bugarín A. STAC: A web platform for the comparison of algorithms using statistical tests. In: 2015 IEEE International Conference on Fuzzy Systems (FUZZ-IEEE); 2015. p. 1–8. https://ieeexplore.ieee.org/document/7337889.
65. OpenAI, ChatGpt (GPT-4 Turbo, November 2023)[Large language model]; 2024. chat.openai.com/chat, queried until 16/02/2025.

Click here to access/download
Supplementary Material
Supplementary Material.pdf
