# Supplementary material for: Exploring the role of normalization and feature selection in microbiome disease classification pipelines
Source: Gigascience. 2025 Sep 2;14:giaf096. doi: 10.1093/gigascience/giaf096 (PMC12402773; doi:10.1093/gigascience/giaf096)

GigaScience

Exploring the role of normalization and feature selection in microbiome disease classification pipelines

--Manuscript Draft--

**Manuscript Number:**	GIGA-D-25-00079R1
**Full Title:**	Exploring the role of normalization and feature selection in microbiome disease classification pipelines
**Article Type:**	Research
**Funding Information:**	Ministerio de Ciencia e Innovación (PID2021-128317OB-I00) — Prof. Ignacio Rojas Ruiz<br>Consejería de Conocimiento, Investigación y Universidad, Junta de Andalucía (PCI2023- 146016-2) — Dr. Francisco Manuel Ortuño Guzmán
**Abstract:**	Background: Disease classification using 16S rRNA microbiome data faces challenges of high dimensionality, compositionality, and sparsity, compounded by the inherent small sample sizes in many studies. Machine learning and feature selection techniques offer potential to identify robust biomarkers and improve classification performance; however, their comparative effectiveness across diverse methods and datasets has been insufficiently explored. This study evaluates multiple feature selection techniques alongside normalization strategies, focusing on their interplay with classifier performance. Results: Our analyses revealed that Centered Log-Ratio normalization improves the performance of Logistic Regression and Support Vector Machine models and facilitates feature selection, whereas RandomForest models yielded strong results using relative abundances. Interestingly, Presence-Absence normalization was able to achieve similar performance compared to abundance-based transformations across classifiers. Among feature selection methods, minimum redundancy maximum relevancy (mRMR) surpassed most methods in identifying compact feature sets and demonstrated performance comparable to LASSO, which obtained top results requiring lower computation times. Autoencoders needed larger latent spaces to perform well and lacked interpretability, Mutual Information suffered from redundancy, and ReliefF struggled with data sparsity. Conclusions: Overall, feature selection pipelines improved model focus and robustness via a massive reduction of the feature space. MRMR and LASSO emerged as the most effective methods across datasets.
**Corresponding Author:**	Ignacio Garach Vélez<br>Universidad de Granada Escuela Tecnica Superior de Ingenierias Informatica y de Telecomunicacion<br>GRANADA, GRANADA SPAIN
**Corresponding Author Secondary Information:**	
**Corresponding Author's Institution:**	Universidad de Granada Escuela Tecnica Superior de Ingenierias Informatica y de Telecomunicacion
**Corresponding Author's Secondary Institution:**	
**First Author:**	Ignacio Garach Vélez
**First Author Secondary Information:**	
**Order of Authors:**	Ignacio Garach Vélez<br>Francisco Manuel Ortuño Guzmán<br>Ignacio Rojas Ruiz<br>Luis Javier Herrera Maldonado
**Order of Authors Secondary Information:**	
**Response to Reviewers:**	Response to editor:

Powered by Editorial Manager® and ProduXion Manager® from Aries Systems Corporation

In addition, please register 16SMicrobiomeMLFS in the bio.tools and SciCrunch.org databases to receive RRID (Research Resource Identification Initiative ID) and biotoolsID identifiers, and include these in your manuscript. Computational workflows should be registered in workflowhub.eu and the DOIs cited in the relevant places in the manuscript. These will facilitate tracking, reproducibility and re-use of your tool.

We thank the editor's feedback. Code and workflow has been submited to these platforms and is cited at the "Availability of source code and requirements" section.

Response to the reviewers (Latex Formated Document submited with these answer to enable easier review).

We thank the reviewers for their constructive comments. We have addressed all of them and modified the paper accordingly. Our detailed answers follow.
Please note that reviewers' comments are in bold while our answers are not. Additions to the original manuscript are indicated in blue at the revised manuscript.

Reviewer 1

1) Are the methods appropriate to the aims of the study, are they well described, and are necessary controls included? Yes, the study aims to compare biomarker discovery in 16S microbiome disease classification. They particularly look at various normalization and feature selection processes across different classification algorithms. They include controls of testing the models without normalization as a baseline.
2) Are the conclusions adequately supported by the data shown? Yes, the data is clearly and professionally presented in the paper along with the author's conclusions on how future work should approach selecting the appropritate normalization, feature selection, or even algorithm for classification.
3) Please indicate the quality of language in the manuscript. Does it require a heavy editing for language and clarity? The manuscript is of high quality. The sections are clear, appropriate data, tests, and analysis are provided. There are little if any language errors. My small suggestion would be to add numbers to the section and subsection seperators.
4) Are you able to assess all statistics in the manuscript, including the appropriateness of statistical tests used? Yes, again the data is clearly and professionally presented.

Reply: We thank the reviewer for their positive and constructive feedback. We agree that numbering the sections and subsections could improve navigability and clarity. However, we have followed the official Overleaf template provided for this journal, and upon reviewing published articles, we noticed that section numbering is generally not used. Nevertheless, we extend this recommendation to the editor and are happy to implement section numbering if deemed appropriate.

Reviewer Point P1.1 — My number one issue with the paper is it would be significantly better if the authors provided more novelty. As it is, the paper needs benchmarking with more existing methods.

Reply: We thank the reviewer for raising this important point regarding the novelty and methodological breadth of our study. In response, we have expanded the benchmarking to include additional normalization techniques—specifically, log-ratio abundance (logRA) and presence/absence (PA)—in combination with all classifiers and feature selection methods. We also incorporated the consideration of rarefaction prior to normalization for baseline classification, further enriching the comparative analysis.
Furthermore, we included Random Forest Feature Importances as an additional feature selection method, allowing for the evaluation of a different feature selection approach and its interactions within the different steps of our pipeline.

The results of the new methods included in the benchmarking are presented on pages 3 to 6.
Moreover, they are discussed mainly on page 9.

Powered by Editorial Manager® and ProduXion Manager® from Aries Systems Corporation

We fully agree with the reviewer that there is potential for incorporating more complex methodologies (such as wrapper-based feature selection or optimization techniques using evolutionary algorithms).
In fact, we explicitly acknowledge this in the potential implications section, where we highlight such approaches as promising future directions. However, in the present work, we intentionally focused on a set of methods that are widely used, computationally tractable, and amenable to extensive validation across a large number of datasets. Our goal was to provide a comprehensive and reproducible comparison across normalization, feature selection, and classification steps, laying the groundwork for future
investigations involving more advanced and computationally intensive techniques.
We believe that the additions made in this revised version considerably enhance the novelty and completeness of our study, and we appreciate the reviewer's suggestion to further strengthen its contribution.

Reviewer 2

This manuscript addresses an important and timely topic concerning the impact of data transformation and feature selection techniques on machine learning classification of microbiome datasets. Given the growing interest in developing robust predictive models in microbiome research, especially for disease classification, this study is of high relevance. The paper is generally well-written and provides a comprehensive overview of commonly used preprocessing and modeling methods in the microbiome field. However, the manuscript would benefit from several substantial revisions to improve clarity, interpretability, and completeness:

Reply: Thank you for your constructive and helpful feedback on our manuscript. We appreciate the time and effort you have dedicated to providing such a thorough review. We have carefully considered all the points raised and have made substantial revisions. Below, we provide a point-by-point response to each of your comments, detailing the changes we have implemented.

Reviewer Point P2.1 — Inclusion of Additional Transformations: The manuscript should include results using log-relative abundance (log-RelAb) and presence/absence (PA) transformations, as these are standard approaches in microbiome data preprocessing and their inclusion would improve the comprehensiveness of the analysis. Additionally, it would be valuable to evaluate the effect of rarefaction on model performance. Including rarefied datasets would allow the authors to assess how this commonly used normalization method influences classification outcomes and whether it interacts with transformation or feature selection strategies.

Reply: We thank the reviewer for this excellent and constructive suggestion. In response, we expanded our study to include two additional normalization strategies: log-relative abundance and presence/absence (PA). The log-relative abundance transformation is now referred to as logRA throughout the manuscript for consistency. Both transformations were incorporated into the baseline classification analysis as well as the full set of pipeline comparisons across all feature selection methods and classifiers. The inclusion of the presence/absence (PA) transformation, in particular, provided valuable insight and contributed significantly to our comprehension of the nature of the problem and to the completeness of our analysis. We are especially grateful for this recommendation, which led to meaningful improvements in the overall quality and depth of the study. Results of baseline classification with the additional proposed normalizations are presented on pages 3 to 5.

LogRA and PA results sections read as:

"Finally, focusing on data normalized using the remaining two transformations, log-transformed Relative Abundances (logRA) and Presence-Absence (PA), the Friedman test also revealed significant differences between classifiers in both cases . For the logRA normalization, results were extremely similar to those from CLR. Logistic Regression models achieved the best average ranks, followed by Support Vector Machines and Boosting. Unlike what was observed with CLR normalization, in this case Boosting

models did not show statistically significant differences in performance compared to Logistic Regression and SVM. Random Forest and KNN fell behind, being significantly outperformed by the top models. In contrast with abundance-based transformations, the PA normalization showed higher performance in a larger range of different models, with Logistic Regression, SVM and Random Forest ranking high and with no significative statistical differences between them. However, the Boosting and KNN results
were not comparable to the top ones, even though KNN worked better compared with the other normalizations.
On average, best performing models were Random Forest for RA data and Logistic Regression for CLR and logRA normalizations. PA obtained similar performance to the best abundance-based pipelines when using Random Forest or Logistic Regression classifier (see again AUC boxplots at figure 2 and Finner's test results at figure 3)"

We thank the reviewer for the suggestion on assessing rarefaction. In response, we conducted an additional analysis to assess the impact of rarefaction on classification performance across normalization methods. Two rarefaction strategies were evaluated: (i) standard rarefaction to the minimum library size, and (ii) a modified approach using the 5th percentile of sequencing depths to mitigate information loss from very shallow samples. These results are now included at the end of the Baseline classification section with tests and figures in the Supplementary material. Results showed that rarefaction consistently did not provide performance benefits and, in some cases, led to substantial degradation in accuracy. As such, rarefaction was not applied in subsequent pipeline
analyses involving feature selection. Rarefaction analysis section is located at the end of page 4 of the manuscript and reads as:

"An additional analysis was conducted to evaluate the impact of rarefaction and its interactions with normalization methods on classification performance. Rarefaction involves subsampling the count data to a uniform sequencing depth across samples. This is typically done using the minimum library size observed in the dataset. However, when certain samples are extremely shallow, this approach can result in a substantial loss of information. To overcome this, we included a second variant where the rarefaction depth was set to the 5th percentile of each dataset sample depths, removing samples with less reads than this quantity. In the global comparison, Wilcoxon paired tests revealed statistically significant differences between non-rarefied data and both rarefaction approaches, with all p-values indicating strong significance in detriment of rarefaction (specifically, $p = 4.4 \cdot 10^{-13}$ for standard rarefaction and $p = 9.3 \cdot 10^{-5}$ for Q05 rarefaction). Quantile-modified rarefaction also performed significantly better than standard rarefaction $p = 1.5 \cdot 10^{-9}$.
When examining each normalization method individually, consistent patterns can be observed (see Supplementary Figure S1). For RA and CLR normalization, non-rarefied data significantly outperformed both rarefied counterparts. In contrast, no differences were found for logRA and PA normalization for quantile-based rarefaction, though it still did not yield any improvements. Interestingly, for CLR, logRA, and PA, the quantile-based rarefaction yielded significantly better results than standard rarefaction (see Supplementary Table T2). Therefore, rarefaction was not applied in the subsequent pipeline analyses involving feature selection, as it consistently led to either inferior or non-improved results across normalization methods."

We also mentioned them at the global discussion on page 9 and rigorously modified the sections Background and Methods to include the new techniques considered.

Reviewer Point P2.2 — Simplify Figure 1 for Clarity: Figure 1 would be more effective if it presented a single boxplot per transformation method (e.g., CLR, RELAB) to facilitate simpler and more direct comparisons.

Reply: We have followed the reviewer's suggestion and created a new figure showing a single boxplot for each transformation method to facilitate a straightforward comparison. We have incorporated this as a key part of a revised and consolidated Figure 2 (Figure 1 in the first version of the MS). To provide a complete picture that supports the detailed analysis in the text, this new figure now also includes the breakdown per normalization method integrated into a single, cohesive panel. It can be seen now at the

Powered by Editorial Manager® and ProduXion Manager® from Aries Systems Corporation

bottom of page 3. We believe this revised figure successfully addresses your concern for simplicity while retaining the necessary detail discussed throughout the manuscript.

Reviewer Point P2.3 — Improve Table 8 Presentation: Table 8 lacks sufficient description. Consider either improving the accompanying explanation or replacing it with a figure (e.g., boxplots) to visually highlight performance distributions. If a table is retained, bold the best-performing results for clarity.

Reply: We appreciate the reviewer’s valuable feedback regarding the presentation of Table 8 (now Table 7). We have thoroughly revised the table to enhance its clarity and informativeness, addressing all your suggestions.
We have significantly improved the accompanying description to provide a more comprehensive explanation of its content. Furthermore, we have restructured the table to clearly delineate the different aspects of the results. On the left side, we now present the baseline classification performance using all features. This is followed by the performance of the top-performing feature selection methods, each explicitly paired with its corresponding percentage of feature reduction. As suggested, the bestperforming results for each dataset are now clearly bolded, making it much easier for the reader to identify optimal outcomes. It can be seen now at the top of page 10.

Reviewer Point P2.4 — Unify Boxplots in Figure 5: For better comparison, combine all boxplots in Figure 5 into a single panel and ensure the Y-axis scale is consistent across plots.

Reply: We thank the reviewer for this helpful suggestion. In the revised manuscript, we have addressed the Y-axis consistency issue and updated the figure accordingly. The figure referenced is now Figure 4. While we understand the value of combining all boxplots into a single panel for comparison, we decided to maintain separate panels to clearly distinguish between comparisons and avoid visual clutter. It can be seen now at the bottom of page 4.

Reviewer Point P2.5 — Format P-values Appropriately: Round p-values to a reasonable number of decimal places (e.g., three significant digits) for readability.

Reply: We thank the reviewer for this observation. In our manuscript, we have now formatted pvalues consistently using scientific notation or fixed-point notation with two significant figures, which we believe offers a balance between readability and precision. For very small p-values, we intentionally used scientific notation to ensure that their order of magnitude remains visible, which is important for displaying the strength of statistical evidence. This formatting choice is in line with other studies in the same field. Nevertheless, if the reviewer or editor considers a different notation more appropriate, we would be happy to adapt accordingly.

Reviewer Point P2.6 — Enhance Figure Quality: Improve the resolution of all figures and ensure consistent text formatting across all graphical elements.

Reply: We thank the reviewer for this suggestion. We have addressed this point by combining some of the figures for clarity and overall improving the resolution of all figures. We have also ensured consistent text formatting across all graphical elements. We believe these changes enhance the visual quality and readability of the figures now numbered as 2, 3, 4, 7, 8, 9, 11 and 12.

Reviewer Point P2.7 — Revise Figure 9 Visualization: Instead of displaying a small plot, use a line plot with a shaded area representing standard deviation or confidence intervals to better convey variability.

Reply: We thank the reviewer for the helpful suggestion. In response, we have revised Figure 9 by replacing the previous corner plot with a line plot that includes shaded areas representing the standard deviation, thus providing a clearer visualization of variability across the data. We have also enhanced the overall quality of the figure. It can be seen now at the bottom of page 7.

Reviewer Point P2.8 — Terminology Refinement: Avoid using the term ”biomarker” unless biologically validated. ”Most important features” or ”predictive features” would

be more appropriate in the context of machine learning feature selection.

Reply: We thank the reviewer for the valuable feedback regarding the use of the term ”biomarker”. We agree and we have implemented the suggested changes throughout the manuscript. Specifically, we have replaced ”biomarker” with more appropriate terms such as ”predictive features,” ”microbial features,”, ”key attributes” and ”discriminative features” in the context of our machine learning feature selection. We have only retained the term ”biomarker” in general sections, such as the Introduction, Methods, Potential Implications and Conclusions, where we discuss the broader state of the art and the potential for selected features to be biologically or externally validated as true biomarkers in future research. This ensures that our terminology remains precise and contextually accurate.

Reviewer Point P2.9 — Relocate Table 7 Content: The discussion of specific taxa and Table 7 should be moved into the Results section, as it presents findings rather than interpretation.

Reply: We thank the reviewer observation. We have moved the content of Table 7 into the Analyses section (now table 5 at the top of page 8), where we now present the selected taxa and highlight the high genera variability observed in the results. However, we kept the interpretation and comparison with previously reported associations in the literature in the Discussion section, as we believe this context helps clarify the relevance of the findings.

Reviewer Point P2.10 — Reorganize the Discussion Section: A significant portion of the current discussion reads as results. These subsections should be moved to the Results section, and the Discussion should be condensed to focus on interpretation, implications, and broader context.

Reply: We appreciate the reviewer’s observation. In response, we have reorganized and condensed the Discussion section to avoid repeating points already presented in the Analyses section. We now focus more on interpreting the findings, exploring possible reasons behind the observed behavior of the methods, and situating our results within the context of relevant literature. These changes aim to enhance the clarity of the discussion.

Reviewer Point P2.11 — Add a Study Design Schematic: A clear, concise diagram summarizing the study design, including data processing, transformation, modeling, and evaluation steps, would greatly enhance reader comprehension.

Reply: We thank the reviewer for the suggestion. We have incorporated a graphical abstract into our submission, which illustrates the key steps of our study design, including data processing, normalization, feature selection, classification, and evaluation. We believe this addition significantly enhances the clarity and comprehension for the reader.

Reviewer Point P2.12 — Summarize Feature Importance Methods: Consider summarizing the feature importance techniques in a dedicated table to facilitate comparison and improve readability.

Reply: We thank the reviewer for this suggestion. We have incorporated a dedicated table (Table 8 at the top of page 12) summarizing the feature importance techniques to provide readers with a quick yet informative overview of the methods.

Reviewer Point P2.13 — Expand Method Descriptions: Briefly introduce each classification method (e.g., logistic regression, random forest, SVM) in the main text and provide a more detailed description in the Methods section.

Reply: We appreciate rhe reviewer’s valuable comment. We have now addressed this by briefly introducing the classifiers in the main text, as suggested.

”These models represent a diverse set of classification strategies. Random Forest and XGBoost are tree-based ensemble methods known for their robustness and ability to capture complex non-linear relationships. Logistic Regression is a linear model that

Powered by Editorial Manager® and ProduXion Manager® from Aries Systems Corporation

	provides interpretable coefficients and performs well when the data is linearly separable. Support Vector Machine is a margin-based classifier effective in high-dimensional spaces, particularly suitable for sparse data. Finally, K-Nearest Neighbor is a lazy learning method that classifies samples based on similarity to their neighbors, though it can be sensitive to noise and sparsity in the data. A more detailed description of each classifier is provided in the Methods section."<br><br>Furthermore, we have dedicated subsections within the Methods section to provide a more in-depth description of each classifier, ensuring a comprehensive understanding for the reader.
**Additional Information:**	
**Question**	**Response**
Are you submitting this manuscript to a special series or article collection?	No
**Experimental design and statistics**<br><br>Full details of the experimental design and statistical methods used should be given in the Methods section, as detailed in our Minimum Standards Reporting Checklist. Information essential to interpreting the data presented should be made available in the figure legends.<br><br>Have you included all the information requested in your manuscript?	Yes
**Resources**<br><br>A description of all resources used, including antibodies, cell lines, animals and software tools, with enough information to allow them to be uniquely identified, should be included in the Methods section. Authors are strongly encouraged to cite Research Resource Identifiers (RRIDs) for antibodies, model organisms and tools, where possible.<br><br>Have you included the information requested as detailed in our Minimum Standards Reporting Checklist?	Yes
**Availability of data and materials**<br><br>All datasets and code on which the	Yes

conclusions of the paper rely must be either included in your submission or deposited in publicly available repositories (where available and ethically appropriate), referencing such data using a unique identifier in the references and in the “Availability of Data and Materials” section of your manuscript.<br><br>Have you have met the above requirement as detailed in our Minimum Standards Reporting Checklist?	
GigaScience has policies and guidelines in place for the use of generative AI-writing tools such as ChatGPT. If you have used such writing tools to assist with writing the manuscript this must be declared and cited in the text. Authors should not list AI-writing tools and other AI-assisted technologies as an author or co-author and should acknowledge that they are fully responsible for text generated or refined by AI-writing tools.<p><br><br>A summary of use (particularly in the introduction or among methods) needs to be included at the end of the paper, and the outputs should also be included as a supplementary file hosted in GigaDB or other open repositories. Please read our guidelines for more information. <p><br><br>By submitting to GigaScience, you are aware of the journal's AI-writing tools policy, and if you have declared use of such tools below, you have acknowledged this where appropriate in your manuscript and have made a summary of use and outputs available. <p><br><b>AI-assisted writing tools have been used in the preparation of this manuscript?	Yes

Powered by Editorial Manager® and ProduXion Manager® from Aries Systems Corporation

Revised Manuscript

Click here to access/download;Manuscript;Revised Manuscript.pdf

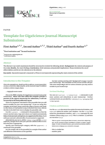

GigaScience, 2025, 1–16

doi: xx.xxxx/xxxx
Manuscript in Preparation
RESEARCH

RESEARCH

Exploring the role of normalization and feature selection in microbiome disease classification pipelines

Ignacio Garach Vélez[1*], Francisco Manuel Ortuño Guzmán[1], Ignacio Rojas Ruiz[1] and Luis Javier Herrera Maldonado[1]

[1]Computer Engineering, Automatics and Robotics Department, University of Granada, 18071 Granada, Spain;

[*] Correspondence address: igarachv@ugr.es

Abstract

Background: Disease classification using 16S rRNA microbiome data faces challenges of high dimensionality, compositionality, and sparsity, compounded by the inherent small sample sizes in many studies. Machine learning and feature selection techniques offer potential to identify robust biomarkers and improve classification performance; however, their comparative effectiveness across diverse methods and datasets has been insufficiently explored. This study evaluates multiple feature selection techniques alongside normalization strategies, focusing on their interplay with classifier performance. **Results**: Our analyses revealed that Centered Log-Ratio normalization improves the performance of Logistic Regression and Support Vector Machine models and facilitates feature selection, whereas Random Forest models yielded strong results using relative abundances. Interestingly, Presence-Absence normalization was able to achieve similar performance compared to abundance-based transformations across classifiers. Among feature selection methods, minimum redundancy maximum relevancy (mRMR) surpassed most methods in identifying compact feature sets and demonstrated performance comparable to LASSO, which obtained top results requiring lower computation times. Autoencoders needed larger latent spaces to perform well and lacked interpretability, Mutual Information suffered from redundancy, and ReliefF struggled with data sparsity. **Conclusions**: Overall, feature selection pipelines improved model focus and robustness via a massive reduction of the feature space. mRMR and LASSO emerged as the most effective methods across datasets.

Key words: microbiome; normalization; machine learning; feature selection; classification;

Background

Microbial communities are present in almost every part of the human body, from the mouth to the gut, skin, and reproductive tract. The gut microbiome, widely recognized as the most extensively studied microbial community, has attracted significant attention in recent years. Numerous studies have linked microbiota dysbiosis to the development of different health problems [1]. Not only infectious diseases but also cancer [2], inflammatory disorders such as Crohn's disease [3] or arthritis [4], and even neurodegenerative disorders such as Parkinson due to the gut-brain axis [5].

Although numerous studies employing classical statistical methods have been conducted to demonstrate these connections, the rapid advancement of next-generation sequencing technologies and the availability of increasingly larger, higher-quality datasets with more diverse cohorts underscore the potential of the use of machine learning techniques in these studies. These new approaches offer opportunities to uncover complex patterns and associations that might be challenging to detect using traditional statistics, potentially leading to more refined insights and robust predictive models.

The task of detecting the presence of a disease in a host from the gut microbiome has been approached in various ways [1]. Machine learning methods, and more specifically the supervised learning paradigm, enable researchers to address this disease classification problem from microbiome data with optimal prediction accuracy, helping to identify key biomarkers and therapeutic targets [6].

16S rRNA microbiome data presents several inherent challenges that complicate its application in disease classification tasks. Firstly, the high dimensionality of these datasets, with hundreds or thou-

Compiled on: July 13, 2025.
Draft manuscript prepared by the author.

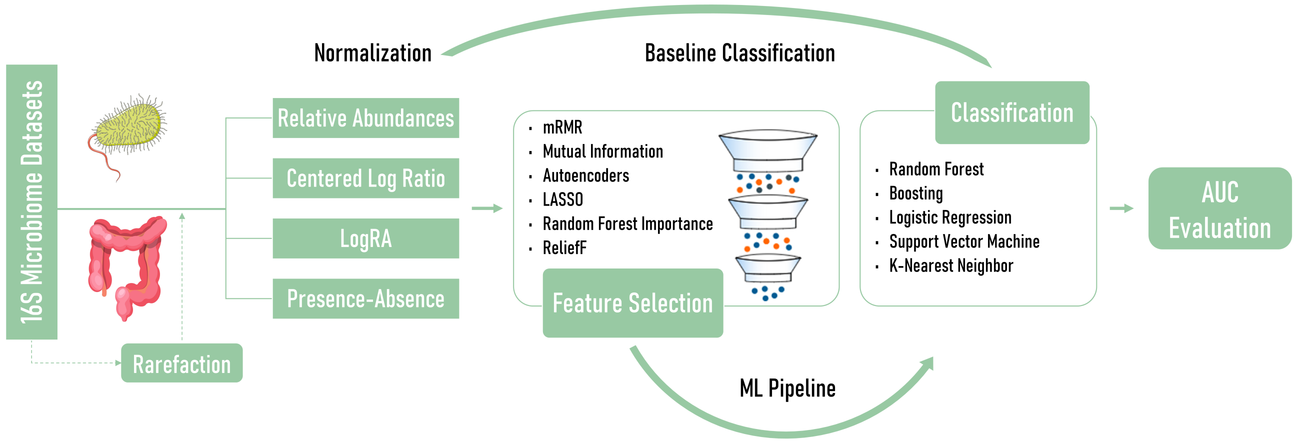

Figure 1. Graphical Abstract. Overview of the proposed pipeline, illustrating the normalization strategies, feature selection techniques, and classification algorithms evaluated.

sands of features (Operational Taxonomic Units -OTUS- or Amplicon Sequence Variants -ASVs-) representing bacterial species, often greatly exceeds the number of available samples, leading to the classic curse of dimensionality problem [7]. Additionally, the sparse nature of this data [8], where most taxa have very low or absent abundances in many samples, increases the chance of overfitting and risks model generalization. Moreover, microbiome data is compositional [9], meaning the abundances of taxa are proportions that sum up to one (or any read number if not under closure operation). This fact introduces important dependencies between features that can harm Machine Learning (ML) algorithms and must be properly accounted for, in order to avoid misleading conclusions. These challenges emphasize the need for normalization and advanced techniques that respect the unique characteristics of microbiome data [10]. In this research, we compare the performance of models using relative abundances and various normalization techniques, and also evaluate the effect of applying rarefaction of counts prior to normalization.

Moreover, feature selection arises as a great tool for microbiome data analysis challenges, allowing to find small taxa signatures and helping classifiers to deal with the high-dimensional compositional-sparse data, ideally improving performance. In this article, our aim is to evaluate multiple feature selection methods and their interaction with classifiers to identify robust microbial features across diseases using a large collection of 16 rRNA disease classification datasets. Feature selection has been used to address this problem with some cohorts [11, 12], but a systematic comparison in multiple datasets as the one presented here is lacking. This offers a broader understanding of feature selection and model interactions in the field of 16S microbiome data modeling. A summary of the analysis pipeline and key methodological components is provided in Figure 1 as a graphical abstract.

Data Description

Although some big initiatives have been developed to centralize microbiome data studies such as The Human Microbiome Project [13] or The American Gut Project [14], big curated repositories with metadata enabling further study of disease classification problems are lacking. To compare the performance of our pipelines we leveraged 16S gut datasets from MicrobiomeHD, a standardized database of human gut 16S microbiome case-control studies and their associated patient metadata,[15] and MLrepo, a repository of curated microbiome-related supervised learning tasks, [16]. We selected datasets containing at least 75 samples, and a minimum 1:6 imbalance ratio between cases and controls. To enhance the benchmark, we retrieved 2 more gut datasets online with abundance tables and metadata available [17]. Multiple datasets from MicrobiomeHD that exhibited extremely low performance in preliminary tests (an AUC lower than 0.60) were excluded from the final analysis to avoid biases in the interpretation of the results. Similarly, datasets with excessively high performance (AUC greater than 0.95) were also excluded, as their classification tasks were too simple, offering limited variability and failing to provide meaningful differences between algorithms. The final selected datasets and their respective original studies are available at Table 1. A total of 3320 gut samples from 15 datasets were considered.

Table 1. Benchmark datasets used in our classification analysis, including their imbalance ratios (IR) and references.

Dataset	Samples	Features	IR	Reference
ART	114 (86, 28)	10 733	3.07	[18]
CDI	336 (93, 243)	3456	2.61	[19]
CRC1	490 (229, 261)	6920	1.14	[20]
CRC2	102 (46, 56)	837	1.22	[21]
HIV	350 (293, 57)	14 425	5.14	[22]
CD1	140 (78, 62)	3547	1.26	[23]
CD2	160 (68, 92)	3547	1.35	[23]
IBD1	91 (67, 24)	2742	2.79	[24]
IBD2	114 (68, 46)	1496	1.48	[25]
CIR	77 (51, 26)	3104	1.96	[26]
MHE	77 (26, 51)	3104	1.96	[26]
OB	281 (220, 61)	6386	3.61	[27]
PAR1	148 (74, 74)	10 232	1.00	[28]
PAR2	333 (201, 132)	6844	1.52	[29]
PAR3	507 (323, 184)	12 198	1.76	[30]

ART: Arthritis; CDI: Clostridium difficile Infection; CRC1 and CRC2: Colorectal Cancer; HIV: Human Immunodeficiency Virus; CD1 and CD2: Crohn's Disease; IBD1 and IBD2: Inflammatory Bowel Disease; CIR: Cirrhosis; MHE: Minimal Hepatic Encephalopathy; OB: Obesity; PAR1, PAR2, and PAR3: Parkinson's Disease.
CD1 and CD2 were taken from MLRepo, PAR2 and PAR3 were retrieved from their respective reference, and the remaining datasets were obtained from MicrobiomeHD.

Analyses

Empirical evidence is presented on the performance levels of baseline machine learning (ML) models across 16S microbiome datasets, focusing on the impact of normalization techniques and feature selection methods. Validation AUC, derived from a nested cross-validation procedure, is used as the primary metric to assess model performance. The models are implemented using the scikit-learn library [31], ensuring consistency and reproducibility. Hyperparameter tuning is done at the inner loop of the validation. The parameter combinations considered are available at Table 2.

Table 2. Hyperparameter grid for classifiers used in the study.

Classifier	Hyperparameter	Parameters
Random Forest	n_estimators<br>max_features<br>max_depth	[200, 300, 400]<br>[sqrt, log2]<br>[None, 3, 5, 7, 8]
KNN	n_neighbors<br>weights	[7, 9, 11, 13, 15, 17, 19, 21]<br>[uniform, distance]
SVM	C<br><br>kernel<br>gamma	[0.001, 0.1, 1, 10, 100, 1000]<br>['rbf']<br>[scale, auto]
Logistic Regression	C	np.logspace(-4, 4, 20)
Boosting	max_depth<br>n_estimators	[3, 5, 7, 8]<br>[300, 500, 800]

Baseline classification and influence of normalization

To perform an initial assessment of the predictive power of microbiome features for the different diseases under different normalization strategies, we trained and validated 5 ML models (Random Forest (RF), XGBoost (XGB), Logistic Regression (LR), Support Vector Machine (SVM) and K-Nearest Neighbor (KNN)) on each dataset. We used relative abundance data and then compared the results with the same models applied to data transformed using three different normalization techniques, namely Centered Log-Ratio (CLR), log-transformed RA (logRA), and Presence-Absence (PA) (see Methods section for details). Rarefaction was also assessed prior to normalization to evaluate its effect on performance.

These models represent a diverse set of classification strategies. Random Forest and XGBoost are tree-based ensemble methods known for their robustness and ability to capture complex nonlinear relationships. Logistic Regression is a linear model that provides interpretable coefficients and performs well when the data is linearly separable. Support Vector Machine is a margin-based classifier effective in high-dimensional spaces, particularly suitable for sparse data. Finally, K-Nearest Neighbor is a lazy learning method that classifies samples based on similarity to their neighbors, though it can be sensitive to noise and sparsity in the data. A more detailed description of each classifier is provided in the Methods section.

On our initial analysis, we compared the overall performance of the four normalization strategies. As Figure 2A displays, the distribution of AUC scores is similar across these methods. This was statistically confirmed by a global Friedman test, which yielded a non-significant p_{val} = 0.56.

Then, to explore the results more in depth, we proceededed with a more granular analysis, breaking down the performance by classifier for each normalization method individually to uncover more specific trends (see Figure 2B).

Focusing on the RA approach, trends on classifier performance showed that tree-based models obtained best results across most datasets with Random Forest yielding the best AUC on average. Boosting (XGBoost) obtained the best performance at CDI dataset with an AUC of 0.926. Friedman's multiple comparisons test revealed statistical differences across algorithms and Finner's posthoc results shown at figure 3 confirmed this ranking, with Random

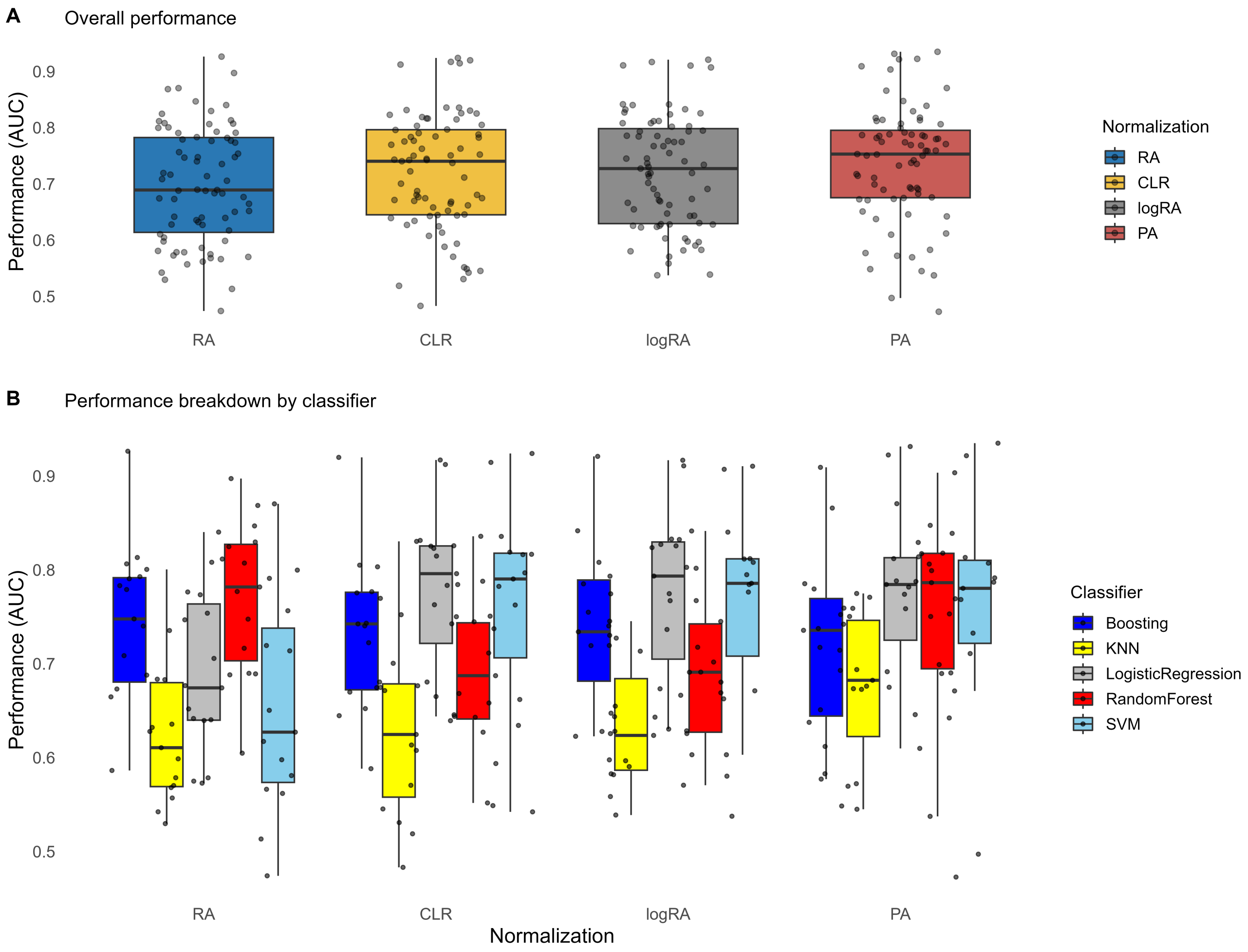

Figure 2. Overall normalization performance and breakdown by classifier for the four types of normalizations -baseline classification–.

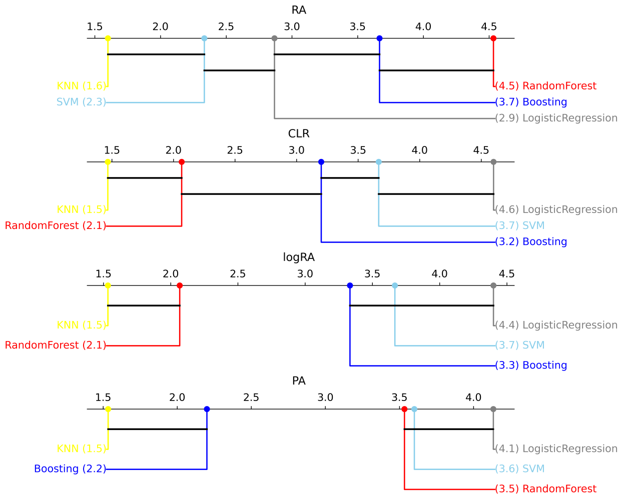

Figure 3. Finner's test results critical distance diagrams for each normalization.

Forest results significantly different when compared with all algorithms except for Boosting. K-Nearest Neighbors obtained the worst results, while not being statistically different from Support Vector Machine. Sitting in the middle ground, Logistic Regression obtained marginally better performance than SVM and slightly worse than Boosting.

Next, focusing on data normalized using the CLR transformation, trends clearly changed with Friedman's test again showing significant differences in performance. In this case, best models were Logistic Regression and Support Vector Machines. Boosting stays one step below as a stable alternative and Random Forest results dramatically fall into the fourth ranking position. One notable example of this drop in performance with normalization is the Obesity dataset which obtained an AUC of 0.869 for relative abundances and 0.788 for CLR normalization (note that Logistic Regression and SVM do improve RA results for this dataset).

Finally, focusing on data normalized using the remaining two transformations, log-transformed Relative Abundances (logRA) and Presence-Absence (PA), the Friedman test also revealed significant differences between classifiers in both cases . For the logRA normalization, results were extremely similar to those from CLR. Logistic Regression models achieved the best average ranks, followed by Support Vector Machines and Boosting. Unlike what was observed with CLR normalization, in this case Boosting models did not show statistically significant differences in performance compared to Logistic Regression and SVM. Random Forest and KNN fell behind, being significantly outperformed by the top models.

In contrast with abundance-based transformations, the PA normalization showed higher performance in a larger range of different models, with Logistic Regression, SVM and Random Forest ranking high and with no significative statistical differences between them. However, the Boosting and KNN results were not comparable to the top ones, even though KNN worked better compared with the other normalizations.

On average, best performing models were Random Forest for RA data and Logistic Regression for CLR and logRA normalizations. PA obtained similar performance to the best abundance-based normalization pipelines when using Random Forest or Logistic Regression classifier (see again AUC boxplots at figure 2 and Finner's test results at figure 3). AUC values varied between datasets showing which ones are more challenging (see Supplementary Tables T3-T6), varying from the CDI dataset with AUC of 0.897 with Random Forest (with RA) and 0.917 with Logistic Regression (with CLR), to arthritis ART with an AUC of 0.605 with Random Forest (with RA) and 0.644 with Logistic Regression (with CLR).

We finally carried out a comparison between best models with and without normalization using Friedman ranks test. As figure 4 shows, significant differences were found between normalizations. It can be observed that Logistic Regression comparison on both normalization methods revealed superior performance with CLR and logRA (B) and the opposite trend in the Random Forest comparison, with RA and PA obtaining better results (A). The PA results were robust and comparable to the best normalizations in both comparisons.

To further investigate, we compared the best performing models for each normalization RA + Random Forest, CLR + Logistic Regression, logRA + Logistic Regression and PA + Random Forest. Friedman's test did not reveal differences $p_{val} = 0.34$ (C), suggesting different ways classifiers can be adapted to deal with compositional data.

An additional analysis was conducted to evaluate the impact of rarefaction and its interactions with normalization methods on classification performance. Rarefaction involves subsampling the count data to a uniform sequencing depth across samples. This is typically done using the minimum library size observed in the dataset. However, when certain samples are extremely shallow, this approach can result in a substantial loss of information. To overcome this, we included a second variant where the rarefaction depth was set to the 5th percentile of each dataset sample depths,

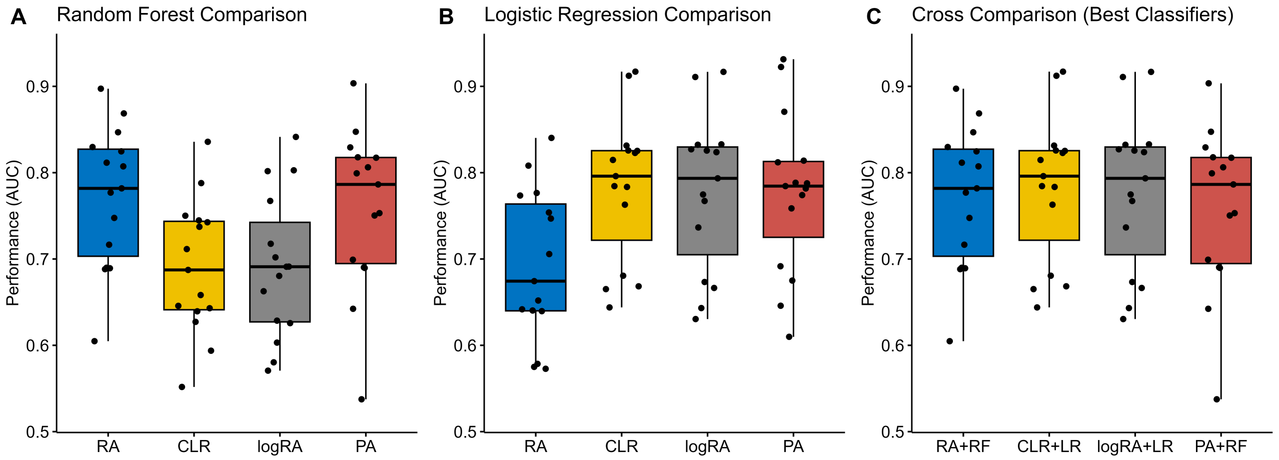

Figure 4. A. Random Forest comparison through normalization methods. Friedman's test with p-value = $1.1 \cdot 10^{-5}$. **B.** Logistic Regression comparison through normalization methods. Friedman's test with p-value = $1.3 \cdot 10^{-4}$ **C.** Comparing best performing classifiers for each normalization. Friedman's test with p-value = 0.34

removing samples with less reads than this quantity.

In the global comparison, Wilcoxon paired tests revealed statistically significant differences between non-rarefied data and both rarefaction approaches, with all p-values indicating strong significance in detriment of rarefaction (specifically, $p = 4.4 \cdot 10^{-13}$ for standard rarefaction and $p = 9.3 \cdot 10^{-5}$ for Q05 rarefaction). Quantile-modified rarefaction also performed significantly better than standard rarefaction $p = 1.5 \cdot 10^{-9}$.

When examining each normalization method individually, consistent patterns can be observed (see Supplementary Figure S1). For RA and CLR normalization, non-rarefied data significantly outperformed both rarefied counterparts. In contrast, no differences were found for logRA and PA normalization for quantile-based rarefaction, though it still did not yield any improvements. Interestingly, for CLR, logRA, and PA, the quantile-based rarefaction yielded significantly better results than standard rarefaction (see Supplementary Table T2).

Therefore, rarefaction was not applied in the subsequent pipeline analyses involving feature selection, as it consistently led to either inferior or non-improved results across normalization methods.

Feature Selection Performance Comparison

We also examined the predictive power of the feature set rankings provided by the different ML feature selection techniques. We considered Mutual Information, mRMR, LASSO, ReliefF, Random Forest importances and autoencoders (see Methods section).

Table 3 shows top 5 performing Feature Selection + Classifier pipelines per normalization method, illustrating that the ones involving Logistic Regression outperformed the other methods. Thus, we observe that trends in classifier performance with CLR and logRA normalizations persisted after feature selection, with Logistic Regression as the best performing method across datasets. Furthermore, Random Forest continue to be the best classifier for RA data even after feature selection. Additionally, PA stability across classifiers is also maintained, yielding strong results using both LR and RF as FS pipeline classifiers.

In our search for the best ML pipeline we executed all combinations of normalization techniques with the selected feature selection methods and classifiers. A total of 900 models were validated for each normalization method, and to observe effects of normalization on feature selection, we performed Friedman's test, which showed significant differences between groups $p = 1.9 \cdot 10^{76}$. A boxplot showing the results across normalizations is shown in Figure 5. As it can be observed, CLR normalization outperformed all other methods overall. Moreover, pipelines relying on relative abundance data consistently underperformed in comparison to all normalization-based approaches. These findings are further supported by the Finner post-hoc test results shown in Figure 6, where logRA and PA occupy an intermediate position, being the only pair of normalization methods with no statistically significant differences between them.

Similarly, to select the most suitable classifier, we performed multiple comparison tests. The results, detailed in the Supplementary Figure S2, revealed that Logistic Regression was statistically

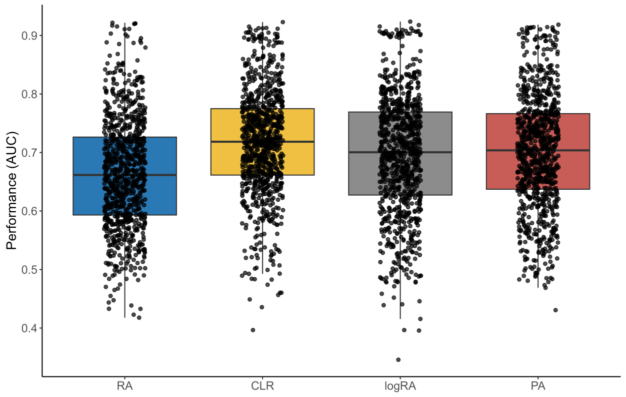

Figure 5. Boxplot of all posible Feature Selection + Classifier pipelines for each normalization technique

superior to the other evaluated models. The previously presented table 3, which highlighted the top-performing pipelines for the CLR normalization, further corroborates this choice, with LR consistently appearing among the best models.

Relying on these results, we selected CLR + Logistic Regression as the reference to compare the feature selection algorithms on the normalized data, simplifying the analysis and enabling a more focused evaluation of their effectiveness.

Figure 7 and Table 4 show results of nested validation from this analysis. As expected, Friedman's multiple comparisons test revealed statistical differences with high significance $p_{val} = 8.6 \cdot 10^{-7}$. Two groups of methods are revealed in terms of results. The first group includes three algorithms with good performance at a comparable level: LASSO, mRMR50 and mRMR100, which consistently yield stronger results across datasets. On the other hand, the second group comprises seven algorithms with lower mean performance, including ReliefF50, ReliefF100, variational autoencoder, MIFS50, MIFS100, RF50 and AE50 with the last one a step above the others. Finally, AE100 and RF100 performances place them in a middle spot between the groups. Critical distance diagram at Figure 8 illustrates how Finner's posthoc test confirms these groups.

The two variants of the minimum redundancy maximum relevance algorithm (50 and 100) achieved the highest AUC scores on a big number of datasets. Small differences were found when varying the number of selected features, indicating that fewer than 50 features may be sufficient for some datasets. Finner's test showed significant differences between them and their MIFS counterparts.

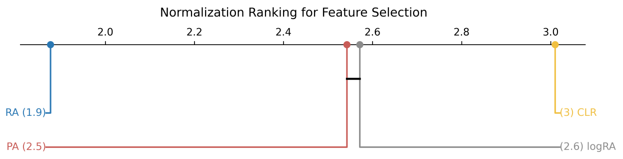

Figure 6. Critical difference diagram from Finner's Post-hoc test on feature selection results for each normalization.

Table 3. Top 5 machine learning pipelines (Classifier + Feature Selection) sorted by mean AUC across datasets for each normalization method.

RA		CLR		logRA		PA	
**Pipeline**	**Mean AUC**	**Pipeline**	**Mean AUC**	**Pipeline**	**Mean AUC**	**Pipeline**	**Mean AUC**
RF + RF100	0.761	LR + mRMR100	0.772	LR + mRMR100	0.766	RF + mRMR100	0.757
RF + mRMR100	0.757	LR + LASSO	0.768	LR + LASSO	0.763	LR + mRMR100	0.753
RF + RF50	0.750	LR + mRMR50	0.764	LR + mRMR50	0.762	RF + RF100	0.752
RF + ReliefF100	0.745	SVM + LASSO	0.749	SVM + LASSO	0.751	LR + AE100	0.752
RF + ReliefF100	0.744	LR + AE100	0.744	SVM + mRMR100	0.748	RF + LASSO	0.750

Table 4. Feature selection + Logistic Regression cross validation results for data normalized with the Centered Log-Ratio transformation

Dataset	AE50	AE100	VAE	ReliefF50	ReliefF100	MIFS50	MIFS100	mRMR50	mRMR100	LASSO	RF50	RF100
CDI	0.8869	0.8878	0.8617	0.9123	0.9107	0.9049	0.9124	0.8968	0.9022	**0.9152**	0.8979	0.8965
CIR	0.6542	0.6602	0.5233	0.7479	0.7410	0.6942	0.7318	**0.8371**	0.8245	0.8103	0.7429	0.7468
CRC1	0.6817	0.6675	0.6584	0.6830	0.6849	0.6144	0.6189	**0.7112**	0.7022	0.6470	0.6639	0.6514
CRC2	**0.8206**	0.7862	0.7188	0.7249	0.7452	0.7403	0.7550	0.7504	0.7575	0.7606	0.7451	0.7799
CD1	0.7770	0.7885	0.7277	0.7424	0.7445	0.7857	0.7852	0.7865	**0.8069**	0.7968	0.7944	0.8028
CD2	0.8372	0.8380	**0.8569**	0.8154	0.8184	0.8371	0.8269	0.8525	0.8459	0.8215	0.8303	0.8279
HIV	0.7079	0.7404	0.6092	0.6987	0.6936	0.7194	0.7299	0.7364	0.7270	**0.8336**	0.6866	0.6872
IBD1	0.7911	0.8077	0.7148	0.8065	0.7960	0.7512	0.7619	0.8180	0.8225	**0.8653**	0.7944	0.7849
IBD2	0.6413	0.6197	0.6512	0.6647	0.6783	0.6942	0.6998	0.6961	0.6979	0.6842	**0.7240**	0.6942
MHE	0.6523	0.6884	0.6771	0.4566	0.4604	0.7110	0.7157	0.7183	**0.7651**	0.7191	0.6888	0.6973
OB	0.8589	**0.9060**	0.8671	0.7817	0.7805	0.7417	0.7967	0.8529	0.8791	0.8650	0.7718	0.8035
PAR1	0.6636	0.6459	0.6341	0.6888	0.7002	0.5981	0.6227	0.7130	**0.7293**	0.6787	0.6432	0.6522
PAR2	0.7291	**0.7529**	0.7197	0.6754	0.6861	0.6638	0.6964	0.7213	0.7219	0.7480	0.7152	0.7328
PAR3	0.7403	0.7569	0.6973	0.7081	0.7147	0.7206	0.7482	0.7724	**0.7804**	0.7596	0.7615	0.7722
ART	0.6368	0.6098	0.5590	0.6581	**0.6717**	0.5270	0.5159	0.6000	0.6115	0.6077	0.5943	0.5835

Results on some datasets like Obesity or CD1 improve when using top 100 features suggesting a more detailed analysis should be done when building a model with the mRMR algorithm.

Mutual Information (MIFS) selection achieves moderate results in feature selection, but as expected, tends to suffer from significant redundancy with MIFS100 not being significantly better than MIFS50 (see figure 8).

LASSO-based feature selection emerges as a competitive alternative to mRMR, achieving robust AUC scores (the highest at CDI, HIV and IBD1) that are very close to the best-performing method, with no significant differences found compared with mRMR.

Relief-based methods do not perform particularly well on these datasets except for some interesting exceptions. On CDI and Arthritis (ART) datasets they obtained top results, with some difference compared with other feature selection methods in the case of Arthritis (AUC = 0.672).

Feature selectors based on RF importance scores display intermediate performance in this comparative. Selecting the top 100 features (RF100) results in a slight performance improvement compared to selecting only 50 features (RF50). The statistical analysis via Finner's test highlights that RF's performance is not significantly different from that of competitive methods like mRMR50 and LASSO. Concurrently, RF does not demonstrate a statistically significant advantage over several of the lower-performing methods, including the ReliefF and Mutual Information variants.

Autoencoders results place them in a situation analogous to that of Random Forest, positioned in a middle ground in terms of performance. In particular, Finner's test did not show significant differences for AE100, neither compared with the top-performing methods nor with some of the weaker ones, such as ReliefF50 and MIFS50. When constrained to a latent space of 50 dimensions (AE50) performance was less favorable. Finally, variational Autoencoders (VAE) displayed very poor performance on most datasets.

For the sake of completeness, we also analyzed the feature selec-

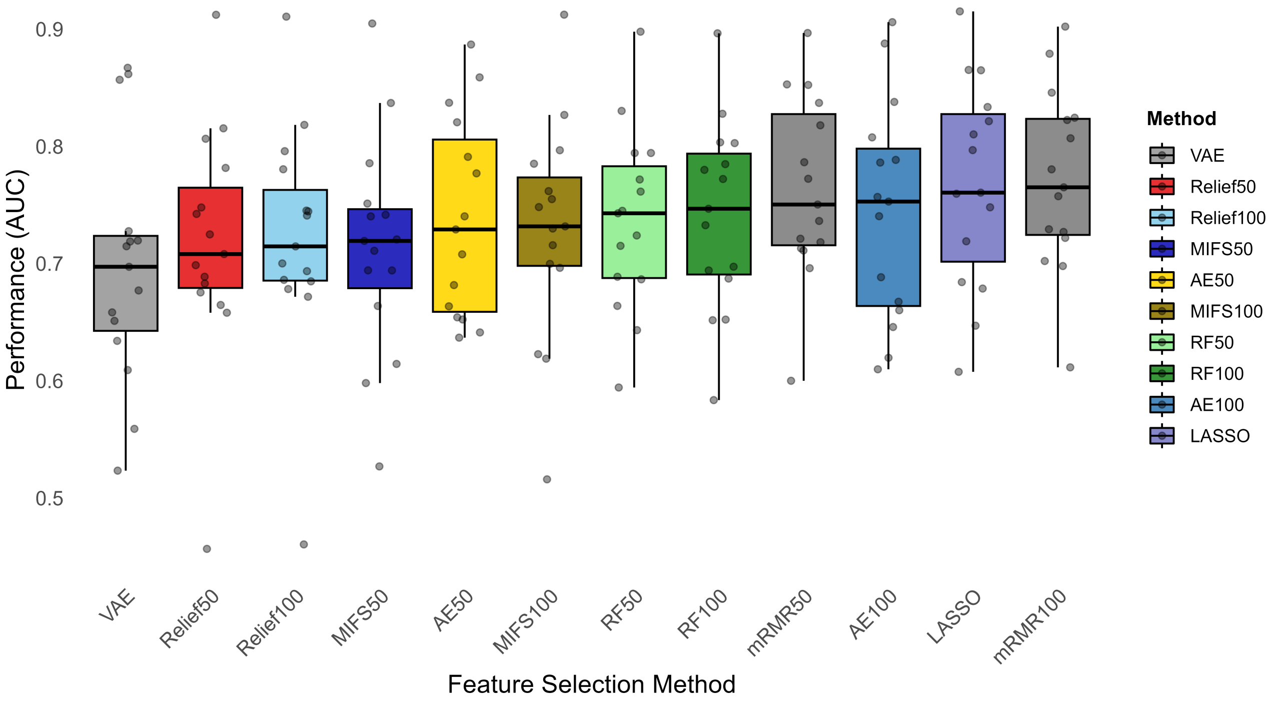

Figure 7. CLR + Feature Selection + Logistic Regression performance comparison

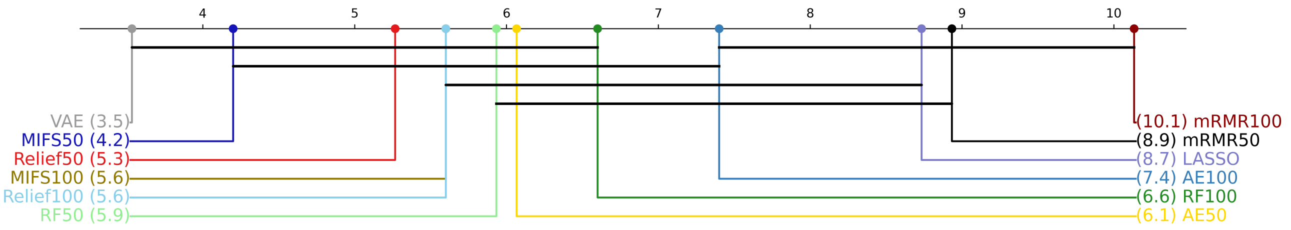

Figure 8. Finner's test results critical distance diagram for Feature selection + Logistic Regression pipelines with CLR normalization. Ranks from Friedman's test with p-value $= 8.6 \cdot 10^{-7}$

tion performance using all normalizations and classifier combinations. The top results are similar to those of CLR Logistic Regression, with mRMR100, mRMR50, and LASSO performing the best. The Friedman test confirmed significant differences between groups, and the Finner post hoc test (Supplementary Figure S3) indicated that mRMR100 was still the top-performing method. Additionally, in this global comparison, Random Forest importances and ReliefF selectors performed significantly better than autoencoders across normalizations. Finally, VAE, MIFS50 and MIFS100 exhibited even weaker performance in this comparison.

mRMR selection outperforms Mutual Information

Motivated by the observation that mRMR100 was the best-performing method in our global comparison, we conducted a focused analysis of mRMR for its potential to extract smaller and more interpretable predictive feature sets. We compared mRMR with Mutual Information (MI) for feature selection, employing an incremental approach where two new variables from the feature selection ranking were added at a time to evaluate their contributions (see figure 9).

The results on most datasets demonstrated that mRMR consistently outperforms MI in identifying relevant features, with mRMR-selected features yielding better performance. This incremental approach is aligned with our statistical analysis, which showed significant differences between mRMR and MI for both small (50 features) and larger (100 features) subsets.

Two different trends are shown at figure 9. CD2 and CIR plots illustrate that in some scenarios, less than 50 features are enough to obtain near optimal sets, while PAR2 and OB model performance keeps improving when adding more than 50 features.

To illustrate the practical output of the feature selection process, we display taxa selected by the mRMR algorithm for dataset PAR3 on Table 5. The method identified the most informative bacterial taxa after CLR transformation that are most relevant for the classification task. Notably, the selected taxa exhibit a high diversity of genera.

LASSO performance remarks

Classic LASSO feature selection has also been applied to 16S compositional data, being the CLR normalization the most straightfor-

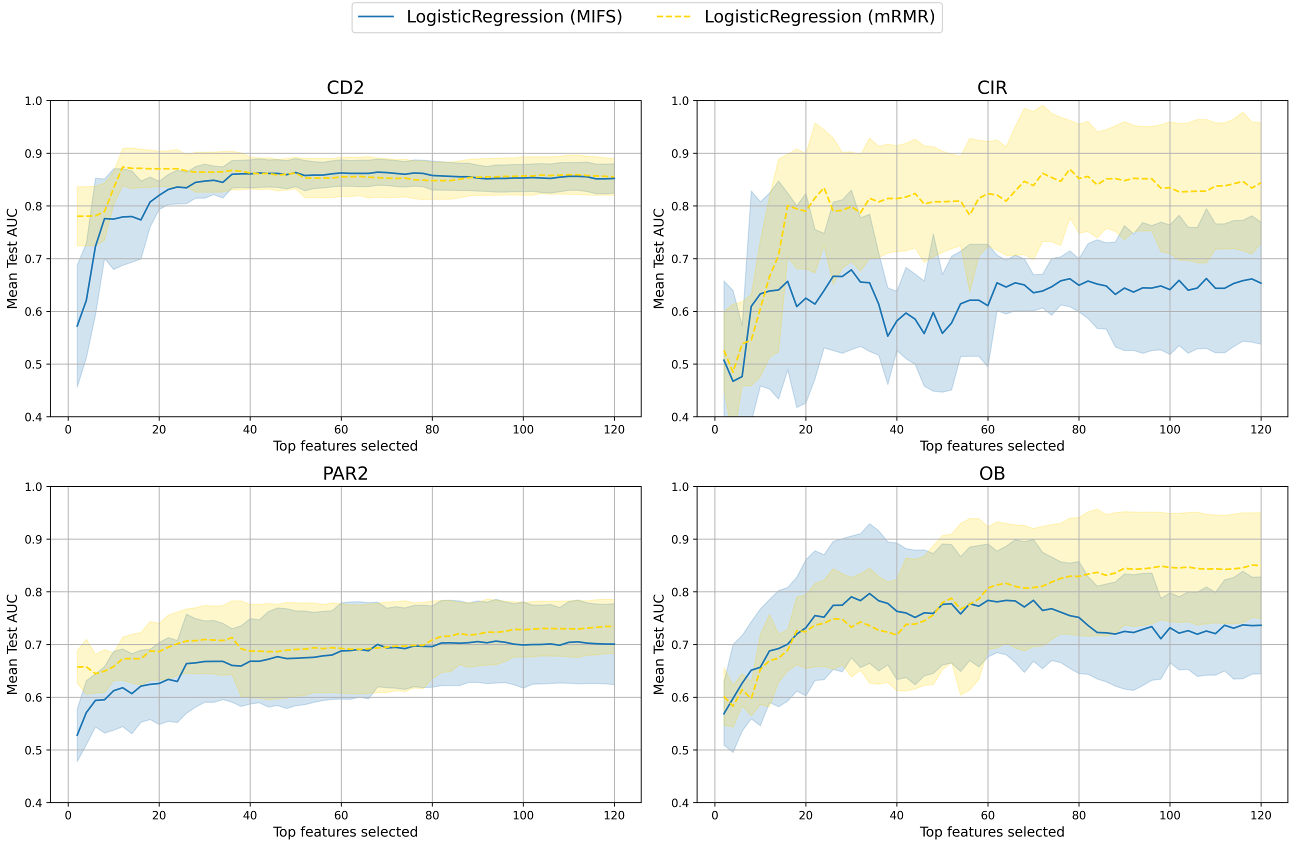

Figure 9. Cross Validation of Logistic Regression models with incremental number of features provided by Mutual Information and mRMR. Datasets CD2 and CIR provide examples of optimal feature sets with less than 50 features. PAR2 and OB sets keep improving beyond 50 and 100 features.

Table 5. Top 12 taxa ranking provided by mRMR feature selection after CLR for PAR3 dataset.

Phylum	Class	Order	Family	Genus
Firmicutes	Clostridia	Clostridiales	Lachnospiraceae	Roseburia
Firmicutes	Clostridia	Clostridiales	Family_XI	Ezakiella
Firmicutes	Bacilli	Bacillales	Not assigned	Not assigned
Firmicutes	Clostridia	Clostridiales	Ruminococcaceae	Butyricicoccus
Firmicutes	Clostridia	Clostridiales	Lachnospiraceae	Lachnoclostridium
Firmicutes	Bacilli	Lactobacillales	Streptococcaceae	Streptococcus
Proteobacteria	Gammaproteobacteria	Enterobacteriales	Enterobacteriaceae	Escherichia/Shigella
Proteobacteria	Gammaproteobacteria	Betaproteobacteriales	Burkholderiaceae	Delftia
Firmicutes	Clostridia	Clostridiales	Ruminococcaceae	Ruminococcaceae_UCG-013
Firmicutes	Bacilli	Lactobacillales	Streptococcaceae	Streptococcus
Actinobacteria	Actinobacteria	Bifidobacteriales	Bifidobacteriaceae	Bifidobacterium
Firmicutes	Clostridia	Clostridiales	Lachnospiraceae	Lachnospiraceae_UCG-004

ward preprocessing approach [32]. As we showed before, relative abundances did not fit well this method. Our results show that this method achieves comparable performance across datasets against more complex feature selection algorithms. No statistical differences were found with well performing methods like mRMR (see posthoc test results in Figure 8).

However, LASSO tends to select a larger number of features compared to methods like mRMR, particularly in larger datasets. As shown in Table 6 , the number of selected features by LASSO is higher in datasets with more samples. This raised the need to verify whether using only the top-50 or top-100 features selected by LASSO would be sufficient to approach the best performance.

The implementation we used takes 10^{-5} as threshold to consider the absolute importance of a feature to be shrunk to zero and not selected. To assess the influence of this parameter, we repeated the analysis taking only the 50 and 100 most important variables, instead of all the variables not shrunk to zero. Results at Supplementary Table T7 show that differences between the threshold approach and the Top-100 approach are minimal. Even selecting the top 50 features is enough to achieve similar results for most datasets considered.

We also measured computing time performance for feature selection algorithms (see Supplementary figure S4). Random Forest importances and LASSO stand out as the quickest, efficiently handling large datasets. Mutual Information and Variational Autoencoders (VAE) also performed swiftly. In contrast, algorithms like Minimum Redundancy Maximum Relevance (mRMR) and Autoencoders fall into an intermediate speed category; they were slower than LASSO but still manageable for moderate datasets. On the other hand, ReliefF was noticeably slower, specially with larger sample sizes.

Table 6. Number of features selected by LASSO (mean of the cross validation), sorted by number of features of the dataset.

Dataset	Selected Features	Features	Samples
CRC2	60.32	837	102(46, 56)
IBD2	71.12	1496	114(68, 46)
IBD1	56.28	2742	91(67, 24)
CD1	70.32	3547	140(78, 62)
CD2	75.60	3547	160(68, 92)
CDI	115.88	3456	336(93, 243)
CIR	47.88	3104	77(51, 26)
MHE	51.00	3104	77(26, 51)
OB	126.44	6386	281(220, 61)
CRC1	217.68	6920	490(229, 261)
PAR2	188.72	6844	333(201, 132)
PAR1	100.52	10232	148(74, 74)
ART	75.32	10733	114(86, 28)
PAR3	295.96	12198	507(323, 184)
HIV	143.84	14425	350(293, 57)
Mean	**113.13**		

Parkinson Cross-cohort Generalization

In order to assess the proposed pipelines on microbiome datasets, and further see its operation in identifying microbial signatures on even external datasets, we used datasets PAR2 and PAR3, which targeted the same hypervariable region. However, their dimensionality differed, with 6844 features at PAR2 and 12198 at PAR3. To address this difference, we trained models using the 2360 features shared between them. While both datasets were collected following uniform protocols, the analysis performed at [29] revealed batch effects between datasets. Thus, to avoid confounding we did not mix datasets while training, instead we trained the pipelines in one dataset and tested their generalization score in the other in a cross-cohort scheme.

We independently trained Logistic Regression models with feature selection using mRMR and LASSO on the subset of 2360 intersectional features. We performed an incremental analysis, evaluating classification performance by sequentially adding two features at a time (see figure 10). Comparing the performance of features selected by mRMR and LASSO revealed that mRMR-selected features exhibited better generalization in this case study.

Pipelines trained with dataset PAR3 and tested at PAR2 achieved 0.722 AUC with only 10 microbial markers, reaching 0.743 AUC with 100 for the mRMR+LR pipeline. However, LASSO+LR only reached 0.659 AUC with the top 100 selected features. Conversely, pipelines trained with dataset PAR2 and tested at PAR3 achieved similar performance for mRMR and LASSO with the top 50 features. Again, when considering 100 features, mRMR+LR pipeline obtained an AUC of 0.715 and LASSO+LR only obtained an AUC of 0.675.

Discussion

Normalization methods play a crucial role in microbiome data analysis, influencing the performance of classification tasks. A recent study showed that CLR normalization consistently outperforms RA for Logistic Regression models when classifying microbiome data [33]. Our findings corroborate this result for Logistic Regression, with CLR normalization yielding superior performance over RA across datasets. Similarly, but with slightly worse performance, SVM exhibited the same behavior across normalization methods. However, we proved this trend does not generalize across all classifiers. The global comparison regardless of the classifier chosen shows that while specific classifier performance varies with normalization, the overall impact across models selected is minimal.

The focused evaluation per normalization revealed that Random Forest performs exceptionally well with RA while performing poorly with CLR and logRA normalizations. This result underscores the importance of tailoring normalization methods to specific algorithms, as different models exhibit varying sensitivities to the transformed data distribution. Random Forest good performance using relative abundance data is aligned with the comparative conducted at [34], where CLR transformation was not applied. A plausible explanation

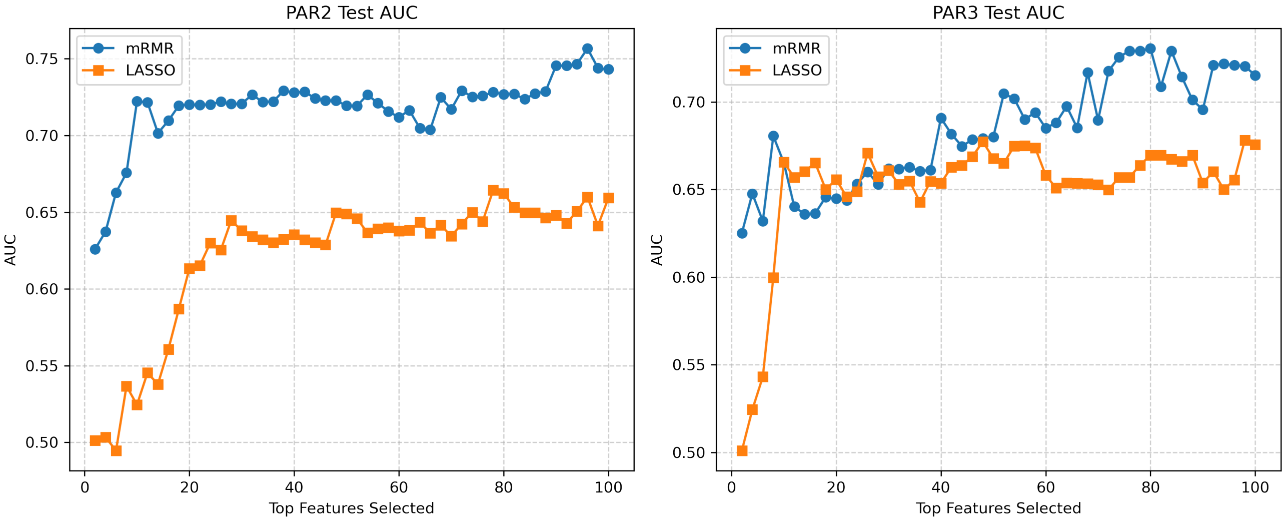

Figure 10. Cross-cohort performance comparison for Parkinson datasets (PAR2 and PAR3). Logistic Regression models were trained in one dataset and tested in the other, this plot shows the test AUCs considering top features incrementally.

is that decision trees, the core components of Random Forest and Boosting, operate by splitting data with decision thresholds, making them less affected by the compositional and interdependent nature of the data.

Moreover, the comparison between best classifiers for each normalization technique did not reveal a big impact on performance. This suggests that while method-specific normalization optimizations can enhance individual classifier outcomes, the overall performance remains stable across different approaches for baseline disease classification.

Boosting and K-Nearest Neighbors displayed stable performance across normalization methods while they do not achieve top results in any of them. KNN generally underperformed relative to other classifiers. This result is likely attributable to the sparsity inherent in microbiome data, which poses challenges for distance-based algorithms like KNN. This underperformance is not addressed properly by any normalization (best performance was obtained with PA), as the root issue seems to lie in the high sparsity of the dataset rather than the compositional nature of the data.

Additionally, the stability of PA across classifiers is remarkable. Our results are aligned with [35] and [36] studies, where Presence-Absence normalization yielded performance comparable to that of abundance-based transformations.

On the other hand, rarefaction, even in its quantile-based variant, generally leads to decreased classification performance, likely due to information loss from discarding potentially relevant counts. This could be particularly concerning in datasets from domains like the reproductive microbiome, where highly dominant species [37] coexist with low-abundance taxa that may be lost during rarefaction. Nevertheless, the improved performance of quantile-based rarefaction compared to the standard rarefaction approach may suggest a mild outlier removal effect, excluding poorly sequenced samples that differ from the overall trend of the dataset.

While these findings highlight the critical interplay between normalization strategies and machine learning algorithms, they also paved the way for further analysis. CLR transformation was found to enable a better-performing feature selection. This enhancement in performance can be attributed to the way CLR transformation addresses the compositional issue. Data is transformed from the simplex into a hyperplane in the euclidean space, which makes unconstrained statistical methods applicable [38], thus likely making data more suitable to feature selection methods that implicitly use these techniques.

When comparing feature selection methods, results shown at table 4 and critical distance diagram (figure 8) state clearly that mRMR and LASSO obtained top performance on most datasets. Notably, mRMR50 ranked slightly higher than LASSO while using fewer features on average.

mRMR selection obtains compact predictive sets

The superior performance of mRMR can be attributed to the balance of its design that generates more compact sets of informative features, which in some scenarios can be smaller, as evidenced by the incremental analysis we conducted (figure 9). These findings highlight mRMR's value as a robust and interpretable feature selection method for key attributes discovery in complex microbiome datasets.

A notable example of mRMR's effectiveness is demonstrated in the dataset PAR3, where the method identified a highly informative subset of less than 50 genera. These features exhibited strong associations with the target condition, achieving comparable performance to larger feature sets selected by other methods. Furthermore, these findings are consistent with results reported in the original study by [29], which highlighted the significance of reduced abundance of the genera Roseburia and Butyricicoccus, as well as the family Lachnospiraceae, in Parkinson's disease cases, alongside an observed overabundance of Bifidobacterium. All of these bacteria were found in the top 12 ranking provided by mRMR (table 5).

This result remarks the power of mRMR in focusing on the most relevant taxa, while minimizing redundancy coming from the hierarchical relationships between species of bacteria. Thus, resulting in compact biologically meaningful sets of features. The interpretability of this subsets is particularly valuable for translational applications, as it provides clear targets for further biological or clinical validation.

Autoencoders struggle to trade off performance and feature reduction

During experimentation, we tested various autoencoder architectures to determine the optimal configuration for microbiome feature selection. AE100 performed reasonably well, ranking highly on some datasets. However, the modest results obtained by AE50 indicated that larger latent spaces are required to achieve superior classification performance. However, this improvement would came at the cost of reduced interpretability and the necessity of handling a high-dimensional latent representation.

Additionally, we experimented with a Variational Autoencoder

Table 7. AUC performance comparison of different pipelines on microbiome datasets. For each dataset, the highest AUC is displayed in bold. The two best baseline combinations are listed first, followed by the top three complete pipelines combining Feature Selection and Logistic Regression. Reduction (%) indicates the percentage of features discarded by the feature selection method, relative to the total number of input features.

Dataset	Input Features	Baseline		CLR + LASSO + LR		CLR + mRMR100 + LR		CLR + mRMR50 + LR	
		RA + RF	CLR + LR	AUC	*Reduction* (%)	AUC	*Reduction* (%)	AUC	*Reduction* (%)
CDI	3456	0.8973	**0.9171**	0.9152	*96.65*	0.9022	*97.11*	0.8968	*98.55*
CIR	3104	**0.8468**	0.8147	0.8103	*98.46*	0.8245	*96.78*	0.8371	*98.39*
CRC1	6920	0.6882	0.6651	0.6470	*96.85*	0.7022	*98.55*	**0.7112**	*99.28*
CRC2	837	**0.8247**	0.7960	0.7606	*92.79*	0.7575	*88.05*	0.7504	*94.03*
CD1	3547	0.8073	**0.8253**	0.7968	*98.02*	0.8069	*97.18*	0.7865	*98.59*
CD2	3547	0.8297	0.8229	0.8215	*97.87*	0.8459	*97.18*	**0.8525**	*98.59*
HIV	14425	0.7165	0.8257	**0.8336**	*99.00*	0.7270	*99.31*	0.7364	*99.65*
IBD1	2742	0.7818	0.8314	**0.8652**	*97.95*	0.8225	*96.35*	0.8180	*98.18*
IBD2	1496	0.6893	0.6683	0.6842	*95.25*	**0.6979**	*93.32*	0.6961	*96.66*
MHE	3104	**0.8116**	0.7835	0.7191	*98.36*	0.7651	*96.78*	0.7183	*98.39*
OB	6386	0.8686	**0.9123**	0.8650	*98.02*	0.8791	*98.43*	0.8529	*99.22*
PAR1	10232	0.6898	0.6806	0.6787	*99.02*	**0.7293**	*99.02*	0.7130	*99.51*
PAR2	6844	0.7476	**0.7629**	0.7480	*97.24*	0.7219	*98.54*	0.7213	*99.27*
PAR3	12198	0.7770	**0.7844**	0.7596	*97.57*	0.7804	*99.18*	0.7724	*99.59*
ART	10733	0.6048	**0.6439**	0.6077	*99.30*	0.6115	*99.07*	0.6000	*99.53*

architecture, which is designed to encode input data into a probabilistic latent space. Despite its theoretical advantages, such as generating smoother latent spaces and capturing underlying data distributions, the VAE did not perform well on our datasets. It failed to effectively model the complexity of the microbiome data, resulting in worse classification outcomes compared to traditional autoencoders.

A significant limitation of autoencoders, particularly in the context of microbiome research and medical applications, is their inherent lack of interpretability. The latent space representations they generate are often considered "black boxes," making it difficult to directly link features to biological phenomena or actionable insights. While some methods for interpreting autoencoder outputs exist, they remain limited in scope and application [39, 40].

This lack of transparency poses a challenge, especially in healthcare-related fields where decision-making algorithms are increasingly scrutinized for explainability [41].

Taking into account these considerations, autoencoders may not be the most suitable alternative for identifying biologically meaningful signature sets. Simpler and more transparent methods, such as mRMR and LASSO, may be better suited for this specific task.

LASSO remains as a powerful option

Lasso feature selection has shown to perform well at microbiome-based disease classification paired with Logistic Regression and Random Forest for inflammatory bowel disease [42, 43]. Our results are aligned with these studies, showing that this method achieves comparable performance across datasets against more complex feature selection algorithms.

Overall, the mRMR + Logistic Regression and Lasso + Logistic Regression pipelines (with CLR) proved to be powerful strategies for improving the robustness and quality of the models. Table 7 shows that these feature selection pipelines achieve at least similar performance, if not better, than the best baseline classification models. Moreover, feature selection offers a greater advantage, a massive reduction of the dimensionality. As we can see, a reduction of around 99 % of the features can be achieved and the selected ones are able to display similar predictive performance as using all of them. This reduction helps models to reduce overfitting and avoid learning noise or spurious patterns coming from features that are irrelevant to the biological signal. Additionally, a reduced set of features makes it easier to interpret and understand the underlying factors influencing the model's predictions.

Generalizing Across Cohorts

One of the key challenges in microbiome data analysis is to obtain feature sets that capture generalizable patterns, ensuring that the selected features are robust and applicable across different datasets rather than being influenced by specific dataset characteristics. For this reason, we conducted the cross-cohort study using our pipelines with the Parkinson data. It is important to note that this analysis was performed as a proof of concept. While the observed results are promising, the ability to consistently achieve such levels of generalization remains as an open question. To the best of our knowledge, although cross-cohort generalization is a desirable goal, it is rarely reported in 16S microbiome studies.

Our results show that the features obtained in this case study can reach a good level of generalization. The improved generalization of mRMR-selected features suggests that its greedy procedure to remove redundancy also can help to mitigate dataset-specific biases, allowing generalizable discoveries.

Another key observation is that models trained on PAR3 and tested on PAR2 achieved better generalization than the reverse. This is a reasonable outcome given the larger sample size in PAR3, which provides a broader representation of microbial variability at the training step. Larger datasets tend to capture more robust patterns, making them able to train models with lower generalization error.

Potential implications

The findings of this study offer new lines of research that could expand the scope and applicability of microbiome disease classification pipelines. The following potential implications highlight broader research opportunities:

- **Hybrid Approaches:** The different feature selection methods explored could be complemented by hybrid strategies, such as employing efficient greedy methods for initial filtering, followed by computationally-intensive techniques like wrappers or evolutionary algorithms to refine the microbial signatures. Such combinations may obtain very reduced yet informative feature subsets.
- **Evaluation Across Harmonized Datasets:** The harmonization of datasets provides an opportunity for comprehensive meta-analyses. Evaluating feature selection methods across integrated datasets can help generalize findings, ensuring they are robust to variations in data collection, preprocessing, and experimental conditions. Validation of results across external studies

would further establish the reliability of identified biomarkers.

- **Advancing Explainability for Clinical Integration:** Developing explainability interfaces and metrics tailored to microbiome data can help to close the gap between bioinformatic discoveries and practical clinical applications. By enabling to understand the reasons behind feature selection and classification decisions, these tools would promote confidence in utilizing biomarkers for aiding diagnostics.
- **Towards Standardization and Collaboration:** As microbiome research continues to expand, standardization of feature selection practices, facilitated by reproducible workflows, will be essential. Collaborative platforms that integrate harmonized data, past results and interpretability frameworks could accelerate discoveries in microbiome research.

Conclusion

Taken together, our study provides some recommendations on how to improve biomarker discovery through 16S microbiome disease classification without sacrificing performance. Random Forest showed strong behavior for raw relative abundances and turned out to be good baselines for pure performance. Additionally, Presence-Absence normalization yielded equivalent performance to that of abundance-based transformations. On the other hand, CLR normalization combined with Logistic Regression helps to cope with compositionality at baseline classification and improves feature selection. Among feature selection methods, mRMR100 consistently enhanced predictive power while maintaining interpretability and compactness. While mRMR50 also ranked better, LASSO must also be considered because of its similar performance and quicker computation.

Future studies are advised to match normalization and feature selection approaches to their chosen classifiers. They should also establish a trade-off between feature set sizes and predictive accuracy, in order to maximize the robustness and relevance of the identified microbial features.

Methods

16S rRNA data

The most usual sequencing technique to analyze human microbiome is 16S ribosomal RNA amplicon analysis [44]. The 16S gene is used for taxonomic and phylogenetic studies as it is universally present between different species of bacteria and archaea. It is approximately 1600 base pair long and contains nine hypervariable regions (V1-V9) with different conservation degree that can provide signatures of different bacterial species [45]. The conserved regions flanking the hypervariable region of interest can be used for PCR amplification of targets and posterior sequencing via the Illumina framework [46] or previously via the discontinued 454 pyrosequencing [47].

The resulting sequences are then clustered into operational taxonomic units (OTUs) [48] or resolved into amplicon sequence variants [49]. Finally, they are compared against a reference database in order to identify their likely taxonomy [50]. At this point raw data has transformed into an abundance table with the number of reads of each sequence for each sample, suited for data analysis and machine learning.

Preprocessing: Filtering and Normalization

As data selected was already assembled into OTU/ASV count tables, only simple filtering was needed. Datasets with acceptable number of features were not filtered to avoid removing low-count but possibly powerful predictive OTUs. In the other hand, extremely sparse datasets, with more than 15000 features were filtered (Supplementary Table T1 provides original feature dimensionality and details for the datasets), i.e., as in [34] we removed taxa with less than 10 reads across the dataset, and features that were not present in more than the 1 % of the dataset. Final dimensions of datasets are presented at table 1. Relative abundances of taxa were then computed, this step is commonly referred to as a closure operation. These proportions naturally reside in the simplex, a constrained D-dimensional space defined as:

$$\mathbf{x}_{\text{ra}} = \left[\frac{x_1}{\sum_{i=1}^{D} x_i}, \frac{x_2}{\sum_{i=1}^{D} x_i}, \ldots, \frac{x_D}{\sum_{i=1}^{D} x_i} \right] \in \mathcal{S}^{D-1}$$

$$\mathcal{S}^{D-1} = \left\{ \mathbf{x} \in \mathbb{R}^D \mid x_i \geq 0, \sum_{i=1}^{D} x_i = 1 \right\}$$

Centered log-ratio normalization was applied to address the compositional nature of microbiome data and compared its performance with RA and other transformations, including log-transformed relative abundances (logRA), and Presence-Absence (PA) encoding.

Aitchison's CLR normalization [51] transforms compositional data from the simplex into the Euclidean space by taking the logarithm of each feature's relative abundance divided by the geometric mean $G(x)$ of all features within a sample.

$$\mathbf{x}_{clr} = \left[\log\left(\frac{x_{\text{ra}1}}{G(\mathbf{x}_{\text{ra}})}\right), \log\left(\frac{x_{\text{ra}2}}{G(\mathbf{x}_{\text{ra}})}\right), \ldots, \log\left(\frac{x_{\text{ra}D}}{G(\mathbf{x}_{\text{ra}})}\right) \right]$$

However, due to the presence of zero counts in microbiome datasets (common when taxa are not detected in a given sample) it is necessary to introduce a pseudocount to avoid undefined logarithmic calculations. The choice of pseudocount remains a critical yet unresolved issue. Different pseudocount values can impact the results, as they affect the ratios between features and distribution of the transformed data. While commonly used values such as 0.5, 1, or small constants close to zero are practical solutions, there is no consensus on an optimal choice. We applied a pseudocount $p = 0.5$ to all datasets as done in the limma-voom approach [52], to allow for the CLR and logRA normalizations.

Finally, the Presence-Absence (PA) transformation is a binary representation where each feature is set to 1 if its relative abundance is greater than zero, and 0 otherwise.

$$x_{\text{pa}_i} = \begin{cases} 1 & \text{if } x_{\text{ra}_i} > 0 \\ 0 & \text{otherwise} \end{cases}$$

Rarefaction is another commonly used preprocessing step in microbiome studies, performed directly on count data, before the previously defined normalizations. It involves randomly subsampling each sample reads (without replacement) to the same sequencing depth to mitigate biases introduced by varying library sizes [53]. While it can help control for differences in sampling depth, rarefaction discards data and introduces stochasticity, which can reduce statistical power.

Feature Selection Techniques Overview

Feature selection is a critical step in high-dimensional data analysis, as it reduces the number of variables while retaining the most informative ones. This enhances interpretability and may improve model performance. By selecting a small number of features, such

Table 8. Summary of feature selection methods used in the study.

Method	Description
Mutual Information	Measures the dependence between each feature and the target variable to select the most informative ones.
mRMR	Selects features greedily balancing high relevance to the target and low redundancy among themselves.
LASSO	Features selected by a L1 regularized regression model that enforces sparsity, shrinking some coefficients to zero.
ReliefF	Assigns feature weights based on how well they differentiate between neighboring instances of different classes.
Random Forest Importances	Ranks features based on their contribution to reducing impurity in decision trees within the ensemble.
Shallow Autoencoder	Learns a compressed representation of input data through unsupervised encoding and decoding.
Variational Autoencoder	Probabilistic autoencoder that learns a compressed latent space representation by modeling input data as a distribution.

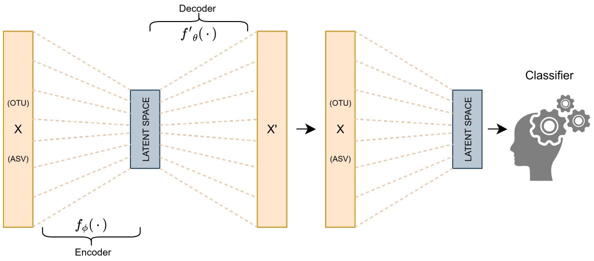

Figure 11. Shallow autoencoder feature reductor diagram. Latent space is calculated via optimization of the reconstruction loss $L(x, x') = ||x - x'||^2 = ||x - f'_\theta(f_\phi(x))||^2$.

as microbial genera or species, their biological relationships with the target diseases can be more easily identified. Moreover, removing redundant and irrelevant features can lead to an improvement of the generalization ability of the models.

In this study, we primarily focused on the filter approach, which evaluates features independently of any classification algorithm. This provides a model-agnostic ranking that can be applied across different classifiers while minimizing the risk of overfitting [54]. Additionally, we employed autoencoders to explore dimensionality reduction techniques. While not strictly a feature selection method, autoencoders identify compressed representations of the data without the use of class information. This deep learning approach is emerging as a growing-interest technique in the microbiome domain [55]. As a reference, we also included LASSO, a widely used technique in the few studies addressing this problem, to benchmark the performance of our approaches.

Mutual Information and mRMR

Mutual Information (MI) is a fundamental concept in information theory that measures the mutual dependency between two variables [56]. It estimates how much knowledge about one variable is obtained by observing the other. One of the main advantages of MI lies in its ability to capture nonlinear relationships, unlike simpler correlation metrics that only account for linear ones. This flexibility is particularly useful in biological data, where complex interactions are common. However, MI-based feature selection provides a ranking of features that has not accounted for redundancy, leading to the selection of features that may be highly correlated and thus less informative collectively. We applied the implementation available at scikit-learn [31].

The Minimum Redundancy Maximum Relevancy (mRMR) algorithm was initially designed for gene selection in transcriptomics. It aims to strike a balance between selecting features that are maximally relevant to the target variable and minimally redundant with each other [57]. Unlike simpler Mutual Information-based methods [58], mRMR imposes additional constraints to avoid selecting correlated features, which is particularly useful in high-dimensional datasets. In particular, we considered two subset sizes to retain for comparisons (50 and 100), in this way we can also evaluate the redundancy present in the data. We applied the open source implementation available at [59].

This algorithm has demonstrated its effectiveness in identifying small, biologically meaningful signatures in transcriptomics, especially in cancer studies. Applications of mRMR to 16S rRNA microbiome data have yielded mixed results, often selecting relatively large feature sets [12].

ReliefF

ReliefF is a feature selection algorithm that extends the original Relief method improving its capabilities to handle unbalanced and noisy datasets [60]. This algorithm evaluates features by estimating their ability to distinguish between instances that are near each other (nearest hits and misses), leveraging a nearest-neighbor approach to assess the relevance of features. Unlike most filter methods, ReliefF considers interactions among features, making it particularly suitable for high-dimensional datasets where complex relationships may exist. We applied the ReBATE open source implementation [61].

Autoencoders

Autoencoders are a specialized class of artificial neural networks that can learn efficient compressed data representations without the information from the labels. Its architecture consists of two main parts, an encoder layer that aims to transform the data into a reduced representation, the latent space. From the output of this layer, a decoder tries to rebuild the original input. During training, the loss function measures the difference between input and output layers, by minimizing this reconstruction loss, the latent space captures the most important features of the input data. While autoencoders are not a traditional Feature Selection method, they have been pointed out as an interesting hypothesis for task-adapted dimensionality reduction that can cope with sparse matrices and low number of samples. [55, 62] . For our study we used DeepMicro [63] , a deep learning framework designed to allow an effective representation of microbiome profiles, to learn the latent space representation as a previous step for the classification task. In particular, we used the shallow autoencoders that have only one hidden layer, as they showed the most promising results in the DeepMicro case studies and also because of the small sample sizes of some of the considered datasets. We chose 2 different latent space sizes (50 and 100), allowing to compare with the other algorithms, and a variational autoencoder (50). DeepMicro also offers the later classification step, but to adapt autoencoders to our validation strategy we preferred to use only the latent representation learning step.

LASSO

Least Absolute Shrinkage and Selection Operator (LASSO) is a regression-based method widely used for feature selection. By effectively shrinking the coefficients of less relevant features to zero it stands as a common FS method for high-dimensional datasets. In microbiome studies, LASSO is frequently employed either as a standalone feature selection method or as part of a pipeline paired with Logistic Regression [32, 64]. In our case, the Logistic Regression classifier uses L2-regularization, therefore when combined in a pipeline, both techniques will be hybridized.

Random Forest Feature Importance
Random Forest is an ensemble learning method that can also be used for feature selection by evaluating the importance of each feature in the model's construction [65]. The core idea is to build a large number of decision trees, with each tree trained on a random subset of the data. The importance of a feature is calculated by measuring how much it contributes to reducing impurity across all the trees. When a particular feature is repeatedly selected at the top of the trees for splitting the nodes, it receives a higher importance score. This provides a feature relevance metric that accounts for interactions between features by construction.

Classification Models, Tuning and Validation

Our study leveraged both ensemble and traditional classification algorithms to evaluate the impact of feature selection and normalization techniques. Ensemble methods have been demonstrated as very powerful classifiers, among which Random Forest (RF) and eXtreme Gradient Boosting decision trees (XGBoost) are the two most popular ones. In addition to them, we employed traditional classifiers, including Support Vector Machines (SVM), Logistic Regression (LR) and k-Nearest Neighbors (KNN).

In order to get a robust measure of generalization performance, each model was validated through 5 times repeated nested 5 fold cross validation (see figure 12). To ensure fair comparisons, all classifiers underwent hyperparameter optimization using grid search in the inner validation loop. This computationally intensive process was selected to ensure robust performance estimates and minimize possible bias and potential overfitting from the model parameters selection [66].

The Area Under the Receiver Operating Characteristic Curve (AUC) was chosen as the objective metric, as it is the most commonly used performance measure in microbiome classification studies. AUC offers a general evaluation of model performance across varying decision thresholds, being less sensitive to imbalance in datasets, where accuracy may fail to capture the true predictive power of a classifier.

Logistic Regression
Logistic Regression is a linear model that is commonly used for binary classification tasks. It estimates the probability of an instance belonging to a particular class by fitting the input features to a logistic function. In our analysis, we incorporated L2 regularization (Ridge) to improve model performance and prevent overfitting. This technique adds a penalty term proportional to the square of the coefficient values to the loss function [67].

Support Vector Machine
Support Vector Machines classify data by finding the optimal hyperplane that maximally separates classes in a high-dimensional feature space. SVM can handle non-linear boundaries using kernel functions (radial basis or polynomial) and is effective in high-dimensional settings [68]. In our case, we selected a radial-basis kernel, as the results of [69] stated its universality, meaning it can approximate any continuous function arbitrarily well given sufficient data. This property implies that the RBF kernel can generalize other kernels under appropriate conditions.

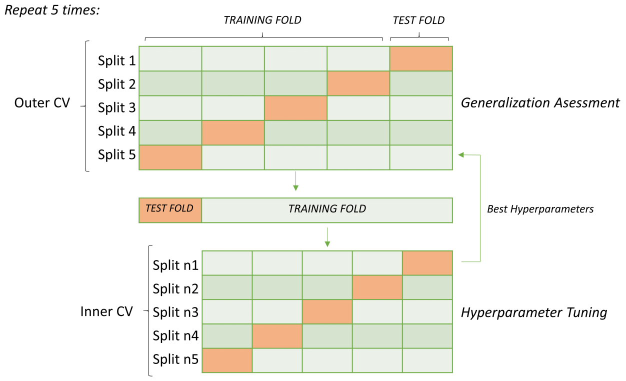

Figure 12. Repeated Nested Cross Validation procedure.

Random Forest
Random Forest is an ensemble learning method that constructs multiple decision trees on different sets of features and samples (bagging) during training and outputs the mode of their predictions. By aggregating predictions from numerous trees and data samples RF reduces overfitting and improves robustness. [65]

Boosting
Boosting is another ensemble learning approach that builds a strong classifier by sequentially combining a set of weak models. Trees are trained iteratively, with each new model focusing on correcting the instances that were misclassified by the previous models. For our analysis, we employed XGBoost (Extreme Gradient Boosting), a powerful and widely adopted implementation of gradient boosting known for its high performance and scalability. [70]

K-Nearest Neighbhor
KNN is a non-parametric, lazy-learning method that classifies samples by majority vote provided by the classes of their k closest neighbors in the feature space. It requires no explicit training but scales poorly with large datasets due to its reliance on distance computations (e.g., Euclidean or Manhattan). Performance depends strongly on the choice of k, which in conjunction with the distance metric need to be tuned properly [71].

Statistical Analysis

Comparing results of multiple algorithms across multiple datasets requires the use of statistical tests to avoid reaching conclusions due to random chance [72].

To analyze pairwise differences between pairs of classifiers or normalization techniques, we employed the Wilcoxon signed-rank test. This paired non-parametric test was chosen due to its suitability for scenarios where the assumptions of normality of parametric tests may not hold.

We also employed the Friedman test followed by the Finner posthoc procedure to analyze the differences between multiple classifiers and feature selection methods. This approach was chosen by evidences on superior power compared to other procedures [73], which means it has a lower probability of making a type II error. The Friedman test allowed us to determine the presence of significant differences in classifier performance for each normalization, while the Finner posthoc procedure enabled us to identify which methods differed from each other in a controlled manner.

Availability of source code and requirements

- Project name: 16SMicrobiomeMLFS
- Project home page: `https://github.com/nach00gar/16SMicrobiomeMLFS`
- Operating system(s): Platform independent
- Programming language: Python, R
- Other requirements: sklearn 1.5.2, composition_stats 2.0.0, xgboost 2.1.2, keras 2.2.4, mrmr-selection 0.2.8, skrebate 0.62, DeepMicro [63], stac (Statistical Tests for Algorithms Comparison [74])
- License: GNU GPL
- SciCrunch RRID: SCR_027170

- Bio.tools ID: 16smicrobiomemlfs
- WorkflowHub ID: 1807 [75]

Data availability

Supporting data used for the analyses can be accessed by our code repository. Data was downloaded from MicrobiomeHD [15], ML-Repo [16] and [17] article repository.

Declarations

List of abbreviations

AE: Autoencoder; AUC: Area Under the Receiver Operating Characteristic Curve; ASV: Amplicon Sequence Variant; CLR: Centered Log-Ratio; FS: Feature Selection; IR: Imbalance Ratio; KNN: k-Nearest Neighbors; LASSO: Least Absolute Shrinkage and Selection Operator; LR: Logistic Regression; MIFS: Mutual Information Feature Selection; ML: Machine Learning; mRMR: Minimum Redundancy Maximum Relevancy; OTU: Operational Taxonomic Unit; PA: Presence-Absence; PCR: Polymerase Chain Reaction; RA: Relative Abundances; RF: Random Forest; rRNA: Ribosomal RNA; SVM: Support Vector Machine; VAE: Variational Autoencoder.

Disclosure of use of AI-assisted tools

Large language models [76] were used to improve the flow of certain paragraphs and to find grammatical errors. The use of the LLM does not negatively affect the empirical data and conclusions. The authors have thoroughly reviewed the text and are fully responsible for its content.

Competing Interests

The authors declare that they have no competing interests.

Funding

This work is supported by Grant PID2021-128317OB-I00 (Principal Investigators: Ignacio Rojas and Luis Javier Herrera) and PCI2023-146016-2 (Principal Investigators: Francisco Manuel Ortuño and Olga Valenzuela), funded by MICIU/AEI/10.13039/501100011033 and co-funded by the European Union.

Author's Contributions

Conceptualization, I.G., L.J.H. and F.M.O.; methodology, I.G. and L.J.H.; software, I.G.; analysis and validation, I.G.; data curation, I.G. ; writing—original draft preparation, I.G. and L.J.H.; writing—review and editing, I.R., L.J.H., and F.M.O.; supervision, I.R., L.J.H., and F.M.O.; funding acquisition, I.R., L.J.H., and F.M.O. All authors read and approved the final manuscript.

References

1. Hou K, Wu ZX, Chen XY, Wang JQ, Zhang D, Xiao C, et al. Microbiota in health and diseases. Signal Transduction and Targeted Therapy 2022 Apr;7(1):1–28. https://www.nature.com/articles/s41392-022-00974-4.
2. Akbar N, Khan NA, Muhammad JS, Siddiqui R. The role of gut microbiome in cancer genesis and cancer prevention. Health Sciences Review 2022 Mar;2:100010. https://www.sciencedirect.com/science/article/pii/S277263202100009X.
3. Núñez-Sánchez MA, Melgar S, O'Donoghue K, Martínez-Sánchez MA, Fernández-Ruiz VE, Ferrer-Gómez M, et al. Crohn's Disease, Host–Microbiota Interactions, and Immunonutrition: Dietary Strategies Targeting Gut Microbiome as Novel Therapeutic Approaches. International Journal of Molecular Sciences 2022 Jul;23(15):8361. https://www.ncbi.nlm.nih.gov/pmc/articles/PMC9369148/.
4. Zhao T, Wei Y, Zhu Y, Xie Z, Hai Q, Li Z, et al. Gut microbiota and rheumatoid arthritis: From pathogenesis to novel therapeutic opportunities. Frontiers in Immunology 2022 Sep;13:1007165. https://www.ncbi.nlm.nih.gov/pmc/articles/PMC9499173/.
5. Carabotti M, Scirocco A, Maselli MA, Severi C. The gut-brain axis: interactions between enteric microbiota, central and enteric nervous systems. Annals of Gastroenterology: Quarterly Publication of the Hellenic Society of Gastroenterology 2015 Jun;28(2):203. https://pmc.ncbi.nlm.nih.gov/articles/PMC4367209/.
6. Hernández Medina R, Kutuzova S, Nielsen KN, Johansen J, Hansen LH, Nielsen M, et al. Machine learning and deep learning applications in microbiome research. ISME Communications 2022 Oct;2(1):1–7. https://www.nature.com/articles/s43705-022-00182-9.
7. Altman N, Krzywinski M. The curse(s) of dimensionality. Nature Methods 2018 Jun;15(6):399–400. https://www.nature.com/articles/s41592-018-0019-x.
8. Pan AY. Statistical analysis of microbiome data: The challenge of sparsity. Current Opinion in Endocrine and Metabolic Research 2021 Aug;19:35–40. https://www.sciencedirect.com/science/article/pii/S2451965021000600.
9. Gloor GB, Macklaim JM, Pawlowsky-Glahn V, Egozcue JJ. Microbiome Datasets Are Compositional: And This Is Not Optional. Frontiers in Microbiology 2017;8:2224.
10. Gloor GB, Wu JR, Pawlowsky-Glahn V, Egozcue JJ. It's all relative: analyzing microbiome data as compositions. Annals of Epidemiology 2016 May;26(5):322–329. https://www.sciencedirect.com/science/article/pii/S1047279716300734.
11. Pödör Z, Hekfusz M. Comparing Feature Selection Methods on Metagenomic Data using Random Forest Classifier. Transactions on Engineering and Computing Sciences 2024 Feb;12(1):175–187. https://journals.scholarpublishing.org/index.php/TMLAI/article/view/16525.
12. Wu H, Cai L, Li D, Wang X, Zhao S, Zou F, et al. Metagenomics Biomarkers Selected for Prediction of Three Different Diseases in Chinese Population. BioMed Research International 2018;2018(1):2936257. https://onlinelibrary.wiley.com/doi/abs/10.1155/2018/2936257.
13. Human Microbiome Project Consortium. Structure, function and diversity of the healthy human microbiome. Nature 2012 Jun;486(7402):207–214.
14. McDonald D, Hyde E, Debelius JW, Morton JT, Gonzalez A, Ackermann G, et al. American Gut: an Open Platform for Citizen Science Microbiome Research. mSystems 2018 May;3(3):10.1128/msystems.00031–18. https://journals.asm.org/doi/10.1128/msystems.00031-18.
15. Duvallet C, Gibbons S, Gurry T, Irizarry R, Alm E, MicrobiomeHD: the human gut microbiome in health and disease. Zenodo; 2017. https://zenodo.org/records/569601, accessed 7 October 2024.
16. Vangay P, Hillmann BM, Knights D. Microbiome Learning Repo (ML Repo): A public repository of microbiome regression and classification tasks. GigaScience 2019 Apr;8(5):giz042. https://pmc.ncbi.nlm.nih.gov/articles/PMC6493971/.
17. Wallen ZD. Comparison study of differential abundance testing methods using two large Parkinson disease gut microbiome datasets derived from 16S amplicon sequencing. BMC Bioin-

formatics 2021 May;22(1):265. https://doi.org/10.1186/s12859-021-04193-6.
18. Scher JU, Sczesnak A, Longman RS, Segata N, Ubeda C, Bielski C, et al. Expansion of intestinal Prevotella copri correlates with enhanced susceptibility to arthritis. eLife 2013 Nov;2:e01202. https://doi.org/10.7554/eLife.01202.
19. Schubert AM, Rogers MAM, Ring C, Mogle J, Petrosino JP, Young VB, et al. Microbiome Data Distinguish Patients with Clostridium difficile Infection and Non-C. difficile-Associated Diarrhea from Healthy Controls. mBio 2014 May;5(3):10.1128/mbio.01021–14. https://journals.asm.org/doi/10.1128/mbio.01021-14.
20. Baxter NT, Ruffin MT, Rogers MAM, Schloss PD. Microbiota-based model improves the sensitivity of fecal immunochemical test for detecting colonic lesions. Genome Medicine 2016 Apr;8(1):37. https://doi.org/10.1186/s13073-016-0290-3.
21. Wang T, Cai G, Qiu Y, Fei N, Zhang M, Pang X, et al. Structural segregation of gut microbiota between colorectal cancer patients and healthy volunteers. The ISME Journal 2012 Feb;6(2):320–329. https://doi.org/10.1038/ismej.2011.109.
22. Noguera-Julian M, Rocafort M, Guillén Y, Rivera J, Casadellà M, Nowak P, et al. Gut Microbiota Linked to Sexual Preference and HIV Infection. eBioMedicine 2016 Mar;5:135–146. https://www.thelancet.com/journals/ebiom/article/PIIS2352-3964(16)30028-7/fulltext.
23. Gevers D, Kugathasan S, Denson LA, Vázquez-Baeza Y, Treuren WV, Ren B, et al. The treatment-naïve microbiome in new-onset Crohn's disease. Cell host & microbe 2014 Mar;15(3):382. https://pmc.ncbi.nlm.nih.gov/articles/PMC4059512/.
24. Papa E, Docktor M, Smillie C, Weber S, Preheim SP, Gevers D, et al. Non-Invasive Mapping of the Gastrointestinal Microbiota Identifies Children with Inflammatory Bowel Disease. PLOS ONE 2012 Jun;7(6):e39242. https://journals.plos.org/plosone/article?id=10.1371/journal.pone.0039242.
25. Willing BP, Dicksved J, Halfvarson J, Andersson AF, Lucio M, Zheng Z, et al. A pyrosequencing study in twins shows that gastrointestinal microbial profiles vary with inflammatory bowel disease phenotypes. Gastroenterology 2010;139(6):1844–1854.e1.
26. Zhang Z, Zhai H, Geng J, Yu R, Ren H, Fan H, et al. Large-Scale Survey of Gut Microbiota Associated With MHE Via 16S rRNA-Based Pyrosequencing. Official journal of the American College of Gastroenterology | ACG 2013 Oct;108(10):1601. https://journals.lww.com/ajg/abstract/2013/10000/large_scale_survey_of_gut_microbiota_associated.14.aspx.
27. Turnbaugh PJ, Hamady M, Yatsunenko T, Cantarel BL, Duncan A, Ley RE, et al. A core gut microbiome in obese and lean twins. Nature 2009 Jan;457(7228):480–484. https://www.nature.com/articles/nature07540.
28. Scheperjans F, Aho V, Pereira PAB, Koskinen K, Paulin L, Pekkonen E, et al. Gut microbiota are related to Parkinson's disease and clinical phenotype. Movement Disorders 2015;30(3):350–358. https://onlinelibrary.wiley.com/doi/abs/10.1002/mds.26069.
29. Wallen ZD, Appah M, Dean MN, Sesler CL, Factor SA, Molho E, et al. Characterizing dysbiosis of gut microbiome in PD: evidence for overabundance of opportunistic pathogens. npj Parkinson's Disease 2020 Jun;6(1):1–12. https://www.nature.com/articles/s41531-020-0112-6.
30. Hill-Burns EM, Debelius JW, Morton JT, Wissemann WT, Lewis MR, Wallen ZD, et al. Parkinson's Disease and PD Medications Have Distinct Signatures of the Gut Microbiome. Movement disorders: official journal of the Movement Disorder Society 2017 May;32(5):739–749. https://www.ncbi.nlm.nih.gov/pmc/articles/PMC5469442/.
31. Pedregosa F, Varoquaux G, Gramfort A, Michel V, Thirion B, Grisel O, et al. Scikit-learn: Machine Learning in Python. Journal of Machine Learning Research 2011;12(85):2825–2830. http://jmlr.org/papers/v12/pedregosa11a.html.
32. Susin A, Wang Y, Lê Cao KA, Calle ML. Variable selection in microbiome compositional data analysis. NAR Genomics and Bioinformatics 2020 Jun;2(2):lqaa029. https://doi.org/10.1093/nargab/lqaa029.
33. Quinn TP, Erb I. Interpretable Log Contrasts for the Classification of Health Biomarkers: a New Approach to Balance Selection. mSystems 2020 Apr;5(2):10.1128/msystems.00230–19. https://journals.asm.org/doi/10.1128/msystems.00230-19.
34. Wang XW, Liu YY. Comparative study of classifiers for human microbiome data. Medicine in Microecology 2020 Jun;4:100013. https://www.sciencedirect.com/science/article/pii/S2590097820300100.
35. Karwowska Z, Aasmets O, research team EB, Kosciolek T, Org E. Effects of data transformation and model selection on feature importance in microbiome classification data. Microbiome 2025;13(1):2.
36. Giliberti R, Cavaliere S, Mauriello IE, Ercolini D, Pasolli E. Host phenotype classification from human microbiome data is mainly driven by the presence of microbial taxa. PLoS Computational Biology 2022;18(4):e1010066.
37. Miller EA, Beasley DE, Dunn RR, Archie EA. Lactobacilli Dominance and Vaginal pH: Why Is the Human Vaginal Microbiome Unique? Frontiers in Microbiology 2016;7:1936.
38. Lin H, Peddada SD. Analysis of microbial compositions: a review of normalization and differential abundance analysis. NPJ Biofilms and Microbiomes 2020 Dec;6:60. https://www.ncbi.nlm.nih.gov/pmc/articles/PMC7710733/.
39. Li Q, Yu Y, Kossinna P, Lun T, Liao W, Zhang Q. XA4C: eXplainable representation learning via Autoencoders revealing Critical genes. PLOS Computational Biology 2023 Oct;19(10):e1011476. https://journals.plos.org/ploscompbiol/article?id=10.1371/journal.pcbi.1011476.
40. Vivek S, Faul J, Thyagarajan B, Guan W. Explainable variational autoencoder (E-VAE) model using genome-wide SNPs to predict dementia. Journal of Biomedical Informatics 2023 Dec;148:104536. https://www.sciencedirect.com/science/article/pii/S1532046423002575.
41. Ali S, Abuhmed T, El-Sappagh S, Muhammad K, Alonso-Moral JM, Confalonieri R, et al. Explainable Artificial Intelligence (XAI): What we know and what is left to attain Trustworthy Artificial Intelligence. Information Fusion 2023 Nov;99:101805. https://www.sciencedirect.com/science/article/pii/S1566253523001148.
42. Hacılar H, Nalbantoğlu OU, Bakir-Güngör B. Machine Learning Analysis of Inflammatory Bowel Disease-Associated Metagenomics Dataset. In: 2018 3rd International Conference on Computer Science and Engineering (UBMK); 2018. p. 434–438. https://ieeexplore.ieee.org/document/8566487/?arnumber=8566487.
43. Fukui H, Nishida A, Matsuda S, Kira F, Watanabe S, Kuriyama M, et al. Usefulness of Machine Learning-Based Gut Microbiome Analysis for Identifying Patients with Irritable Bowels Syndrome. Journal of Clinical Medicine 2020 Aug;9(8):2403. https://www.mdpi.com/2077-0383/9/8/2403.
44. Johnson JS, Spakowicz DJ, Hong BY, Petersen LM, Demkowicz P, Chen L, et al. Evaluation of 16S rRNA gene sequencing for species and strain-level microbiome analysis. Nature Communications 2019 Nov;10(1):5029. https://www.nature.com/articles/s41467-019-13036-1.
45. Chakravorty S, Helb D, Burday M, Connell N, Alland D. A detailed analysis of 16S ribosomal RNA gene segments for the diagnosis of pathogenic bacteria. Journal of microbiological methods 2007 Feb;69(2):330. https://pmc.ncbi.nlm.nih.gov/articles/PMC2562909/.
46. Amplicon Sequencing Solutions;. https://emea.illumina.com/techniques/sequencing/dna-sequencing/targeted-resequencing/amplicon-sequencing.html, accessed

19 December 2024.
47. Rothberg JM, Leamon JH. The development and impact of 454 sequencing. Nature Biotechnology 2008 Oct;26(10):1117–1124.
48. Nguyen NP, Warnow T, Pop M, White B. A perspective on 16S rRNA operational taxonomic unit clustering using sequence similarity. npj Biofilms and Microbiomes 2016 Apr;2(1):1–8. https://www.nature.com/articles/npjbiofilms20164.
49. Callahan BJ, McMurdie PJ, Holmes SP. Exact sequence variants should replace operational taxonomic units in marker-gene data analysis. The ISME Journal 2017 Jul;11(12):2639. https://pmc.ncbi.nlm.nih.gov/articles/PMC5702726/.
50. Santamaria M, Fosso B, Consiglio A, De Caro G, Grillo G, Licciulli F, et al. Reference databases for taxonomic assignment in metagenomics. Briefings in Bioinformatics 2012 Nov;13(6):682–695. https://doi.org/10.1093/bib/bbs036.
51. Aitchison J. The Statistical Analysis of Compositional Data. London: Chapman and Hall.; 1986.
52. Law CW, Chen Y, Shi W, Smyth GK. voom: precision weights unlock linear model analysis tools for RNA-seq read counts. Genome Biology 2014 Feb;15(2):R29. https://doi.org/10.1186/gb-2014-15-2-r29.
53. McMurdie PJ, Holmes S. Waste not, want not: why rarefying microbiome data is inadmissible. PLoS Computational Biology 2014;10(4):e1003531.
54. Guyon I, Elisseeff A. An introduction to variable and feature selection. J Mach Learn Res 2003 Mar;3(null):1157–1182.
55. Roy G, Prifti E, Belda E, Zucker JD. Deep learning methods in metagenomics: a review. Microbial Genomics 2024;10(4):001231. https://www.microbiologyresearch.org/content/journal/mgen/10.1099/mgen.0.001231.
56. Cover TM, Thomas JA. Elements of Information Theory. John Wiley & Sons; 2012.
57. Ding C, Peng H. Minimum redundancy feature selection from microarray gene expression data. In: Computational Systems Bioinformatics. CSB2003. Proceedings of the 2003 IEEE Bioinformatics Conference. CSB2003 Stanford, CA, USA: IEEE Comput. Soc; 2003. p. 523–528. http://ieeexplore.ieee.org/document/1227396/.
58. Kraskov A, Stögbauer H, Grassberger P. Estimating mutual information. Physical Review E, Statistical, Nonlinear, and Soft Matter Physics 2004 Jun;69(6 Pt 2):066138.
59. smazzanti, smazzanti/mrmr minimum Redundancy - Maximum Relevance; 2025. https://github.com/smazzanti/mrmr, installed 1 November 2024.
60. Kononenko I. Estimating attributes: Analysis and extensions of RELIEF. In: Bergadano F, De Raedt L, editors. Machine Learning: ECML-94 Berlin, Heidelberg: Springer; 1994. p. 171–182.
61. Urbanowicz RJ, Meeker M, La Cava W, Olson RS, Moore JH. Relief-based feature selection: Introduction and review. Journal of Biomedical Informatics 2018 Sep;85:189–203. https://www.sciencedirect.com/science/article/pii/S1532046418301400.
62. Wickramaratne D, Wijesinghe R, Weerasinghe R. Human Gut Microbiome Data Analysis for Disease Likelihood Prediction Using Autoencoders. In: 2021 21st International Conference on Advances in ICT for Emerging Regions (ICter); 2021. p. 49–54. https://ieeexplore.ieee.org/document/9774811/?arnumber=9774811, iSSN: 2472-7598.
63. Oh M, Zhang L. DeepMicro: deep representation learning for disease prediction based on microbiome data. Scientific Reports 2020 Apr;10(1):6026. https://www.nature.com/articles/s41598-020-63159-5.
64. Queen O, Emrich SJ. LASSO-based feature selection for improved microbial and microbiome classification. In: 2021 IEEE International Conference on Bioinformatics and Biomedicine (BIBM); 2021. p. 2301–2308. https://ieeexplore.ieee.org/document/9669485.
65. Breiman L. Random Forests. Machine Learning 2001;45(1):5–32.
66. Cawley GC, Talbot NLC. On Over-fitting in Model Selection and Subsequent Selection Bias in Performance Evaluation. Journal of Machine Learning Research 2010;11(70):2079–2107. http://jmlr.org/papers/v11/cawley10a.html.
67. Hastie T, Tibshirani R, Friedman J. The Elements of Statistical Learning: Data Mining, Inference, and Prediction. 2nd ed. Springer; 2009. Chapter 3: Linear Methods for Regression.
68. Cortes C, Vapnik V. Support-vector networks. Machine Learning 1995;20(3):273–297.
69. Park J, Sandberg IW. Universal approximation using radial-basis-function networks. Neural computation 1991;3(2):246–257.
70. Chen T, Guestrin C. Xgboost: A scalable tree boosting system. In: Proceedings of the 22nd acm sigkdd international conference on knowledge discovery and data mining; 2016. p. 785–794.
71. Cover T, Hart P. Nearest neighbor pattern classification. IEEE Transactions on Information Theory 1967;13(1):21–27.
72. Demšar J. Statistical Comparisons of Classifiers over Multiple Data Sets. Journal of Machine Learning Research 2006;7(1):1–30. http://jmlr.org/papers/v7/demsar06a.html.
73. García S, Fernández A, Luengo J, Herrera F. Advanced nonparametric tests for multiple comparisons in the design of experiments in computational intelligence and data mining: Experimental analysis of power. Information Sciences 2010 May;180(10):2044–2064. https://www.sciencedirect.com/science/article/pii/S0020025509005404.
74. Rodríguez-Fdez I, Canosa A, Mucientes M, Bugarín A. STAC: A web platform for the comparison of algorithms using statistical tests. In: 2015 IEEE International Conference on Fuzzy Systems (FUZZ-IEEE); 2015. p. 1–8. https://ieeexplore.ieee.org/document/7337889.
75. Garach I. Exploring the role of normalization and feature selection in microbiome disease classification pipelines 2025;https://workflowhub.eu/workflows/1807?version=1.
76. OpenAI, ChatGpt (GPT-4 Turbo, November 2023)[Large language model]; 2024. chat.openai.com/chat, queried until 16/02/2025.

Click here to access/download
Supplementary Material
Revised Supplementary.pdf

Response to Reviewers Click here to access/download;Personal Cover;Answers to Reviewer Comments.pdf

Response to the reviewers

We thank the reviewers for their constructive comments. We have addressed all of them and modified the paper accordingly. Our detailed answers follow.

Please note that reviewers' comments are in bold while our answers are not. Additions to the original manuscript are indicated in blue.

Reviewer 1

1) Are the methods appropriate to the aims of the study, are they well described, and are necessary controls included? Yes, the study aims to compare biomarker discovery in 16S microbiome disease classification. They particularly look at various normalization and feature selection processes across different classification algorithms. They include controls of testing the models without normalization as a baseline.

2) Are the conclusions adequately supported by the data shown? Yes, the data is clearly and professionally presented in the paper along with the author's conclusions on how future work should approach selecting the appropritate normalization, feature selection, or even algorithm for classification.

3) Please indicate the quality of language in the manuscript. Does it require a heavy editing for language and clarity? The manuscript is of high quality. The sections are clear, appropriate data, tests, and analysis are provided. There are little if any language errors. My small suggestion would be to add numbers to the section and subsection seperators.

4) Are you able to assess all statistics in the manuscript, including the appropriateness of statistical tests used? Yes, again the data is clearly and professionally presented.

Reply: We thank the reviewer for their positive and constructive feedback. We agree that numbering the sections and subsections could improve navigability and clarity. However, we have followed the official Overleaf template provided for this journal, and upon reviewing published articles, we noticed that section numbering is generally not used. Nevertheless, we extend this recommendation to the editor and are happy to implement section numbering if deemed appropriate.

Reviewer Point P 1.1 — My number one issue with the paper is it would be significantly better if the authors provided more novelty. As it is, the paper needs benchmarking with more existing methods.

Reply: We thank the reviewer for raising this important point regarding the novelty and methodological breadth of our study. In response, we have expanded the benchmarking to include additional normalization techniques—specifically, log-ratio abundance (logRA) and presence/absence (PA)—in combination with all classifiers and feature selection methods. We also incorporated the consideration of rarefaction prior to normalization for baseline classification, further enriching the comparative analysis.

Furthermore, we included Random Forest Feature Importances as an additional feature selection method, allowing for the evaluation of a different feature selection approach and its interactions within the different steps of our pipeline.

The results of the new methods included in the benchmarking are presented on pages 3 to 6. Moreover, they are discussed mainly on page 9.

We fully agree with the reviewer that there is potential for incorporating more complex methodologies (such as wrapper-based feature selection or optimization techniques using evolutionary algorithms). In fact, we explicitly acknowledge this in the potential implications section, where we highlight such approaches as promising future directions. However, in the present work, we intentionally focused on a set of methods that are widely used, computationally tractable, and amenable to extensive validation across a large number of datasets. Our goal was to provide a comprehensive and reproducible comparison across normalization, feature selection, and classification steps, laying the groundwork for future investigations involving more advanced and computationally intensive techniques.

We believe that the additions made in this revised version considerably enhance the novelty and completeness of our study, and we appreciate the reviewer's suggestion to further strengthen its contribution.

Reviewer 2

This manuscript addresses an important and timely topic concerning the impact of data transformation and feature selection techniques on machine learning classification of microbiome datasets. Given the growing interest in developing robust predictive models in microbiome research, especially for disease classification, this study is of high relevance. The paper is generally well-written and provides a comprehensive overview of commonly used preprocessing and modeling methods in the microbiome field. However, the manuscript would benefit from several substantial revisions to improve clarity, interpretability, and completeness:

Reply: Thank you for your constructive and helpful feedback on our manuscript. We appreciate the time and effort you have dedicated to providing such a thorough review.

We have carefully considered all the points raised and have made substantial revisions. Below, we provide a point-by-point response to each of your comments, detailing the changes we have implemented.

Reviewer Point P 2.1 — Inclusion of Additional Transformations: The manuscript should include results using log-relative abundance (log-RelAb) and presence/absence (PA) transformations, as these are standard approaches in microbiome data preprocessing and their inclusion would improve the comprehensiveness of the analysis. Additionally, it would be valuable to evaluate the effect of rarefaction on model performance. Including rarefied datasets would allow the authors to assess how this commonly used normalization method influences classification outcomes and whether it interacts with transformation or feature selection strategies.

Reply: We thank the reviewer for this excellent and constructive suggestion. In response, we expanded our study to include two additional normalization strategies: log-relative abundance and presence/absence (PA). The log-relative abundance transformation is now referred to as logRA throughout the manuscript for consistency. Both transformations were incorporated into the baseline classification analysis as well as the full set of pipeline comparisons across all feature selection methods and classifiers. The inclusion of the presence/absence (PA) transformation, in particular, provided valuable insight and contributed significantly to our comprehension of the nature of the problem and to the completeness of our analysis. We are especially grateful for this recommendation, which led to meaningful improvements in the overall quality and depth of the study. Results of baseline classification with the additional proposed normalizations are presented on pages 3 to 5. LogRA and PA results sections read as:

"Finally, focusing on data normalized using the remaining two transformations, log-transformed Relative Abundances (logRA) and Presence-Absence (PA), the Friedman test also revealed significant differences between classifiers in both cases . For the logRA normalization, results were extremely similar to those from CLR. Logistic Regression models achieved the best average ranks, followed by Support Vector Machines and Boosting. Unlike what was observed with CLR normalization, in this case Boosting models did not show statistically significant differences in performance compared to Logistic Regression and SVM. Random Forest and KNN fell behind, being significantly outperformed by the top models.

In contrast with abundance-based transformations, the PA normalization showed higher performance in a larger range of different models, with Logistic Regression, SVM and Random Forest ranking high

and with no significative statistical differences between them. However, the Boosting and KNN results were not comparable to the top ones, even though KNN worked better compared with the other normalizations.

On average, best performing models were Random Forest for RA data and Logistic Regression for CLR and logRA normalizations. PA obtained similar performance to the best abundance-based pipelines when using Random Forest or Logistic Regression classifier (see again AUC boxplots at figure 2 and Finner's test results at figure 3)" We thank the reviewer for the suggestion on assessing rarefaction.

In response, we conducted an additional analysis to assess the impact of rarefaction on classification performance across normalization methods. Two rarefaction strategies were evaluated: (i) standard rarefaction to the minimum library size, and (ii) a modified approach using the 5th percentile of sequencing depths to mitigate information loss from very shallow samples. These results are now included at the end of the Baseline classification section with tests and figures in the Supplementary material. Results showed that rarefaction consistently did not provide performance benefits and, in some cases, led to substantial degradation in accuracy. As such, rarefaction was not applied in subsequent pipeline analyses involving feature selection. Rarefaction analysis section is located at the end of page 4 of the manuscript and reads as:

"An additional analysis was conducted to evaluate the impact of rarefaction and its interactions with normalization methods on classification performance. Rarefaction involves subsampling the count data to a uniform sequencing depth across samples. This is typically done using the minimum library size observed in the dataset. However, when certain samples are extremely shallow, this approach can result in a substantial loss of information. To overcome this, we included a second variant where the rarefaction depth was set to the 5th percentile of each dataset sample depths, removing samples with less reads than this quantity.

In the global comparison, Wilcoxon paired tests revealed statistically significant differences between non-rarefied data and both rarefaction approaches, with all p-values indicating strong significance in detriment of rarefaction (specifically, $p = 4.4 \cdot 10^{-13}$ for standard rarefaction and $p = 9.3 \cdot 10^{-5}$ for Q05 rarefaction). Quantile-modified rarefaction also performed significantly better than standard rarefaction $p = 1.5 \cdot 10^{-9}$.

When examining each normalization method individually, consistent patterns can be observed (see Supplementary Figure S1). For RA and CLR normalization, non-rarefied data significantly outperformed both rarefied counterparts. In contrast, no differences were found for logRA and PA normalization for quantile-based rarefaction, though it still did not yield any improvements. Interestingly, for CLR, logRA, and PA, the quantile-based rarefaction yielded significantly better results than standard rarefaction (see Supplementary Table T2). Therefore, rarefaction was not applied in the subsequent pipeline analyses involving feature selection, as it consistently led to either inferior or non-improved results across normalization methods."

We also mentioned them at the global discussion on page 9 and rigorously modified the sections Background and Methods to include the new techniques considered.

Reviewer Point P 2.2 — Simplify Figure 1 for Clarity: Figure 1 would be more effective if it presented a single boxplot per transformation method (e.g., CLR, RELAB) to facilitate simpler and more direct comparisons.

Reply: We have followed the reviewer's suggestion and created a new figure showing a single boxplot for each transformation method to facilitate a straightforward comparison. We have incorporated this

as a key part of a revised and consolidated Figure 2 (Figure 1 in the first version of the MS). To provide a complete picture that supports the detailed analysis in the text, this new figure now also includes the breakdown per normalization method integrated into a single, cohesive panel. It can be seen now at the bottom of page 3.

We believe this revised figure successfully addresses your concern for simplicity while retaining the necessary detail discussed throughout the manuscript.

Reviewer Point P 2.3 — Improve Table 8 Presentation: Table 8 lacks sufficient description. Consider either improving the accompanying explanation or replacing it with a figure (e.g., boxplots) to visually highlight performance distributions. If a table is retained, bold the best-performing results for clarity.

Reply: We appreciate the reviewer's valuable feedback regarding the presentation of Table 8 (now Table 7). We have thoroughly revised the table to enhance its clarity and informativeness, addressing all your suggestions.

We have significantly improved the accompanying description to provide a more comprehensive explanation of its content. Furthermore, we have restructured the table to clearly delineate the different aspects of the results. On the left side, we now present the baseline classification performance using all features. This is followed by the performance of the top-performing feature selection methods, each explicitly paired with its corresponding percentage of feature reduction. As suggested, the best-performing results for each dataset are now clearly bolded, making it much easier for the reader to identify optimal outcomes. It can be seen now at the top of page 10.

Reviewer Point P 2.4 — Unify Boxplots in Figure 5: For better comparison, combine all boxplots in Figure 5 into a single panel and ensure the Y-axis scale is consistent across plots.

Reply: We thank the reviewer for this helpful suggestion. In the revised manuscript, we have addressed the Y-axis consistency issue and updated the figure accordingly. The figure referenced is now Figure 4. While we understand the value of combining all boxplots into a single panel for comparison, we decided to maintain separate panels to clearly distinguish between comparisons and avoid visual clutter. It can be seen now at the bottom of page 4.

Reviewer Point P 2.5 — Format P-values Appropriately: Round p-values to a reasonable number of decimal places (e.g., three significant digits) for readability.

Reply: We thank the reviewer for this observation. In our manuscript, we have now formatted p-values consistently using scientific notation or fixed-point notation with two significant figures, which we believe offers a balance between readability and precision. For very small p-values, we intentionally used scientific notation to ensure that their order of magnitude remains visible, which is important for displaying the strength of statistical evidence. This formatting choice is in line with other studies in the same field. Nevertheless, if the reviewer or editor considers a different notation more appropriate, we would be happy to adapt accordingly.

Reviewer Point P 2.6 — Enhance Figure Quality: Improve the resolution of all figures and ensure consistent text formatting across all graphical elements.

Reply: We thank the reviewer for this suggestion. We have addressed this point by combining some of the figures for clarity and overall improving the resolution of all figures. We have also ensured consistent text formatting across all graphical elements. We believe these changes enhance the visual quality and readability of the figures now numbered as 2, 3, 4, 7, 8, 9, 11 and 12.

Reviewer Point P 2.7 — Revise Figure 9 Visualization: Instead of displaying a small plot, use a line plot with a shaded area representing standard deviation or confidence intervals to better convey variability.

Reply: We thank the reviewer for the helpful suggestion. In response, we have revised Figure 9 by replacing the previous corner plot with a line plot that includes shaded areas representing the standard deviation, thus providing a clearer visualization of variability across the data. We have also enhanced the overall quality of the figure. It can be seen now at the bottom of page 7.

Reviewer Point P 2.8 — Terminology Refinement: Avoid using the term "biomarker" unless biologically validated. "Most important features" or "predictive features" would be more appropriate in the context of machine learning feature selection.

Reply: We thank the reviewer for the valuable feedback regarding the use of the term "biomarker". We agree and we have implemented the suggested changes throughout the manuscript. Specifically, we have replaced "biomarker" with more appropriate terms such as "predictive features," "microbial features,", "key attributes" and "discriminative features" in the context of our machine learning feature selection. We have only retained the term "biomarker" in general sections, such as the Introduction, Methods, Potential Implications and Conclusions, where we discuss the broader state of the art and the potential for selected features to be biologically or externally validated as true biomarkers in future research. This ensures that our terminology remains precise and contextually accurate.

Reviewer Point P 2.9 — Relocate Table 7 Content: The discussion of specific taxa and Table 7 should be moved into the Results section, as it presents findings rather than interpretation.

Reply: We thank the reviewer observation. We have moved the content of Table 7 into the Analyses section (now table 5 at the top of page 8), where we now present the selected taxa and highlight the high genera variability observed in the results. However, we kept the interpretation and comparison with previously reported associations in the literature in the Discussion section, as we believe this context helps clarify the relevance of the findings.

Reviewer Point P 2.10 — Reorganize the Discussion Section: A significant portion of the current discussion reads as results. These subsections should be moved to the Results section, and the Discussion should be condensed to focus on interpretation, implications, and broader context.

Reply: We appreciate the reviewer's observation. In response, we have reorganized and condensed the Discussion section to avoid repeating points already presented in the Analyses section. We now focus more on interpreting the findings, exploring possible reasons behind the observed behavior of the methods, and situating our results within the context of relevant literature. These changes aim to enhance the clarity and depth of the discussion.

Reviewer Point P 2.11 — Add a Study Design Schematic: A clear, concise diagram summarizing the study design, including data processing, transformation, modeling, and evaluation steps, would greatly enhance reader comprehension.

Reply: We thank the reviewer for the suggestion. We have incorporated a graphical abstract into our submission, which illustrates the key steps of our study design, including data processing, normalization, feature selection, classification, and evaluation. We believe this addition significantly enhances the clarity and comprehension for the reader.

Reviewer Point P 2.12 — Summarize Feature Importance Methods: Consider summarizing the feature importance techniques in a dedicated table to facilitate comparison and improve readability.

Reply:

We thank the reviewer for this suggestion. We have incorporated a dedicated table (Table 8 at the top of page 12) summarizing the feature importance techniques to provide readers with a quick yet informative overview of the methods.

Reviewer Point P 2.13 — Expand Method Descriptions: Briefly introduce each classification method (e.g., logistic regression, random forest, SVM) in the main text and provide a more detailed description in the Methods section.

Reply: We appreciate rhe reviewer's valuable comment. We have now addressed this by briefly introducing the classifiers in the main text, as suggested.

"These models represent a diverse set of classification strategies. Random Forest and XGBoost are tree-based ensemble methods known for their robustness and ability to capture complex non-linear relationships. Logistic Regression is a linear model that provides interpretable coefficients and performs well when the data is linearly separable. Support Vector Machine is a margin-based classifier effective in high-dimensional spaces, particularly suitable for sparse data. Finally, K-Nearest Neighbor is a lazy learning method that classifies samples based on similarity to their neighbors, though it can be sensitive to noise and sparsity in the data. A more detailed description of each classifier is provided in the Methods section."

Furthermore, we have dedicated subsections within the Methods section to provide a more in-depth description of each classifier, ensuring a comprehensive understanding for the reader.

In my opinion, this paper is an important contribution to the machine learning community working with microbiome data. While the manuscript could benefit from improvements in clarity and readability, it holds significant value. The feature selection aspect is particularly strong and stands out as a major strength.

Click here to access/download;Graphical Abstract;GraphicalAbstract.png

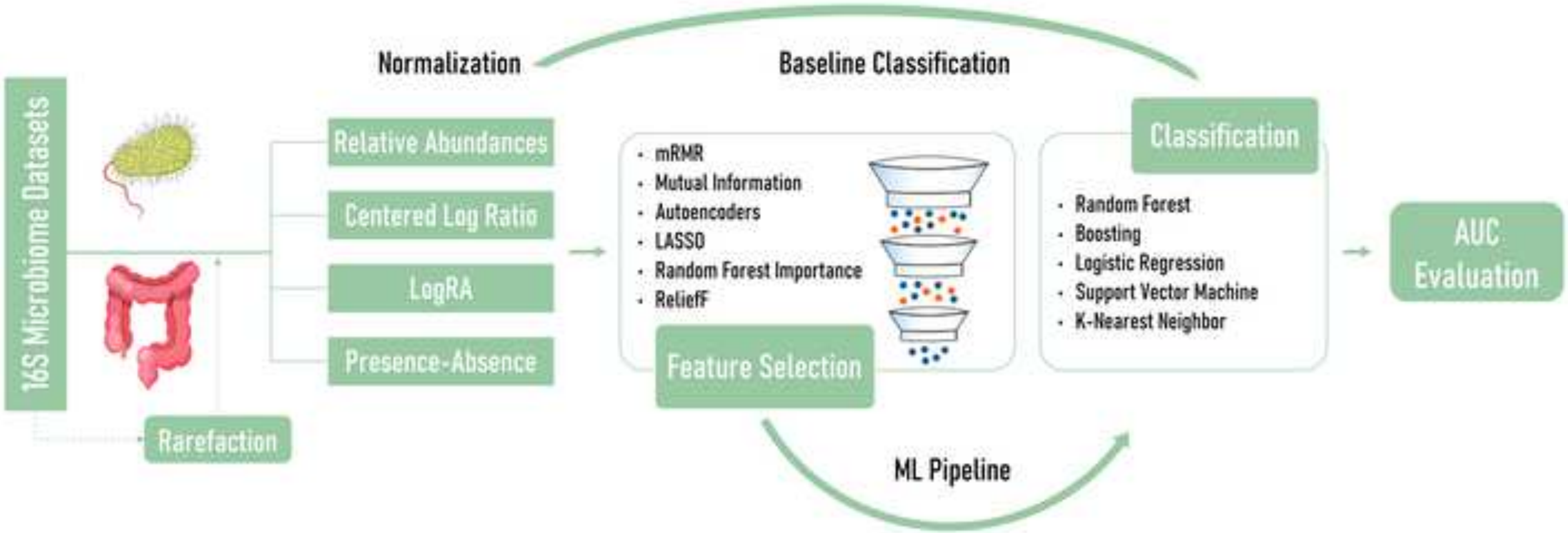

Supplement: giaf096_GIGA-D-25-00079_Revision_1 [file giaf096_giga-d-25-00079_revision_1.pdf]
