# Supplementary material for: Exploring the role of normalization and feature selection in microbiome disease classification pipelines
Source: Gigascience. 2025 Sep 2;14:giaf096. doi: 10.1093/gigascience/giaf096 (PMC12402773; doi:10.1093/gigascience/giaf096)
Supplement: giaf096_Authors_Response_To_Reviewer_Comments_Original_Submission [file giaf096_authors_response_to_reviewer_comments_original_submission.pdf]

Author's Response To Reviewer Comments

Response to editor:

In addition, please register 16SMicrobiomeMLFS in the bio.tools and SciCrunch.org databases to receive RRID (Research Resource Identification Initiative ID) and biotoolsID identifiers, and include these in your manuscript. Computational workflows should be registered in workflowhub.eu and the DOIs cited in the relevant places in the manuscript. These will facilitate tracking, reproducibility and re-use of your tool.

We thank the editor's feedback. Code and workflow has been submited to these platforms and is cited at the "Availability of source code and requirements" section.

Response to the reviewers (Latex Formated Document submited with these answer to enable easier review).

We thank the reviewers for their constructive comments. We have addressed all of them and modified the paper accordingly. Our detailed answers follow.
Please note that reviewers' comments are in bold while our answers are not. Additions to the original manuscript are indicated in blue at the revised manuscript.

Reviewer 1

1) Are the methods appropriate to the aims of the study, are they well described, and are necessary controls included? Yes, the study aims to compare biomarker discovery in 16S microbiome disease classification. They particularly look at various normalization and feature selection processes across different classification algorithms. They include controls of testing the models without normalization as a baseline.
2) Are the conclusions adequately supported by the data shown? Yes, the data is clearly and professionally presented in the paper along with the author's conclusions on how future work should approach selecting the appropritate normalization, feature selection, or even algorithm for classification.
3) Please indicate the quality of language in the manuscript. Does it require a heavy editing for language and clarity? The manuscript is of high quality. The sections are clear, appropriate data, tests, and analysis are provided. There are little if any language errors. My small suggestion would be to add numbers to the section and subsection seperators.
4) Are you able to assess all statistics in the manuscript, including the appropriateness of statistical tests used? Yes, again the data is clearly and professionally presented.

Reply: We thank the reviewer for their positive and constructive feedback. We agree that numbering the sections and subsections could improve navigability and clarity. However, we have followed the official Overleaf template provided for this journal, and upon reviewing published articles, we noticed that section numbering is generally not used. Nevertheless, we extend this recommendation to the editor and are happy to implement section numbering if deemed appropriate.

Reviewer Point P1.1 — My number one issue with the paper is it would be significantly better if the authors provided more novelty. As it is, the paper needs benchmarking with more existing methods.

Reply: We thank the reviewer for raising this important point regarding the novelty and methodological breadth of our study. In response, we have expanded the benchmarking to include additional normalization techniques—specifically, log-ratio abundance (logRA) and presence/absence (PA)—in combination with all classifiers and feature selection methods. We also incorporated the consideration of rarefaction prior to normalization for baseline classification, further enriching the comparative analysis.
Furthermore, we included Random Forest Feature Importances as an additional feature selection method, allowing for the evaluation of a different feature selection approach and its interactions within the different steps of our pipeline.

The results of the new methods included in the benchmarking are presented on pages 3 to 6.
Moreover, they are discussed mainly on page 9.

We fully agree with the reviewer that there is potential for incorporating more complex methodologies (such as wrapper-based feature selection or optimization techniques using evolutionary algorithms).
In fact, we explicitly acknowledge this in the potential implications section, where we highlight such approaches as promising future directions. However, in the present work, we intentionally focused on a set of methods that are widely used, computationally tractable, and amenable to extensive validation across a large number of datasets. Our goal was to provide a comprehensive and reproducible comparison across normalization, feature selection, and classification steps, laying the groundwork for future investigations involving more advanced and computationally intensive techniques.
We believe that the additions made in this revised version considerably enhance the novelty and completeness of our study, and we appreciate the reviewer's suggestion to further strengthen its contribution.

Reviewer 2

This manuscript addresses an important and timely topic concerning the impact of data transformation and feature selection techniques on machine learning classification of microbiome datasets. Given the growing interest in developing robust predictive models in microbiome research, especially for disease classification, this study is of high relevance. The paper is generally well-written and provides a comprehensive overview of commonly used preprocessing and modeling methods in the microbiome field. However, the manuscript would benefit from several substantial revisions to improve clarity, interpretability, and completeness:

Reply: Thank you for your constructive and helpful feedback on our manuscript. We appreciate the time and effort you have dedicated to providing such a thorough review. We have carefully considered all the points raised and have made substantial revisions. Below, we provide a point-by-point response to each of your comments, detailing the changes we have implemented.

Reviewer Point P2.1 — Inclusion of Additional Transformations: The manuscript should include results using log-relative abundance (log-RelAb) and presence/absence (PA) transformations, as these are standard approaches in microbiome data preprocessing
and their inclusion would improve the comprehensiveness of the analysis. Additionally, it would be valuable to evaluate the effect of rarefaction on model performance. Including rarefied datasets would allow the authors to assess how this commonly used normalization method influences classification outcomes and whether it interacts with transformation or feature selection strategies.

Reply: We thank the reviewer for this excellent and constructive suggestion. In response, we expanded our study to include two additional normalization strategies: log-relative abundance and presence/absence (PA). The log-relative abundance transformation is now referred to as logRA throughout the manuscript for consistency. Both transformations were incorporated into the baseline classification analysis as well as the full set of pipeline comparisons across all feature selection methods and classifiers. The inclusion of the presence/absence (PA) transformation, in particular, provided valuable insight and contributed significantly to our comprehension of the nature of the problem and to the completeness of our analysis. We are especially grateful for this recommendation, which led to meaningful improvements in the overall quality and depth of the study. Results of baseline classification with the additional proposed normalizations are presented on pages 3 to 5.

LogRA and PA results sections read as:

"Finally, focusing on data normalized using the remaining two transformations, log-transformed Relative Abundances (logRA) and Presence-Absence (PA), the Friedman test also revealed significant differences between classifiers in both cases . For the logRA normalization, results were extremely similar to those from CLR. Logistic Regression models achieved the best average ranks, followed by Support Vector Machines and Boosting. Unlike what was observed with CLR normalization, in this case Boosting
models did not show statistically significant differences in performance compared to Logistic Regression

and SVM. Random Forest and KNN fell behind, being significantly outperformed by the top models. In contrast with abundance-based transformations, the PA normalization showed higher performance in a larger range of different models, with Logistic Regression, SVM and Random Forest ranking high and with no significative statistical differences between them. However, the Boosting and KNN results were not comparable to the top ones, even though KNN worked better compared with the other normalizations.
On average, best performing models were Random Forest for RA data and Logistic Regression for CLR and logRA normalizations. PA obtained similar performance to the best abundance-based pipelines when using Random Forest or Logistic Regression classifier (see again AUC boxplots at figure 2 and Finner's test results at figure 3)"

We thank the reviewer for the suggestion on assessing rarefaction. In response, we conducted an additional analysis to assess the impact of rarefaction on classification performance across normalization methods. Two rarefaction strategies were evaluated: (i) standard rarefaction to the minimum library size, and (ii) a modified approach using the 5th percentile of sequencing depths to mitigate information loss from very shallow samples. These results are now included at the end of the Baseline classification section with tests and figures in the Supplementary material. Results showed that rarefaction consistently did not provide performance benefits and, in some cases, led to substantial degradation in accuracy. As such, rarefaction was not applied in subsequent pipeline analyses involving feature selection. Rarefaction analysis section is located at the end of page 4 of the manuscript and reads as:

"An additional analysis was conducted to evaluate the impact of rarefaction and its interactions with normalization methods on classification performance. Rarefaction involves subsampling the count data to a uniform sequencing depth across samples. This is typically done using the minimum library size observed in the dataset. However, when certain samples are extremely shallow, this approach can result in a substantial loss of information. To overcome this, we included a second variant where the rarefaction depth was set to the 5th percentile of each dataset sample depths, removing samples with less reads than this quantity. In the global comparison, Wilcoxon paired tests revealed statistically significant differences between non-rarefied data and both rarefaction approaches, with all p-values indicating strong significance in detriment of rarefaction (specifically, $p = 4.4 \cdot 10{-}13$ for standard rarefaction and $p = 9.3 \cdot 10{-}5$ for Q05 rarefaction). Quantile-modified rarefaction also performed significantly better than standard rarefaction $p = 1.5 \cdot 10{-}9$.
When examining each normalization method individually, consistent patterns can be observed (see Supplementary Figure S1). For RA and CLR normalization, non-rarefied data significantly outperformed both rarefied counterparts. In contrast, no differences were found for logRA and PA normalization for quantile-based rarefaction, though it still did not yield any improvements. Interestingly, for CLR, logRA, and PA, the quantile-based rarefaction yielded significantly better results than standard rarefaction (see Supplementary Table T2). Therefore, rarefaction was not applied in the subsequent pipeline analyses involving feature selection, as it consistently led to either inferior or non-improved results across normalization methods."

We also mentioned them at the global discussion on page 9 and rigorously modified the sections Background and Methods to include the new techniques considered.

Reviewer Point P2.2 — Simplify Figure 1 for Clarity: Figure 1 would be more effective if it presented a single boxplot per transformation method (e.g., CLR, RELAB) to facilitate simpler and more direct comparisons.

Reply: We have followed the reviewer's suggestion and created a new figure showing a single boxplot for each transformation method to facilitate a straightforward comparison. We have incorporated this as a key part of a revised and consolidated Figure 2 (Figure 1 in the first version of the MS). To provide a complete picture that supports the detailed analysis in the text, this new figure now also includes the breakdown per normalization method integrated into a single, cohesive panel. It can be seen now at the bottom of page 3. We believe this revised figure successfully addresses your concern for simplicity while retaining the necessary detail discussed throughout the manuscript.

Reviewer Point P2.3 — Improve Table 8 Presentation: Table 8 lacks sufficient description. Consider either improving the accompanying explanation or replacing it with a figure (e.g., boxplots) to visually highlight performance distributions. If a table is retained, bold the best-performing results for clarity.

Reply: We appreciate the reviewer's valuable feedback regarding the presentation of Table 8 (now Table 7). We have thoroughly revised the table to enhance its clarity and informativeness, addressing all your suggestions.
We have significantly improved the accompanying description to provide a more comprehensive explanation of its content. Furthermore, we have restructured the table to clearly delineate the different aspects of the results. On the left side, we now present the baseline classification performance using all features. This is followed by the performance of the top-performing feature selection methods, each explicitly paired with its corresponding percentage of feature reduction. As suggested, the bestperforming results for each dataset are now clearly bolded, making it much easier for the reader to identify optimal outcomes. It can be seen now at the top of page 10.

Reviewer Point P2.4 — Unify Boxplots in Figure 5: For better comparison, combine all boxplots in Figure 5 into a single panel and ensure the Y-axis scale is consistent across plots.

Reply: We thank the reviewer for this helpful suggestion. In the revised manuscript, we have addressed the Y-axis consistency issue and updated the figure accordingly. The figure referenced is now Figure 4. While we understand the value of combining all boxplots into a single panel for comparison, we decided to maintain separate panels to clearly distinguish between comparisons and avoid visual clutter. It can be seen now at the bottom of page 4.

Reviewer Point P2.5 — Format P-values Appropriately: Round p-values to a reasonable number of decimal places (e.g., three significant digits) for readability.

Reply: We thank the reviewer for this observation. In our manuscript, we have now formatted pvalues consistently using scientific notation or fixed-point notation with two significant figures, which we believe offers a balance between readability and precision. For very small p-values, we intentionally used scientific notation to ensure that their order of magnitude remains visible, which is important for displaying the strength of statistical evidence. This formatting choice is in line with other studies in the same field. Nevertheless, if the reviewer or editor considers a different notation more appropriate, we would be happy to adapt accordingly.

Reviewer Point P2.6 — Enhance Figure Quality: Improve the resolution of all figures and ensure consistent text formatting across all graphical elements.

Reply: We thank the reviewer for this suggestion. We have addressed this point by combining some of the figures for clarity and overall improving the resolution of all figures. We have also ensured consistent text formatting across all graphical elements. We believe these changes enhance the visual quality and readability of the figures now numbered as 2, 3, 4, 7, 8, 9, 11 and 12.

Reviewer Point P2.7 — Revise Figure 9 Visualization: Instead of displaying a small plot, use a line plot with a shaded area representing standard deviation or confidence intervals to better convey variability.

Reply: We thank the reviewer for the helpful suggestion. In response, we have revised Figure 9 by replacing the previous corner plot with a line plot that includes shaded areas representing the standard deviation, thus providing a clearer visualization of variability across the data. We have also enhanced the overall quality of the figure. It can be seen now at the bottom of page 7.

Reviewer Point P2.8 — Terminology Refinement: Avoid using the term ”biomarker” unless biologically validated. ”Most important features” or ”predictive features” would be more appropriate in the context of machine learning feature selection.

Reply: We thank the reviewer for the valuable feedback regarding the use of the term ”biomarker”. We agree and we have implemented the suggested changes throughout the manuscript. Specifically, we have

replaced ”biomarker” with more appropriate terms such as ”predictive features,” ”microbial features,”, ”key attributes” and ”discriminative features” in the context of our machine learning feature selection. We have only retained the term ”biomarker” in general sections, such as the Introduction, Methods, Potential Implications and Conclusions, where we discuss the broader state of the art and the potential for selected features to be biologically or externally validated as true biomarkers in future research. This ensures that our terminology remains precise and contextually accurate.

Reviewer Point P2.9 — Relocate Table 7 Content: The discussion of specific taxa and Table 7 should be moved into the Results section, as it presents findings rather than interpretation.

Reply: We thank the reviewer observation. We have moved the content of Table 7 into the Analyses section (now table 5 at the top of page 8), where we now present the selected taxa and highlight the high genera variability observed in the results. However, we kept the interpretation and comparison with previously reported associations in the literature in the Discussion section, as we believe this context helps clarify the relevance of the findings.

Reviewer Point P2.10 — Reorganize the Discussion Section: A significant portion of the current discussion reads as results. These subsections should be moved to the Results section, and the Discussion should be condensed to focus on interpretation, implications, and broader context.

Reply: We appreciate the reviewer’s observation. In response, we have reorganized and condensed the Discussion section to avoid repeating points already presented in the Analyses section. We now focus more on interpreting the findings, exploring possible reasons behind the observed behavior of the methods, and situating our results within the context of relevant literature. These changes aim to enhance the clarity of the discussion.

Reviewer Point P2.11 — Add a Study Design Schematic: A clear, concise diagram summarizing the study design, including data processing, transformation, modeling, and evaluation steps, would greatly enhance reader comprehension.

Reply: We thank the reviewer for the suggestion. We have incorporated a graphical abstract into our submission, which illustrates the key steps of our study design, including data processing, normalization, feature selection, classification, and evaluation. We believe this addition significantly enhances the clarity and comprehension for the reader.

Reviewer Point P2.12 — Summarize Feature Importance Methods: Consider summarizing the feature importance techniques in a dedicated table to facilitate comparison and improve readability.

Reply: We thank the reviewer for this suggestion. We have incorporated a dedicated table (Table 8 at the top of page 12) summarizing the feature importance techniques to provide readers with a quick yet informative overview of the methods.

Reviewer Point P2.13 — Expand Method Descriptions: Briefly introduce each classification method (e.g., logistic regression, random forest, SVM) in the main text and provide a more detailed description in the Methods section.

Reply: We appreciate rhe reviewer’s valuable comment. We have now addressed this by briefly introducing the classifiers in the main text, as suggested.

”These models represent a diverse set of classification strategies. Random Forest and XGBoost are tree-based ensemble methods known for their robustness and ability to capture complex non-linear relationships. Logistic Regression is a linear model that provides interpretable coefficients and performs well when the data is linearly separable. Support Vector Machine is a margin-based classifier effective in high-dimensional spaces, particularly suitable for sparse data. Finally, K-Nearest Neighbor is a lazy learning method that classifies samples based on similarity to their neighbors, though it can be sensitive to noise and sparsity in the data. A more detailed description of each classifier is provided in the Methods section.”

Furthermore, we have dedicated subsections within the Methods section to provide a more in-depth description of each classifier, ensuring a comprehensive understanding for the reader.

Editorial Manager®

Cookie Preference Center

We use cookies which are necessary to make our site work. We may also use additional cookies to analyze, improve and personalize our content and your digital experience. For more information, see our Cookie Policy.

You may choose not to allow some types of cookies. However, blocking some types may impact your experience of our site and the services we are able to offer. See the different category headings below to find out more or change your settings.

Allow all

Manage Consent Preferences

Strictly Necessary Cookies

Always active

These cookies are necessary for the website to function and cannot be switched off in our systems. They are usually only set in response to actions made by you which amount to a request for services, such as setting your privacy preferences, logging in or filling in forms. You can set your browser to block or alert you about these cookies, but some parts of the site will not then work.

Functional Cookies

These cookies enable the website to provide enhanced functionality and personalisation. They may be set by us or by third party providers whose services we have added to our pages. If you do not allow these cookies then some or all of these services may not function properly.

Performance Cookies

These cookies allow us to count visits and traffic sources so we can measure and improve the performance of our site. They help us to know which pages are the most and least popular and see how visitors move around the site.

Targeting Cookies

These cookies may be set through our site by our advertising partners. They may be used by those companies to build a profile of your interests and show you relevant adverts on other sites. If you do not allow these cookies, you will experience less targeted advertising.

Cookie List

Clear

Apply Cancel
Consent Leg.Interest

Confirm my choices

Powered by onetrus
