# Supplementary material for: Exploring the role of normalization and feature selection in microbiome disease classification pipelines
Source: Gigascience. 2025 Sep 2;14:giaf096. doi: 10.1093/gigascience/giaf096 (PMC12402773; doi:10.1093/gigascience/giaf096)
Supplement: giaf096_Reviewer_2_Report_Original_Submission — Zuzanna Karwowska -- 4/5/2025 [file giaf096_reviewer_2_report_original_submission.pdf]

Reviewer Report

Title: Exploring the role of normalization and feature selection in microbiome disease classification pipelines

Version: Original Submission **Date:** 4/5/2025

Reviewer name: Zuzanna Karwowska

Reviewer Comments to Author:

This manuscript addresses an important and timely topic concerning the impact of data transformation and feature selection techniques on machine learning classification of microbiome datasets. Given the growing interest in developing robust predictive models in microbiome research, especially for disease classification, this study is of high relevance. The paper is generally well-written and provides a comprehensive overview of commonly used preprocessing and modeling methods in the microbiome field. However, the manuscript would benefit from several substantial revisions to improve clarity, interpretability, and completeness:

1 Inclusion of Additional Transformations: The manuscript should include results using log-relative abundance (log-RelAb) and presence/absence (PA) transformations, as these are standard approaches in microbiome data preprocessing and their inclusion would improve the comprehensiveness of the analysis. Additionally, it would be valuable to evaluate the effect of rarefaction on model performance. Including rarefied datasets would allow the authors to assess how this commonly used normalization method influences classification outcomes and whether it interacts with transformation or feature selection strategies.

2 Simplify Figure 1 for Clarity: Figure 1 would be more effective if it presented a single boxplot per transformation method (e.g., CLR, RELAB) to facilitate simpler and more direct comparisons.

3 Improve Table 8 Presentation: Table 8 lacks sufficient description. Consider either improving the accompanying explanation or replacing it with a figure (e.g., boxplots) to visually highlight performance distributions. If a table is retained, bold the best-performing results for clarity.

4 Unify Boxplots in Figure 5: For better comparison, combine all boxplots in Figure 5 into a single panel and ensure the Y-axis scale is consistent across plots.

5 Format P-values Appropriately: Round p-values to a reasonable number of decimal places (e.g., three significant digits) for readability.

6 Enhance Figure Quality: Improve the resolution of all figures and ensure consistent text formatting across all graphical elements.

7 Revise Figure 9 Visualization: Instead of displaying a small plot, use a line plot with a shaded area representing standard deviation or confidence intervals to better convey variability.

8 Terminology Refinement: Avoid using the term "biomarker" unless biologically validated. "Most important features" or "predictive features" would be more appropriate in the context of machine learning feature selection.

9 Relocate Table 7 Content: The discussion of specific taxa and Table 7 should be moved into the Results section, as it presents findings rather than interpretation.

10 Reorganize the Discussion Section: A significant portion of the current discussion reads as results. These subsections should be moved to the Results section, and the Discussion should be condensed to focus on interpretation, implications, and broader context.
11 Add a Study Design Schematic: A clear, concise diagram summarizing the study design, including data processing, transformation, modeling, and evaluation steps, would greatly enhance reader comprehension.
12 Summarize Feature Importance Methods: Consider summarizing the feature importance techniques in a dedicated table to facilitate comparison and improve readability.
13 Expand Method Descriptions: Briefly introduce each classification method (e.g., logistic regression, random forest, SVM) in the main text and provide a more detailed description in the Methods section.
In my opinion, this paper is an important contribution to the machine learning community working with microbiome data. While the manuscript could benefit from improvements in clarity and readability, it holds significant value. The feature selection aspect is particularly strong and stands out as a major strength.

Methods

Are the methods appropriate to the aims of the study, are they well described, and are necessary controls included? Choose an item.

Conclusions

Are the conclusions adequately supported by the data shown? Choose an item.

Reporting Standards

Does the manuscript adhere to the journal's guidelines on minimum standards of reporting? Choose an item.

Choose an item.

Statistics

Are you able to assess all statistics in the manuscript, including the appropriateness of statistical tests used? Choose an item.

Quality of Written English

Please indicate the quality of language in the manuscript: Choose an item.

Declaration of Competing Interests

Please complete a declaration of competing interests, considering the following questions:

- Have you in the past five years received reimbursements, fees, funding, or salary from an organisation that may in any way gain or lose financially from the publication of this manuscript, either now or in the future?
- Do you hold any stocks or shares in an organisation that may in any way gain or lose financially from the publication of this manuscript, either now or in the future?
- Do you hold or are you currently applying for any patents relating to the content of the manuscript?
- Have you received reimbursements, fees, funding, or salary from an organization that holds or has applied for patents relating to the content of the manuscript?
- Do you have any other financial competing interests?
- Do you have any non-financial competing interests in relation to this paper?

If you can answer no to all of the above, write 'I declare that I have no competing interests' below. If your reply is yes to any, please give details below.

I declare no competing interests

I agree to the open peer review policy of the journal. I understand that my name will be included on my report to the authors and, if the manuscript is accepted for publication, my named report including any attachments I upload will be posted on the website along with the authors' responses. I agree for my report to be made available under an Open Access Creative Commons CC-BY license (http://creativecommons.org/licenses/by/4.0/). I understand that any comments which I do not wish to be included in my named report can be included as confidential comments to the editors, which will not be published.

Choose an item.

To further support our reviewers, we have joined with Publons, where you can gain additional credit to further highlight your hard work (see: https://publons.com/journal/530/gigascience). On publication of this paper, your review will be automatically added to Publons, you can then choose whether or not to claim your Publons credit. I understand this statement.

Yes Choose an item.
